# Pan-cancer Myc modulator that targets Myc-α-tubulin interaction to drive selective mitotic catastrophe

**DOI:** 10.1038/s41598-025-22011-4

**Published:** 2025-10-31

**Authors:** Jessica Teitel, Margaret Farah, Michele L. Dziubinski, Pil Lee, Andrew White, Alexander Sobeck, Jose Colina, John Takyi-Williams, Bo Wen, Elmar Nurmemmedov, Ivan Babic, Andre Monteiro da Rocha, Karan Bedi, Aaron Robida, Grace McIntyre, Takashi Hotta, Yinzhi Lin, Sreeja C. Sekhar, Ryoma Ohi, Analisa DiFeo

**Affiliations:** 1 Department of Pathology, University of Michigan, Ann Arbor, MI 48109 USA; 2 Rogel Cancer Center, University of Michigan, Ann Arbor, MI 48109 USA; 3 College of Pharmacy, University of Michigan, Ann Arbor, MI 48109 USA; 4 Therapeutic Systems Research Laboratories (TSRL), Inc, Ann Arbor, MI 48105 USA; 5 Pharmacokinetics Core, University of Michigan, Ann Arbor, MI 48109 USA; 6 CellarisBio, San Diego, CA 92121 USA; 7 Frankel Cardiovascular Center Cardiovascular Regeneration Core Laboratory, University of Michigan, Ann Arbor, MI 48109 USA; 8 Internal Medicine, Cardiology, University of Michigan, Ann Arbor, MI 48109 USA; 9 Department of Biostatistics, University of Michigan, Ann Arbor, MI 48109 USA; 10 Center for Chemical Genomics, University of Michigan, Ann Arbor, MI 48109 USA; 11 Department of Cell and Developmental Biology, University of Michigan, Ann Arbor, MI 48109 USA

**Keywords:** High-grade serous ovarian cancer, c-Myc, G2-M arrest, Mitotic catastrophe, α-tubulin, Cancer therapy, Gynaecological cancer, Oncogenes, Cell-cycle exit, Checkpoints, Cytokinesis, Mitosis, Mitotic spindle, Target validation

## Abstract

*MYC* overexpression is a well-established cancer vulnerability, yet direct therapeutic targeting of Myc remains a challenge. Here, we identify DL78 as a potent antimitotic agent with selective anticancer activity through its regulation of Myc. DL78 demonstrated broad efficacy by inhibiting growth across nine cancer types and significantly reducing tumor burden in an in vivo model of platinum-resistant high-grade serous ovarian cancer, with no overt toxicity. DL78 preferentially targets chromosomally unstable, *MYC*-overexpressing cancer cells, a hallmark of high-grade serous ovarian cancer. Mechanistically, DL78 exploits Myc’s role in mitotic entry by disrupting its interaction with α-tubulin, leading to sustained mitotic arrest, mitotic catastrophe, and apoptosis while sparing nonmalignant cells. This study establishes a novel paradigm for Myc-targeted therapy by introducing DL78, which induces cancer-selective mitotic catastrophe by disrupting Myc’s interaction with α-tubulin rather than its transcriptional activity.

**Supplementary Information:**

The online version contains supplementary material available at 10.1038/s41598-025-22011-4.

## Introduction

Ovarian cancer stands as a formidable challenge in the realm of gynecologic malignancies, characterized by its insidious onset and the absence of specific symptoms in its early stages, which often leads to late diagnosis and a poor prognosis. This is especially true for high-grade serous ovarian cancer (HGSC), the most common and aggressive subtype^1^. Despite advances in surgical techniques and chemotherapy regimens, ovarian cancer remains the sixth cause of cancer death in women^2^. This challenge is driven by three characteristics: lack of early screening measures, heterogeneous genetics, and chemoresistance. A well-defined characteristic of HGSC is a high degree of chromosomal instability, with nearly 50% of patients displaying homologous recombination deficiency^3^. More than 90% of HGSC patients harbor mutations in *TP53* and over 45% of patients have amplification of *MYC*, the master regulator oncogene^3^. Otherwise, there are few commonly recurrent genes, occurring at rates of 10% or less^4,5^. As one of the most heterogeneous cancers in regard to DNA copy number variations^4^, few key driver mutations have been identified that apply across patients. For these reasons, while most patients initially respond to platinum-based chemotherapy, 90% of these patients will succumb to chemoresistance^6,7^. Currently only four targeted therapies are approved for clinical use—PARP inhibitors, VEGF inhibitors, and antibody-drug conjugates trastuzumab deruxtecan against HER2 and Mirvetuximab soravtansine-gynx against folate receptor alpha—but have limited improvement in progression-free survival and overall survival^5,8^. Therefore, there is an urgent need to uncover novel therapies and targetable vulnerabilities to treat HGSC cancer.

To address this need, we previously used a profile-based drug-repositioning computational platform, DrugPredict. DrugPredict integrates disease-associated genes, mouse genome mutation phenotype data, and chemical/drug profiles from three public databases to conduct simultaneous in silico target-based and phenotypic screening across half a million compounds, including FDA-approved drugs to explore drug repurposing^9^. In our previous work, DrugPredict revealed that amiodarone, a class III antiarrhythmic, ranked comparably high to currently used ovarian cancer drugs^10^. Subsequent studies confirmed the efficacy of amiodarone, revealing that it induced apoptosis in numerous primary and cisplatin-resistant patient-derived HGSC lines^9^. Mechanistically, amiodarone’s anticancer effects were mediated through Myc degradation, as overexpression of a non-degradable mutant Myc (T58A) eliminated amiodarone’s ability to inhibit cell growth^10^. While amiodarone’s anti-cancer properties have been reported in various in vivo models^11–14^, its dose-limiting toxicities and long half-life raise concern^15,16^. Importantly, efficacy was specific to amiodarone’s structure, but not necessarily to its potassium channel pharmacology, as other class III antiarrhythmics did not show anti-tumor effects^10^. Consequently, in this study, amiodarone was employed as a chemical precursor to discover new compounds that retained the parent drug’s anticancer and Myc-modulating properties while enhancing its drug-like attributes.

Myc is an influential transcription factor that modulates expression of nearly 15% of the genome^17–19^ and is amplified in 33 different cancers^20^. While it is a relevant cancer target, it has been challenging to therapeutically inhibit due to its lack of both enzymatic activity and a small molecule binding pocket. Despite these challenges, scientific advances have led to discoveries of directly or indirectly targeting Myc by disrupting its transcriptional activity, with several preclinical studies occurring in ovarian cancer^7,21–23^. Alternatively, other approaches include leveraging the vulnerabilities of these cancer cells due to Myc-driven phenotypes such as genomic instability, altered mitotic spindle morphology, and increased sensitivity to antimitotic agents^24–26^. For example, targeting Aurora A or CDK1 has been shown to induce synthetic dosage lethality in high Myc-expressing cells^7,27–32^. Thus, herein, we describe the discovery of a unique Myc modulator that leverages Myc’s pivotal role in mitotic progression by disrupting its interactions with α-tubulin rather than its transcriptional activity and induces cancer-specific mitotic catastrophe.

## Results

### Structure similarity search identifies DL78 as a selective anti-cancer compound

**Fig. 1:**
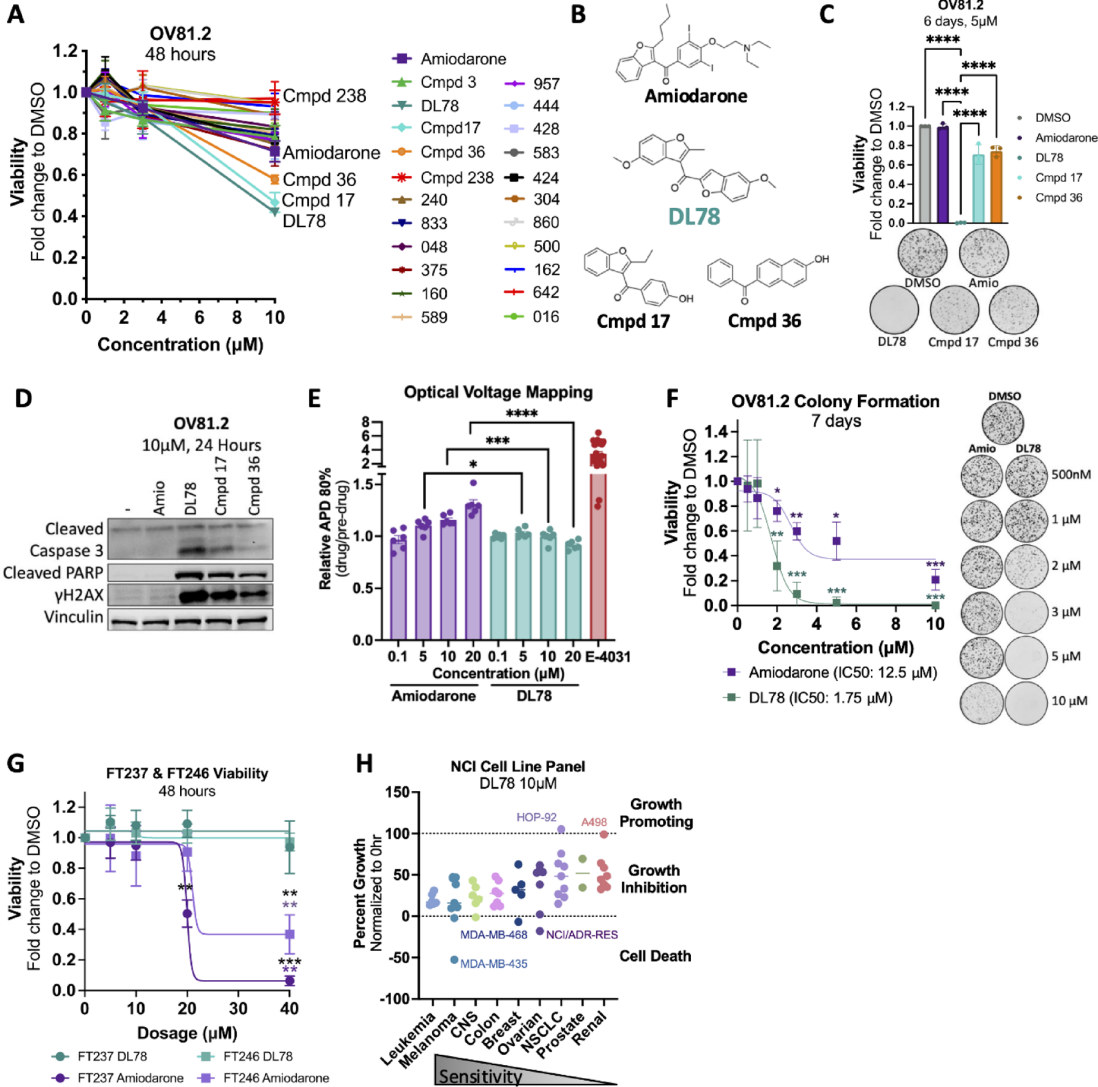
Utilizing structure similarity search to identify DL78, a selective anti-cancer compound. (**A**) 48-h CellTiter-Glo viability screen in OV81.2 treated with amiodarone or the 21 compounds identified from structure similarity search. Cells were treated at 1, 3, or 10 µM and compared to DMSO treated cells. *n* = 2 biological replicates. (**B**) Chemical structure of amiodarone, DL78, Cmpd 17, and Cmpd 36. (**C**) Colony formation in OV81.2 cells treated with DMSO, Amiodarone, DL78, Cmpd 17, or Cmpd 36 at 5 µM for 6 days. ∗∗∗∗*p* < 0.0001 as determined by one-way ANOVA. (**D**) Western blot in OV81.2 after 24 h of DMSO (−) or 10 µM treatment of amiodarone, DL78, Cmpd 17, or Cmpd 36. (**E**) Optical mapping measuring the relative Action Potential Duration (ADP) 80% repolarization in FCDI/iCell2 cardiomyocytes after 30 min treatment with amiodarone, DL78, or positive control E-4031 (500 nM). *n* = 5–24. (**F**) Colony formation in OV81.2 following treatment of amiodarone or DL78 (0, 500nM, 1, 2, 3, 5, 10 µM) over 7 days. (**G**) FT237 and FT246 MTT assay after 48 h of amiodarone or DL78 treatment (0, 5, 10, 20, 40 µM). Colored asterisks represent significance relative to DMSO; black asterisks represent significance between amiodarone and DL78. (**H**) Growth inhibition of DL78 in the NCI-60 Screen after 48-h 10 µM DL78 treatment. Data are normalized to untreated control and number of cells at 0 h. Cancer type is ordered by sensitivity to DL78. Data plotted as mean with error bars as standard deviation, *n* = 3 biological replicates. ∗*p* < 0.05, ∗∗*p* < 0.01, ∗∗∗*p* < 0.001, and ∗∗∗∗*p* < 0.0001 as determined by two-sided Student’s t-test.

Due to amiodarone’s toxic side effects, long half-life, and ability to block potassium channels such as the human Ether-à-go-go-Related Gene (hERG) channel, we sought to identify publicly available compounds that share amiodarone’s anticancer properties without its antiarrhythmic activity. Compounds were selected through a similarity search based on amiodarone, utilizing various modeling software, including Molecular Operating Environment (MOE), to predict the absence of blocking hERG channel activity, which is typically shown through prolongation of the action potential. Furthermore, it is well-established that the tertiary amine is responsible for amiodarone’s antiarrhythmic activity, while the iodine contributes to its toxicity^33^. Therefore, compounds lacking these chemical groups were prioritized. In total, 21 compounds (Cmpd) were screened in patient-derived HGSC cell line OV81.2 in a dose dependent fashion for 48 h to assess effects on viability (Fig. 1A). We identified three compounds (DL78, Cmpd 17, and Cmpd 36) that were significantly more potent than amiodarone at 5 and 10 µM (Figs. 1A,B and S1A). DL78 and Cmpd 17 retained the benzofuran of amiodarone with variations to the 3-acyl substituent, including truncation of the side chain as well as replacing the phenyl group with an additional benzofuran. Cmpd 36 replaced the benzofuran with a naphthalene and removed all substituents from the acyl phenyl group. Due to the observed plateau in the dose-response curve (Fig. S1A), colony formation assays were used to evaluate drug potency. Of note, potency values obtained from colony formation assays are expected to differ from those in short-term viability assay because the two methods probe distinct biological endpoints and use different drug-to-cell ratios. DL78 proved to be the most potent compound in colony formation assays (Fig. 1C), and it significantly induced apoptosis with 10 µM treatment as assessed by cleaved Caspase 3, cleaved PARP, and γH2AX (Fig. 1D). Next, we utilized optical voltage mapping to assess whether DL78 lacked the parent compound’s prolongation of action potential. In human terminally differentiated cardiomyocytes, amiodarone induced a dose-dependent prolongation of the action potential, whereas DL78 had no effect (Fig. 1E). Next, a dose-response was performed in a colony formation assay in OV81.2 to further demonstrate the potency of DL78 compared to amiodarone. DL78 had an IC50 under 2µM, whereas amiodarone required five times that dose for the same effect (Fig. 1F). Finally, it was crucial to validate that DL78 had selectivity towards cancer, rather than general toxicity. Given that ovarian cancer arises from the fallopian tube epithelium^34,35^, DL78 was tested in two nonmalignant fallopian tube cell lines, FT237 and FT246, as a “normal” control. DL78 treatment left FT237 and FT246 cells unaffected whereas amiodarone became toxic beyond 20 µM (Fig. 1G).

To evaluate pan-cancer activity, DL78 was tested at 10 µM in the NCI-60 Human Tumor Cell Line Screen, which includes 9 cancer types: breast, brain, colon, leukemia, melanoma, non-small cell lung, ovarian, prostate, and renal^36^. DL78 inhibited cell growth by ≥ 50% in 47 of the 60 cell lines (Fig. 1H), with leukemia and melanoma being the most sensitive. We next assessed whether the sensitivity of DL78 corresponded with expression of particular genes. Interestingly, there was a strong significant correlation between responsiveness to DL78 and increased *CCNE1* expression, which is amplified in 20% of HGSC and is associated with poor outcome^37^ (Fig. S1B). There was no significant correlation between DL78 response and *TP53* expression nor TP53 mutational status (Fig. S1C,D).

### DL78 sensitizes platinum-resistant cells to cisplatin and induces apoptosis following mitotic catastrophe

**Fig. 2:**
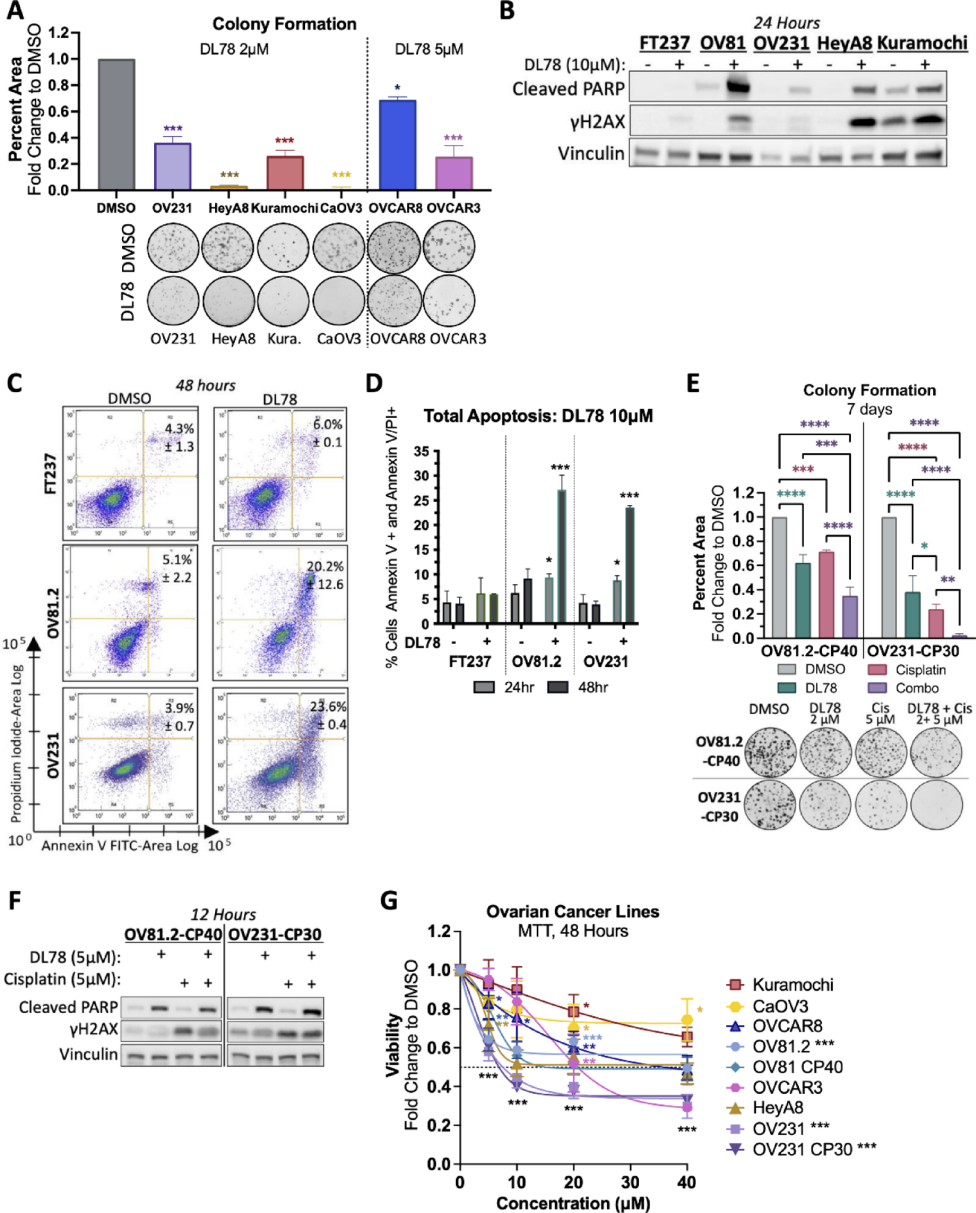
DL78 inhibits growth and induces apoptosis in primary and cisplatin-resistant ovarian cancer cells. (**A**) Colony formation over 7 days in OV231, HeyA8, Kuramochi, and CaOV3 (17 days) with 2 µM DL78 treatment or OVCAR8 and OVCAR3 with 5 µM DL78 treatment. Viability relative to DMSO treated cells. (**B**) Western blot in FT237, OV81.2, OV231, HeyA8, and Kuramochi after 24 h of 10 µM DL78 treatment. (**C**) Representative flow plots of Annexin V/PI of FT237, OV81.2, and OV231 cells treated with 10 µM DL78 for 48 h. Number in the top right quadrant is the average total apoptosis, calculated via summation of the top and bottom right quadrants. (**D**) Quantification of Annexin V/PI Flow Cytometry *n* = 3 via total apoptosis (early and late). 24-h treatment represented by the lighter color and 48-h treatment represented by the darker color. (**E**) Colony formation in cisplatin-resistant lines OV81.2-CP40 and OV231-CP30 following treatment of DMSO, 2 µM DL78, 5 µM Cisplatin, or a combination of 2 µM DL78 and 5 µM Cisplatin over 7 days. Viability relative to DMSO treated cells. Significance determined by one-way ANOVA. (**F**) Western blot in OV81.2-CP40 and OV231-CP30 treated with 5 µM DL78, Cisplatin, or both for 12 h. (**G**) MTT assay of Kuramochi, CaOV3, OVCAR8, OV81.2, OV81.2-CP40, OVCAR3, HeyA8, OV231, OV231-CP30 treated for 48 h with 0, 5, 10, 20, or 40 µM DL78. Three black asterisks on the key denote statistical significance at every dose. Three black asterisks on the graph denote statistical significance at that specific dose unless otherwise marked by cell-line specific color-matched asterisks. Data plotted as mean with error bars as standard deviation, *n* = 3 biological replicates. ∗*p* < 0.05, ∗∗*p* < 0.01, ∗∗∗*p* < 0.001, and ∗∗∗∗*p* < 0.0001 as determined by two-sided Student’s t-test.

HGSC is characterized by high genomic instability^4,38^. Among various gene amplifications, *MYC* and *CCNE1* are some of the most prevalent^3^. The significant correlations between responsiveness to DL78 and high DNA copy number or *CCNE1* expression further supported ovarian cancer as a suitable model to study DL78. We next determined DL78’s efficacy across a panel of HGSC cell lines, including an additional patient-derived line OV231, and commercial lines HeyA8, Kuramochi, CaOV3, OVCAR3, and OVCAR8. DL78 significantly decreased colony formation at 2 µM or 5 µM doses for 7 days in all cell lines tested (Fig. 2A). Observing the significant reduction in colony formation induced by DL78 treatment across 7 HGSC cell lines, we next explored its effects on cell death. OV81.2, OV231, HeyA8, and Kuramochi all showed increased cleaved PARP and γH2AX, with no significant induction in FT237 following 10µM DL78 treatment (Fig. 2B). Annexin V/PI staining confirmed apoptosis at 24 and 48 h in OV81.2 and OV231, while FT237 cells were unaffected (Fig. 2C,D, and S2). Given that most patients succumb to platinum-based chemoresistance, we also assessed whether DL78 was effective in isogenic lines OV81.2-CP40 and OV231-CP30, which are cisplatin-resistant relative to their parental lines as previously described^39,40^. DL78 decreased cell growth and significantly potentiated the effects of cisplatin in these lines (Fig. 2E). Furthermore, 12 h treatment of 5 µM DL78 led to significantly increased cleaved PARP (Fig. 2F).

**Fig. 3:**
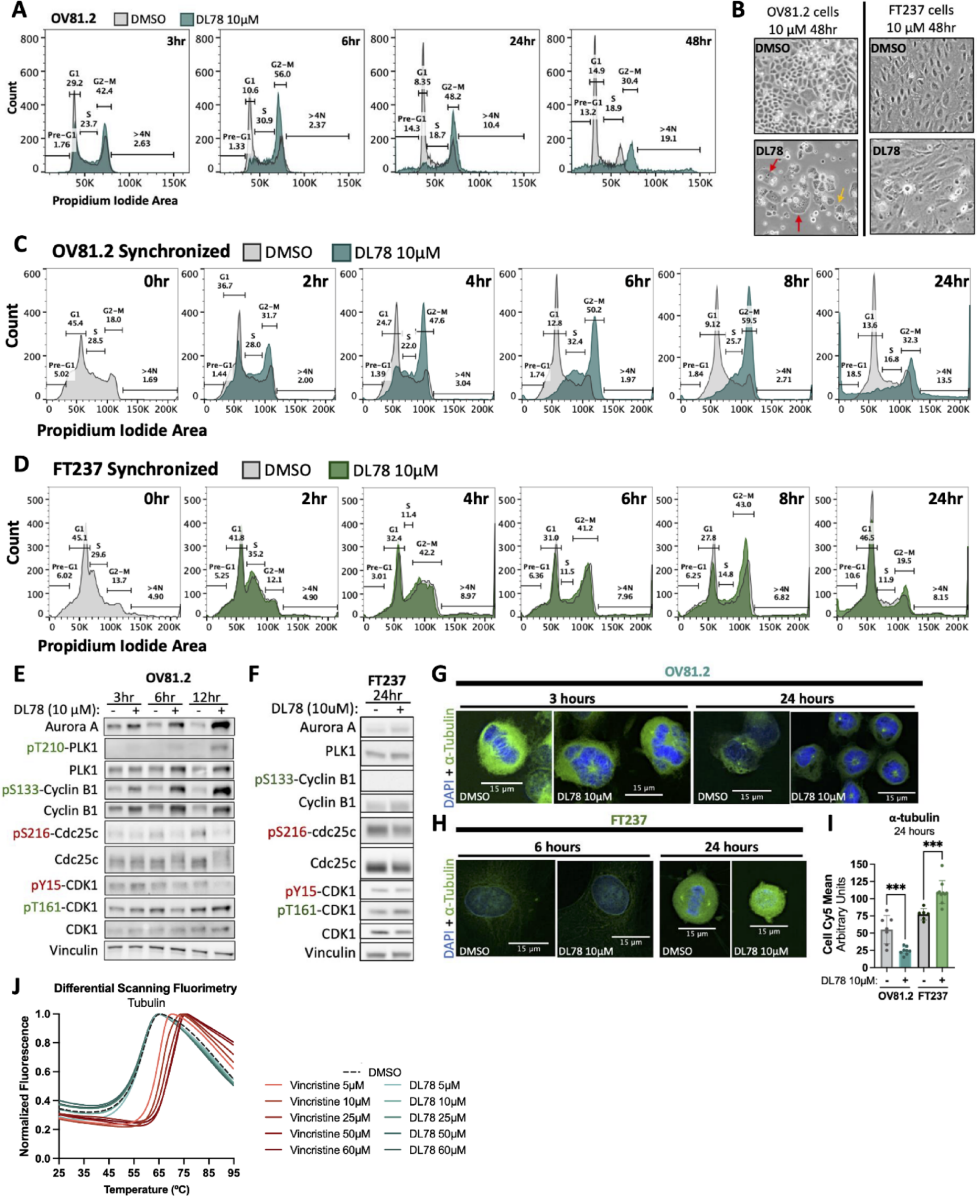
DL78 arrests cancer cells in mitosis, leading to mitotic catastrophe, while nonmalignant cells pause in G2 and recover. (**A**) Representative histograms of flow cytometry propidium iodide cell cycle analysis of OV81.2 treated with DMSO or 10 µM DL78 over 3, 6, 24, or 48 h. Statistics on graph are for DL78-treated cells. (**B**) Microscopy images at 10× magnification of OV81.2 or FT237 following 48 h of DMSO or 10 µM DL78 treatment. Colored arrows in OV81.2 treated cells mark enlarged, multinucleated cells (red arrow) and micronuclei (yellow arrow). (**C**) Representative histograms of flow cytometry propidium iodide cell cycle analysis with 10 µM DL78 over 3, 6, 24, or 48 h in double thymidine block synchronized OV81.2, or (**D**) double thymidine block synchronized FT237 cells. Statistics on graph are for DL78-treated cells. (**E**) Western blots after DMSO or 10 µM DL78 treatment over 3, 6, or 12 h in OV81.2 or (**F**) over 24 h in FT237. (**G**) Immunofluorescent microscopy images at 40× magnification of OV81.2 treated with DMSO or 10 µM DL78 at 3–24 h. Blue = DAPI, Magenta = α-tubulin. Scale bar is 20 μm. (**H**) Immunofluorescent microscopy images at 40× magnification of FT237 treated with DMSO or 10 µM DL78 at 6–24 h. Blue = DAPI, Magenta = α-tubulin. Scale bar is 20 μm. (**I**) Quantification of α-tubulin expression in OV81.2 (*n* = 7–8 mitotic cells) and FT237 (*n* = 6–10 mitotic cells) at 24 h via Cy5 mean. Data plotted as mean with error bars as standard deviation. ∗∗∗*p* < 0.001 determined by one-way ANOVA. (**J**) Differential scanning fluorimetry of 0.8 mg/mL tubulin in the presence of DMSO, Vincristine, or DL78 at 10–60 µM.

Interestingly, while a significant induction of apoptosis was observed upon DL78 treatment in all HGSC lines tested, there was a plateau in cell viability regardless of dose (Fig. 2G). To investigate this, cell cycle analysis in OV81.2 revealed that 10µM DL78 induced a G2-M arrest as early as 3 h, which peaked at 6 h, and was maintained through 24 h (Figs. 3A and S3A). By 48 h, cells died or became enlarged multinucleated cells with micronuclei (Fig. 3B, left), consistent with the phenotype of mitotic catastrophe, which is defined as premature or inappropriate entry into mitosis resulting in cell death^41–43^. The same G2-M arrest and mitotic catastrophe was also observed in OV231 cells (Fig. S3B–D). Remarkably, this phenotype is not seen in FT237 (Fig. 3B, right).

To further understand the kinetics of the G2-M arrest, cells were synchronized via a double thymidine block prior to 10 µM treatment with DL78. Synchronized cell cycle analysis highlighted that the cancer cells experienced a significant decrease in the G1 population within 2 h of DL78 treatment, without altering the duration of S phase (Figs. 3C, S3E,F). Beginning at 4 h, there was a significant increase in G2-M as the cells accelerated through the cell cycle and accumulated in a G2-M arrest, ultimately resulting in polyploidy and death by 24 h. Thus, cancer cells prematurely enter mitosis in the presence of DL78. Conversely, the nonmalignant cells only reached a significant decrease in G1 and slight increase in G2-M at 8 h (Figs. 3D and S3G). However, there was near complete recovery by 24 h, except for slight but significant changes in the S phase and Pre-G1 populations. Of note, the synchronization process increased the Pre-G1 population at baseline in the FT237 and as well as the DL78-induced Pre-G1 population in OV81.2.

To ascertain if either cell type obtained mitotic entry with DL78 treatment, the expression of proteins that regulate the G2/M transition were examined. In OV81.2, 10 µM DL78 treatment increased expression of Aurora A, PLK1, and Cyclin B1, which are known to be elevated in cells progressing to M phase^44^ (Figs. 3E and S3H). Activation of this pathway was evident due to increased phosphorylation of Threonine210-PLK1, suggesting active PLK1, which then phosphorylates Cyclin B1 at Serine133. This phosphorylation event is necessary for Cyclin B1 to enter the nucleus^45^, and within 6 h of DL78 treatment, phosphorylation of S133-Cyclin B1 significantly increased. By 12 h of DL78 treatment, phosphatase Cdc25c became activated through dephosphorylation at Serine216. A shift in Cdc25c’s molecular weight was also observed, likely due to increased activating phosphorylation at various sites in preparation for mitosis^46^. This Cdc25c activation leads to dephosphorylation of CDK1 at Tyrosine15 (Y15), an inhibitory phosphorylation site, activating CDK1 at 12 h (Fig. 3E), thus triggering an automated feedback loop that leads to irreversible mitotic entry^47^. Furthermore, we show that DL78 induced the same anticipated effects on G1/S proteins as Nocodazole, another small molecule that arrests cells in mitosis. Treatment decreased Cyclin E, E2F-1, and p21, while showing an increase in the ratio of phosphorylated T160-CDK2 as CDK2 switches from Cyclin E to Cyclin A2 (Fig. S3I), consistent with CDK2 phosphorylation remaining through mitosis^48^.

Since FT237 cells proliferate at a slower rate, we examined the expression of mitotic entry proteins at 24 h. In these nonmalignant cells, while there was a trending increase in Aurora A expression, 10 µM DL78 did not significantly increase expression of Aurora A, PLK1, nor Cyclin B1 (Figs. 3F and S3H). Furthermore, there was a significant decrease in phosphorylation of S133-Cyclin B1, suggesting Cyclin B1 remained cytoplasmic. Total Cdc25c did not experience a band shift, nor did CDK1 become dephosphorylated at Y15; instead there was a significant increase in phosphorylation of Threonine161-CDK1, which helps protect premature activation of the CDK1:Cyclin B1 complex^49^. Altogether, this suggests that the FT237 cells remained in G2 and did not reach mitotic entry. Thus, these studies uncover that DL78 induces selective uncoordinated mitotic entry in cancer cells to drive mitotic catastrophe, while sparing nonmalignant cells.

Due to DL78’s robust impact on mitosis, we next examined its effect on microtubules. Immunofluorescence microscopy revealed that within 3 h of 10 µM DL78 treatment, OV81.2 cells were impaired in their ability to form a proper mitotic spindle. Microtubule structures that did form were either short spindles or small asters that failed to separate (Fig. 3G). Expression levels of α-tubulin decreased, and the asters persisted in an aberrant state through 24 h. Interestingly, nonmalignant FT237 cells did not exhibit discernible alterations in spindle morphology following a 6 h treatment with DL78 and at 24 h, these cells appeared in G2, noted by decondensed chromosomes and depolymerized α-tubulin (Fig. 3H). Thus at 24 h, α-tubulin significantly decreased in dividing OV81.2 cells, whereas dividing FT237 cells had a significant increase (Fig. 3I), highlighting that microtubule formation is selectively impacted in the cancer cells. Decreased α-tubulin expression was also observed by Western blot in OV81.2 as early as 12 h (Fig. S3J). Interestingly, these results are consistent with computational analysis from the COMPARE pattern recognition algorithm, a product of the NCI-60 Screen, that correlates a compound of interest to all other 88,000 + compounds tested^50,51^. The algorithm showed that DL78 significantly correlated with other microtubule targeting drugs Bavistin/carbendazim, visoltricin, indibulin, and clanfenur (*r* > 0.64)^52–54^ as well as microtubule stabilizers, paclitaxel and docetaxel (*r* > 0.4, *r* > 0.5)^55,56^ (Table S1). To determine if DL78 directly prevents microtubule assembly, we performed a microtubule pelleting assay. Tubulin was assembled in the presence or absence of DL78 (2.5–160µM), and then subjected to ultracentrifugation. Our results show that DL78 does not affect microtubule formation directly (Fig. S3K). Further, we confirmed through differential scanning fluorimetry that DL78 does not alter tubulin’s stability in the presence of a thermal challenge, suggesting that tubulin is not a target of DL78 (Figs. 3J and S3L). Thus, other proteins may be involved in driving the spindle assembly defects induced by this antimitotic agent.

### DL78 modulates Myc

**Fig. 4:**
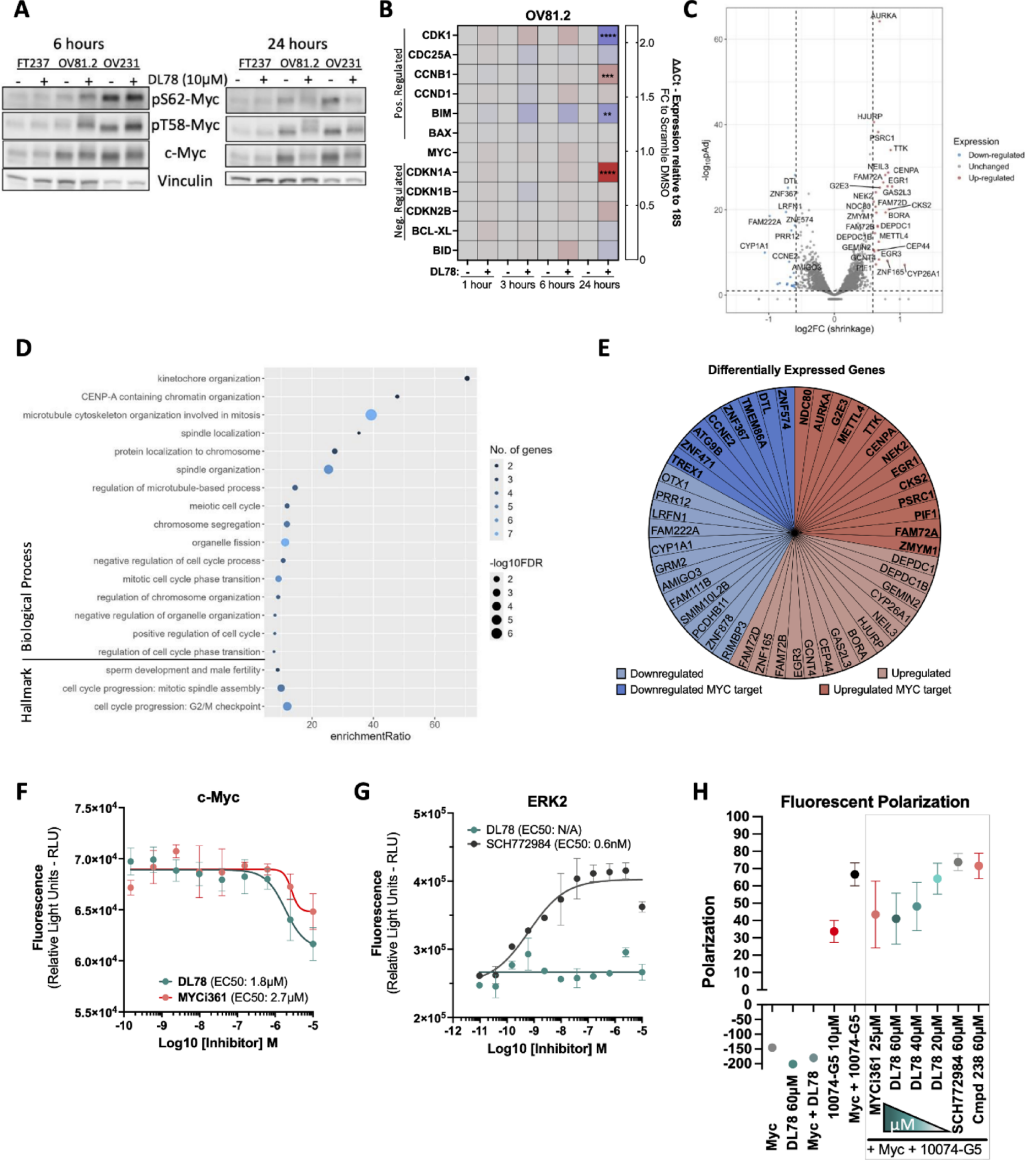
DL78 modulates Myc. (**A**) Western blot of Myc in FT237, OV81.2, and OV231 treated with DMSO or 10 µM DL78 for 6–24 h. (**B**) qPCR analysis of OV81.2 after 10 µM DL78 treatment over time (1, 3, 6, 24 h) relative to DMSO treated cells at the same timepoints. Positively regulated genes on top, followed by negatively regulated genes, and non-Myc target gene *BID*. (**C**) Volcano plot of differentially expressed genes from RNASeq analysis of OV81.2 treated with 10 µM DL78 for 6 h. (**D**) WebGestalt analysis of the Biological Process and Hallmark gene ontologies identified from the significantly upregulated genes via RNASeq. (**E**) Chart of significantly differentially expressed genes identified in RNASeq, color coded by downregulation (blue) or upregulation (red). Myc target genes are shaded and bolded. (**F**) Florescent readout of the Myc-MicroTag in HEK293 cell lysate treated with DL78 or MYCi361 over a dose curve (10 µM to 0.4 nM) for 30 min. (**G**) Florescent readout of the ERK2-Micro-Tag in HEK293 cell lysate treated with DL78 or SCH772984 over a dose curve (10 µM to 0.01 nM) for 30 min. (**H**) Fluorescence polarization competition assay of 10 µM 10,074-G5 incubated with 10 µM recombinant Myc and test compounds at various concentrations for 30 min. Data plotted as mean with error bars as standard deviation, *n* = 3 biological replicates. ∗*p* < 0.05, ∗∗*p* < 0.01, ∗∗∗*p* < 0.001, and ∗∗∗∗*p* < 0.0001 as determined by two-sided Student’s t-test.

Given that the efficacy of the parent compound, amiodarone, relies on Myc degradation ​^10^, and considering Myc’s critical role in regulating cell proliferation​, sensitizing cells to antimitotic agents^24–26^, and influencing microtubule nucleation and organization ​^24–26^, as well as the ability of microtubule-targeting drugs to impact Myc activity​^57–59^, we investigated whether the cancer-specific antimitotic effects of DL78 were driven by Myc modulation. Remarkably, we found that Myc protein expression, including its phosphorylation site, Threonine-58 (T58), which aids in regulating Myc’s stability^60^, were significantly affected upon DL78 treatment. DL78 treatment led to increased phosphorylation at T58-Myc within 6 h in OV81.2 and OV231 (Figs. 4A and S4A,B), which peaked at 12 h (Fig. S4C) and led to total Myc protein loss by 24 h in a proteosome-dependent manner without affecting the rate of its degradation (Fig. S4D,E). Additionally, there was a dose-dependent effect in the loss of Myc (F(3,16) = 6.404; *p* = 0.0047) and trending in phosphorylation of T58 (F(3,16) = 3.045; *p* = 0.0592) (Fig. S4F). Phosphorylation of Serine62-Myc (S62) was unchanged through 12 h at 10µM (Fig. S4C). The nonmalignant cells, which have lower Myc expression at baseline, did not experience any changes in Myc phosphorylation nor total protein expression when treated with DL78 (Figs. 4A and S4A,B). Next, we examined expression of Myc target genes, in which significant changes weren’t observed until 24 h of treatment with 10 µM DL78, which was consistent with the loss of Myc protein at this timepoint. These changes included decreased expression of Myc target genes *CDK1* and *BIM*, and increased expression of *CDKN1A* (Fig. 4B). Interestingly, *CCNB1* remained elevated, potentially because of the G2-M arrest. The *MYC* transcript, however, was unchanged. Furthermore, to assess the impact of DL78 on Myc transcriptional activity, we ascertained binding to the E-box sequence (CACGTG) in OV81.2 and FT237 DL78-treated lysates. These studies showed that baseline Myc transcriptional activity is decreased in the nonmalignant FT237 cells relative to cancer cells, and upon DL78 treatment, remained unchanged (Fig. S4G). Interestingly, in OV81.2, a significant increase in Myc-E-box binding is observed at 6 h of 10 µM DL78, but this returns to untreated levels by 24 h (Fig. S4G), suggesting that Myc maintains its transcriptional activity in the presence of DL78.

RNA-Seq analysis was conducted to evaluate the global transcriptomic changes triggered by DL78 treatment within six hours. DL78 treatment resulted in 47 significantly differentially expressed genes (27 upregulated and 20 downregulated) (Fig. 4C and S4H). Consistent with the biological effects observed in vitro, we found that the upregulated genes showed enrichment for biological processes such as microtubule cytoskeleton organization in mitosis, kinetochore organization, and spindle organization as well as Hallmark G2/M checkpoint (Fig. 4D). In addition, 48% of the upregulated genes are known Myc target genes, and 9 of those 13 genes are expected to be activated by Myc (Fig. 4E). While the 20 significantly downregulated genes did not display pathway enrichment, 40% were Myc target genes (Fig. 4E). This suggests that Myc is still active as a transcription factor at this early timepoint, which aligns with the E-box assay. Target gene expression only decreases at 24 h when total Myc is lost, and cells enter apoptosis.

After observing the robust early changes in Myc phosphorylation and ultimate loss of the Myc protein, we next examined whether DL78 directly or indirectly interacted with Myc. For these studies, we used MYCi361 as a positive control, which has been shown to directly bind to Myc, alter its phosphorylation, and decease transcriptional activity^61^. Both compounds displayed similar effects on colony formation, but MYCi361 showed greater potency in a cell viability assay (Fig. S5A,B), most likely because this compound does not induce the robust G2/M arrest observed with DL78. However, treatment with DL78 led to significantly higher induction of T58-Myc phosphorylation, greater loss of total Myc, and greater cleaved PARP and γH2AX at 24 h than MYCi361 (Fig. S5C). Next, these compounds were tested in a target engagement assay using the Micro-Tag platform, an in-cell thermal challenge fluorescent-based readout. This assay measures drug-protein engagement in cells by quantifying shifts in protein thermal stability after preincubation with the drug via a fluorescence resonance energy transfer readout. The aggregation temperature of Myc was empirically determined to be 53 °C (Fig. S5D). Within 30 min, DL78 altered Myc’s stability with an EC50 of 1.8 µM compared to MYCi361 with an EC50 of 2.7 µM (Fig. 4F), which matched previous findings^61^. Neither compound affected negative control Gapdh (Fig. S5E,F). Given that ERK is known to directly bind and phosphorylate Myc at S62, which must occur for T58 to become phosphorylated^62^, we next determined whether DL78 interacted with ERK2. The aggregation temperature of ERK2 was empirically determined (Fig. S5G). Positive control SCH772984, a known ERK2 inhibitor with high specificity, displayed an EC50 of 0.6 nM while DL78 had no effect on protein stability (Fig. 4G). Thus, DL78 engages Myc-containing complexes, without engaging another Myc-interacting protein, ERK1/2. This was further confirmed by the failure of DL78 to affect ERK1/2 kinase activity (Fig. S5H). Furthermore, phosphorylation of ERK1/2 and GSK3β, a kinase that phosphorylates Myc at T58, showed no difference in activation as measured by the ratio of phosphorylated protein to total protein in the DL78-treated cells over time (Fig. S5I,J).

**Fig. 5:**
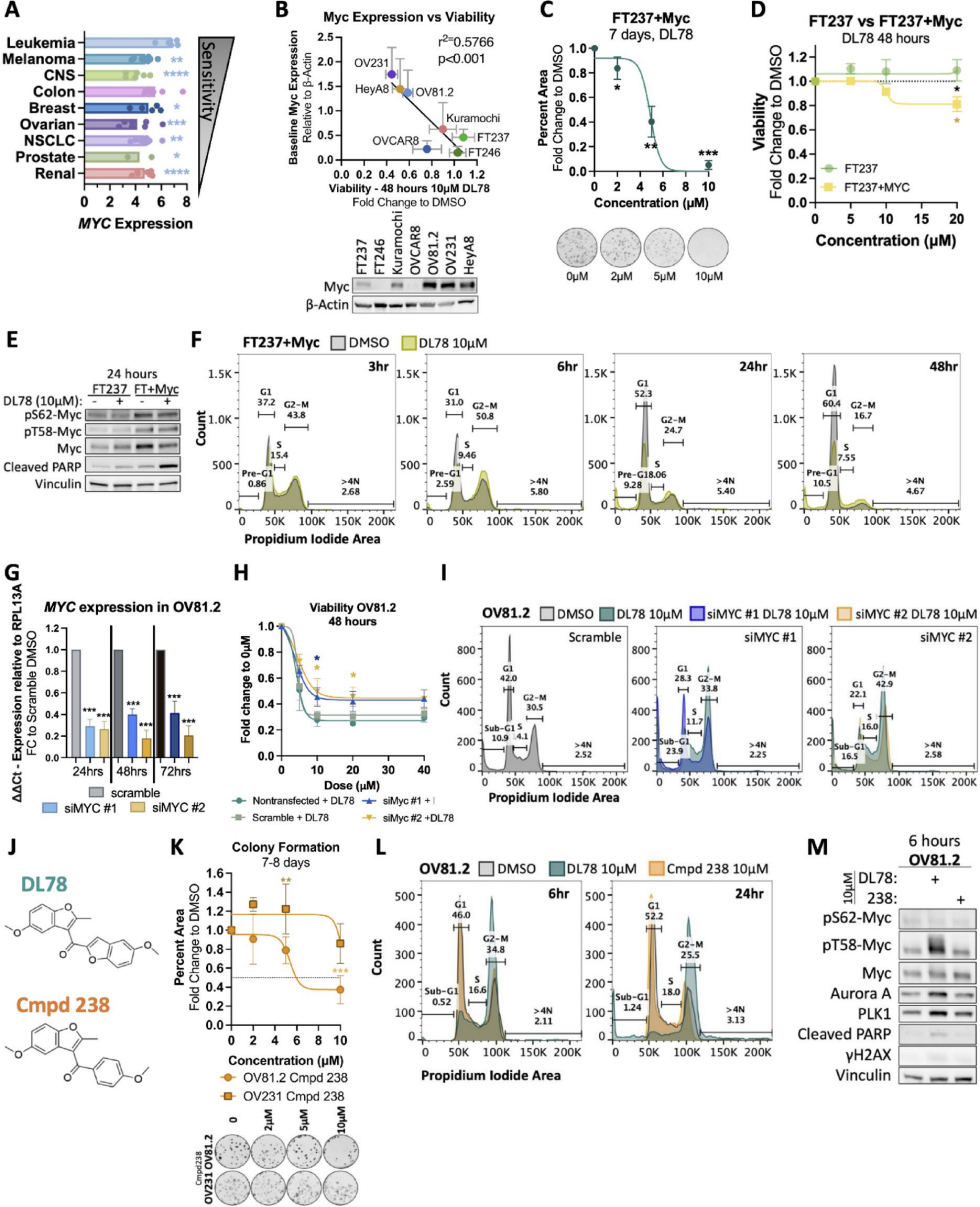
DL78’s dependency on Myc-driven cancer cells. (**A**) Myc mRNA expression from RNASeq of the cell lines in the NCI-60 Screen. Significance determined by two-way ANOVA. (**B**) Correlation of Myc protein expression (representative Western shown below) with cell viability after 10 µM DL78 treatment for 48 h relative to DMSO. (**C**) Colony formation in FT237 + Myc with 0, 2, 5, or 10µM treatment over 7 days. (**D**) MTT in FT237 and FT237 + Myc cells after 48 h of DL78 treatment. Colored asterisk denotes significantly different from 0 µM. Black asterisk denotes significant difference between cell lines at that dose. (**E**) Western blot in FT237 and FT237 + Myc cells treated with 10 µM DL78 for 48 h. (**F**) Representative histograms of flow cytometry propidium iodide cell cycle analysis with 10 µM DL78 over 3, 6, 24, or 48 h in FT237 + Myc cells. Statistics on graph are for DL78-treated cells. (**G**) qPCR analysis of *MYC* in OV81.2 following transfection with scramble, siMYC#1 or siMYC#2 for 24, 48, or 72 h. (**H**) MTT in OV81.2 cells transfected with scramble, siMYC#1 or siMYC#2 following 48 h treatment of DL78 at 5, 10, 20, or 40 µM. Colored asterisk denotes significantly different from treated scramble transfected cells. (**I**) Representative histograms of flow cytometry propidium iodide cell cycle analysis with 10 µM DL78 over 6 h in OV81.2 cells transfected with scramble, siMYC#1, or siMYC#2. Statistics on graph are for siMYC DL78-treated cells. (**J**) Chemical structures of DL78 and Cmpd 238. (**K**) Colony formation with Cmpd 238 treatment over 7 days in OV81.2 (circles) or 9 days in OV231 (squares). (**L**) Representative histograms of flow cytometry propidium iodide cell cycle analysis of OV81.2 cells treated with 10 µM DL78 or Cmpd 238 for 6 or 24 h. Statistics on graph are for Cmpd-238-treated cells. (**M**) Western blot of Myc, apoptotic, and cell cycle proteins in OV81.2 cells treated with 10 µM DL78 or Cmpd 238 for 6 h. Data plotted as mean with error bars as standard deviation, *n* = 3 biological replicates. ∗*p* < 0.05, ∗∗*p* < 0.01, ∗∗∗*p* < 0.001, and ∗∗∗∗*p* < 0.0001 as determined by two-sided Student’s t-test.

To validate the findings from the Micro-Tag assay and determine whether DL78 binds directly to Myc in a cell-free system, a competitive fluorescence polarization assay was performed using recombinant Myc protein and 10074-G5, a fluorescent small molecule which is known to bind to Myc at the basic helix-loop-helix leucine zipper (bHLH-ZIP) domain. This assay mirrors the approach used by Han et al.^61^ to confirm MYCi361 binding to Myc. Notably, neither Myc nor DL78 fluoresce (Fig. 4H), ensuring that the results remain unaffected. 10074-G5 was incubated with 10 µM Myc and 20, 40, and 60 µM DL78. DL78 competed with 10074-G5 in a dose dependent manner, obtaining a similar reduction of florescence polarization as MYCi361 (25 µM), used as a positive control (Fig. 4H). ERK inhibitor SCH772984 and Cmpd 238, a chemically inactive DL78 analog further described in Fig. 5, had no effect even at 60 µM. Altogether, these data highlight that DL78 interacts with Myc without affecting the kinases that regulate Myc’s stability or Myc’s transcriptional activity until the protein is lost at 24 h, coinciding with the onset of cell death. Further, since MYCi361 is known to bind to Myc at a site similar to 10074-G5, our data suggest that DL78 may either bind to a similar site in the C-terminus or interact elsewhere, inducing a conformational shift that displaces 10074-G5.

### High Myc-expressing cancer cells are selectively sensitive to DL78

Given that DL78 was shown to interact with Myc, we sought to investigate if DL78 relied on the presence of Myc. First, we examined whether Myc expression correlated with DL78 sensitivity. Returning to the NCI-60 Screen data, RNASeq analysis revealed that the leukemia lines, which were most sensitive to DL78, had on average significantly higher *MYC* expression than 7 other cancers, excluding colon (Fig. 5A). Furthermore, a strong significant correlation was found between Myc protein expression and cell viability in all HGSC cell lines and nonmalignant fallopian tube cell lines tested (Fig. 5B and S6A).

To probe if DL78’s efficacy required Myc expression, we induced *MYC* expression in nonmalignant cells to determine whether this would sensitize them to DL78. Overexpression of *MYC* in the insensitive FT237 resulted in significant inhibition of colony formation with an IC50 of 6µM (Fig. 5C) as well as a significant decrease in cell viability (Fig. 5D). Furthermore, cleaved PARP was significantly induced with 10 µM treatment of DL78 only in the FT237 overexpressing *MYC* and not the parental FT237 cells (Figs. 5E and S6B). Similar to what was found in the cancer cells, FT237 + Myc cells experienced a significant increase in pT58-Myc/Myc ratio following 24 h of treatment, as well as a significant G2-M arrest at 6 h which resulted in cell death (Figs. 5F and S6C). Interestingly, no phenotypic changes consistent with mitotic catastrophe were observed in DL78-treated FT237 + Myc cells (Fig. S6D), which may be due to the lack of genomic instability in these cells given that they are not fully transformed. It has been shown that *MYC* overexpression in hTERT-immortalized fallopian tube cells is not sufficient to gain levels of chromosomal instability comparable to cancer^63,64^. Thus, these cells are not vulnerable to Myc-induced mitotic catastrophe but instead engage growth suppressive and apoptotic machinery. Next, to further validate the dependency on Myc expression, knockdown experiments using two independent siRNAs targeting Myc were performed in OV81.2 cells. With only 50% knockdown achieved, likely caused by the cancer cells’ dependence on Myc, there was a significant decrease in efficacy with DL78 as shown by a viability assay (Fig. 5G,H) along with a significant rescue of the G2-M arrest (Figs. 5I and S6E). Importantly, Myc knockdown alone did not alter the cell cycle, with the exception of increased cell death (Fig. S6F,G). These studies demonstrate that DL78 requires oncogenic Myc to induce the G2-M arrest in cancer cells.

We assessed DL78’s chemical specificity towards Myc using an inactive analog, Cmpd 238. In the original lead compound screen, we identified Cmpd 238, which has a similar structure to DL78, but replaces the acyl benzofuran with an acyl phenyl and lacks biological activity (Fig. 5J). Thus, we sought to include it as a negative control to help confirm DL78’s structural specificity. Cmpd 238 induced modest cell death at 10 µM in OV81.2 and required as high as 40 µM in OV231 to significantly reduce viability (Fig. S6H,I). When tested in a colony formation assay, significant cell death was not reached until 10 µM in OV81.2, while OV231 remained unaffected (Fig. 5K), compared to 5 µM DL78 treatment which completely killed both lines. Cmpd 238 did not affect cell cycle (Figs. 5L and S6J) nor result in increased expression of cell cycle or apoptotic proteins (Figs. 5M and S6K). Finally, Cmpd 238 did not alter phosphorylation of Myc nor its total expression, highlighting DL78’s structural specificity. This lack of activity is consistent with the fluorescence polarization assay, which showed that Cmpd 238 did not bind to Myc (Fig. 4H). Overall, these data combined suggest that DL78 is dependent on Myc, and its chemical structure uniquely interacts with Myc.

### DL78 disrupts Myc and α-tubulin interaction

**Fig. 6:**
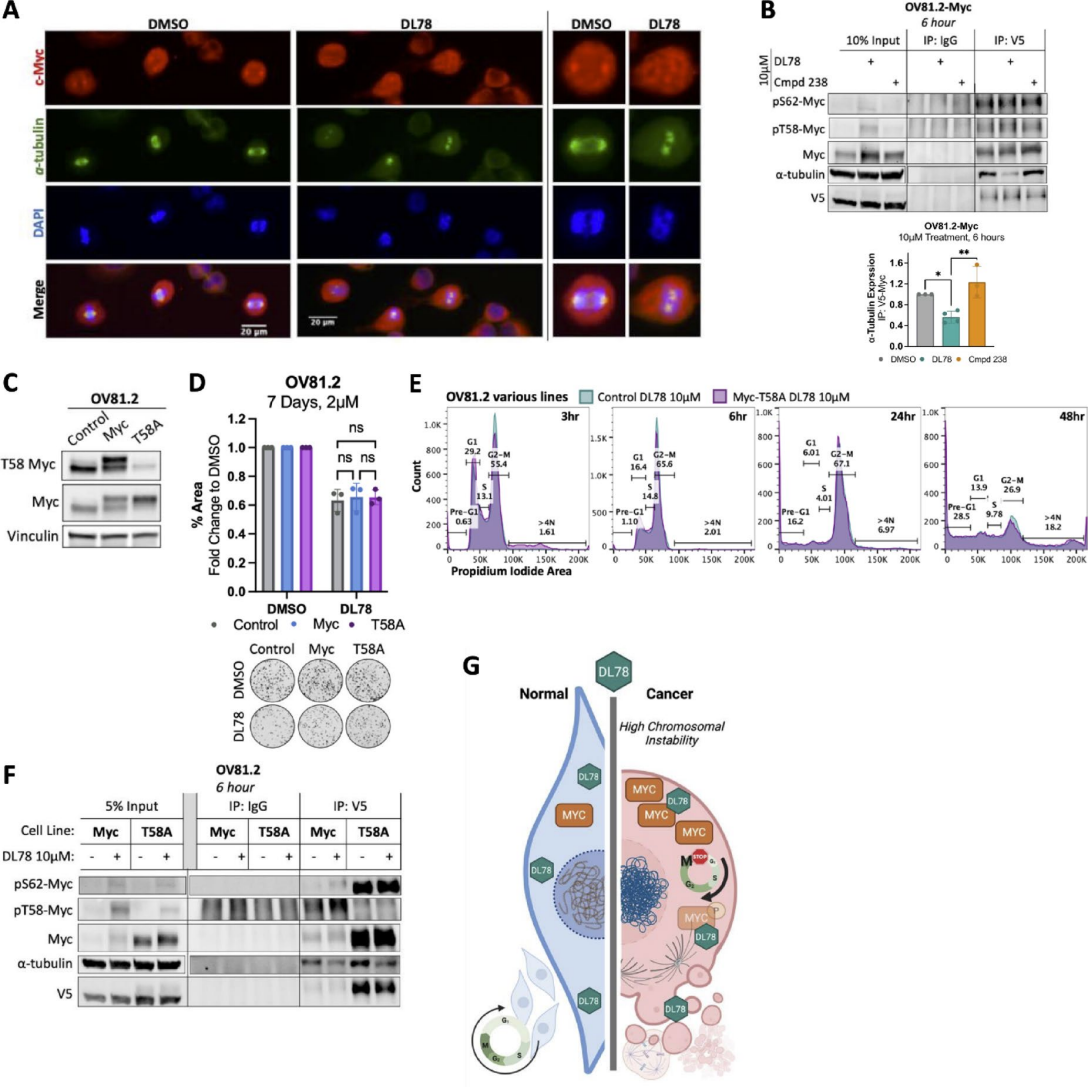
DL78 disrupts Myc-α-tubulin interaction. (**A**) Immunofluorescent microscopy at 40× magnification of OV81.2 cells synchronized via double thymidine block, then upon final release, treated with DL78 10 µM for 4 h. Myc (red), α-tubulin (green), DAPI (blue). Scale bar is 20 μm. Zoomed images on the right. (**B**) Co-immunoprecipitation of V5-tagged Myc from OV81.2-Myc cells treated with DMSO, 10 µM DL78, or 10 µM Cmpd 238 for 6 h. Quantification below. a-tubulin input was developed independently of the immunoprecipitation lanes. (**C**) Western blot of OV81.2 Control, Myc, or T58A transduced cells. (**D**) Colony formation in OV81.1 Control, Myc or T58A cells following 7 days of DMSO or 2 µM DL78 treatment. Representative images are below graph. (**E**) Representative histograms of flow cytometry propidium iodide cell cycle analysis with 10 µM DL78 treatment over 3, 6, 24, or 48 h in OV81.2 Control or T58A. DMSO treatment shown in Fig. S7L. (**F**) Co-immunoprecipitation of V5-tagged Myc from OV81.2-Myc or OV81.2-Myc-T58A cells treated with DMSO or 10 µM DL78 6 h. pS62 and a-tubulin input were developed independently of the immunoprecipitation lanes. (**G**) Schema of DL78’s mechanism of action and differential responses in the nonmalignant versus cancer cells. Schematic made in Biorender. https://BioRender.com/01wlea5. Data plotted as mean with error bars as standard deviation, *n* = 3 biological replicates. ∗*p* < 0.05, ∗∗*p* < 0.01, ∗∗∗*p* < 0.001, and ∗∗∗∗*p* < 0.0001 as determined by two-sided Student’s t-test.

Finally, we sought to connect DL78’s effects on Myc with the observed microtubule defects. *MYC* overexpression can alter proper mitotic spindle formation through improper chromosomal alignment, decreased microtubule aster length, and decreased centrosome distance^24^. Furthermore, Myc is known to interact with α-tubulin for stabilization, and disruption of this interaction contributes to the efficacy of several microtubule destabilizers^58,59^. We hypothesized that DL78 could disrupt Myc’s interaction with α-tubulin, leading to phosphorylation at T58-Myc and its subsequent degradation. Therefore, we examined the colocalization of Myc and α-tubulin in synchronized cells. We found that in untreated cells, Myc was identified at the microtubule asters with clear puncta (Fig. 6A). After 6 h of 10µM DL78 treatment, while some Myc still colocalized with the multiple abnormal microtubule asters, there were numerous Myc aggregates throughout the cell (Fig. 6A). To investigate the impact of DL78 on the binding between Myc and α-tubulin, we performed a coimmunoprecipitation assay. Upon treatment with DL78 at 10 µM for 6 h, binding of α-tubulin to Myc was reduced by greater than 50% (Fig. 6B). The inactive DL78 analog, Cmpd 238, as well as the Myc inhibitor MYCi361, did not alter α-tubulin and Myc binding (Fig. S7A), highlighting DL78’s specificity and its distinction from other Myc-binding compounds.

We next sought to evaluate the role of T58 phosphorylation of Myc in DL78’s efficacy. First, we confirmed whether DL78-mediated T58-Myc phosphorylation occurred in other cancer types. We used non-small cell lung cancer cell lines A549 and NCI-H1975, which displayed differential sensitivity to DL78. A549 was significantly more sensitive to DL78 at 10 µM whereas NCI-H1975 was resistant (Fig. S7B). We found that only A549 had increased phosphorylation of T58-Myc at 6 h, while it remained unchanged in NCI-H1975 (Fig. S7C). Furthermore, NCI-H1975 did not undergo the robust G2/M arrest or mitotic catastrophe seen in all other cancer cells. Instead, NCI-H1975 experienced a decrease in G1 and a slight but significant increase in G2-M at 6 h, but ultimately did not reach a prolonged G2-M arrest, polyploidy, nor significant induction of cleaved PARP (Fig. S7D–F). Thus, the DL78-induced T58-Myc phosphorylation is not unique to ovarian cancer.

To directly investigate if DL78-induced T58-Myc phosphorylation is a cause or effect of the α-tubulin separation, we transduced OV81.2 and OV231 cells to overexpress wild-type Myc and mutant Myc-T58A, which cannot be phosphorylated at T58. After confirming overexpression via Western blotting (Figs. 6C and S7G) and no effect on cell cycling (Fig. S7H), a colony formation assay and cell cycle profiling were performed. DL78 had the same effect on growth inhibition across all conditions (Figs. 6D and S7I). Similarly, cell cycle profiling revealed that that overexpression of Myc-T58A induced similar G2/M arrest at 3, 6, 24 and 48 h post-treatment with DL78 as seen in control cells (Figs. 6E and S7J). Lastly, a coimmunoprecipitation assay was performed to assess whether T58-Myc phosphorylation affected DL78-mediated displacement of Myc from α-tubulin. Remarkably, DL78 treatment resulted in a similar disruption of the interaction between Myc and α-tubulin in both Myc-T58A and wild-type Myc cells (Figs. 6F and S7K). Thus, these data suggest that DL78 treatment displaces Myc from α-tubulin, leading to T58-Myc phosphorylation and its subsequent loss. Collectively, these data highlight the identification of a novel Myc modulator that induces cancer-specific mitotic catastrophe by targeting Myc’s interaction with α-tubulin rather than its transcriptional activity (Fig. 6G).

### DL78 selectively localizes to tumor and reduces tumor burden

**Fig. 7:**
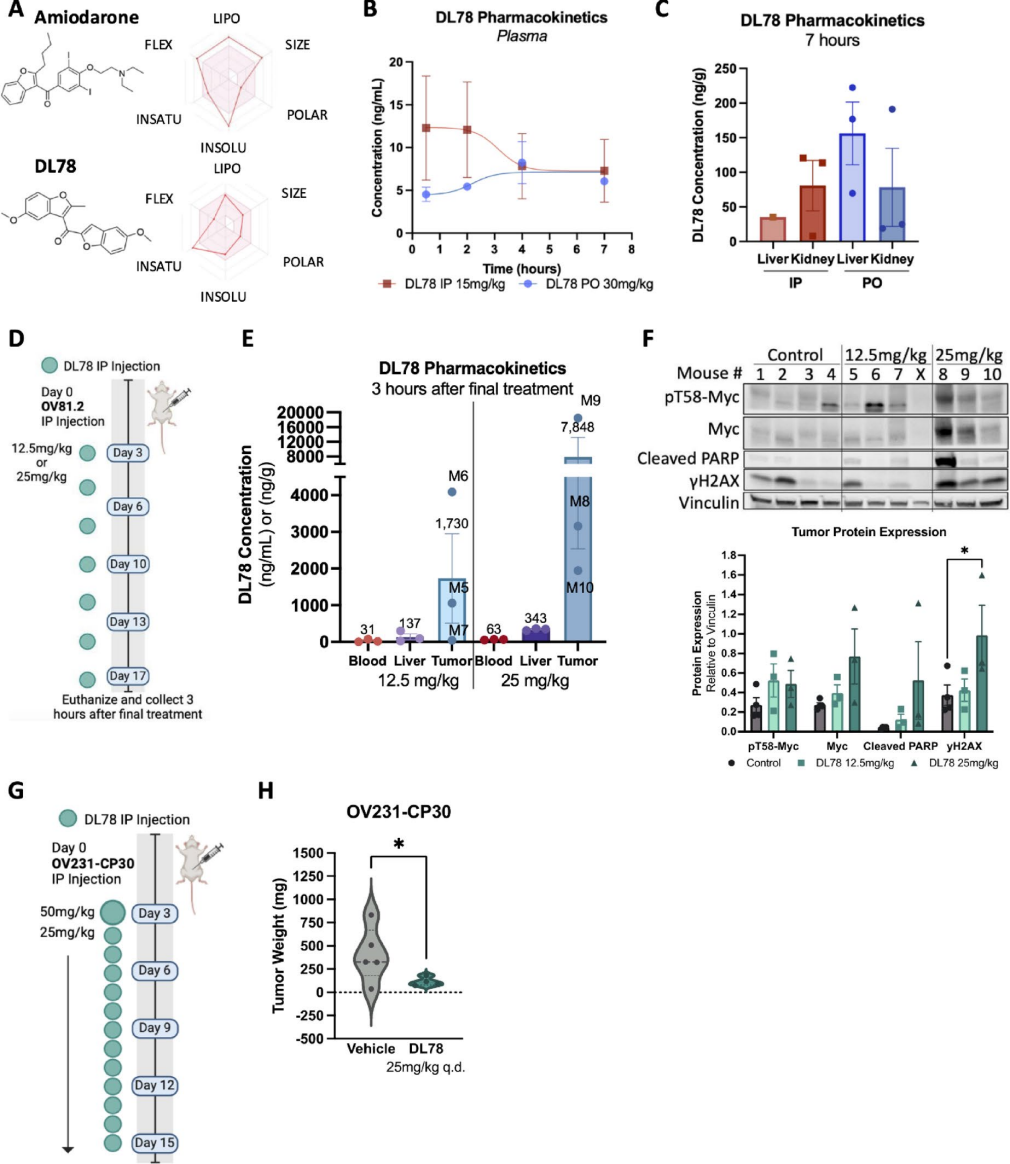
DL78 selectively accumulates in tumor 3 h after treatment and reduces tumor burden. (**A**) Screenshot from SwissADME in silico bioavailability analysis of amiodarone (top) and DL78 (bottom). The white hexagon represents various qualities of drug-likeness (lipophicity, polarity, solubility, saturation, flexibility). The shaded red hexagon represents the ideal score for a drug, and the red outline polygon represents the score of amiodarone or DL78. (**B**) Pharmacokinetics studies performed in healthy CD-1 mice. DL78 was given orally or intraperitoneally and the plasma concentration was measured over 7 h. (**C**) From the same pharmacokinetics study in (**B**), DL78 concentration was measured in kidney and liver tissue from both dosing methods 7 h after delivery. Two outliers were detected (removed from analysis) in the liver from the intraperitoneally dosed mice. (**D**) Design of pilot pharmacokinetic study in NSG mice injected intraperitoneally with OV81.2 cells. Three days later, treatment of DL78 began, dosed intraperitoneally at 12.5 mg/kg or 25 mg/kg and occurred about every other day. Schematic made with Biorender. (**E**) Pharmacokinetic analysis of DL78 in blood, liver, and tumor of treated mice 3 h after the final dose on Day 17. “M#” represents individual mouse. (**F**) Western blot from mouse tumors treated with vehicle, DL78 12.5 mg/kg or DL78 25 mg/kg. “X” removed from analysis. Quantification below. Significance calculated via one-way ANOVA, ∗*p* < 0.05. *n* = 3 mice. (**G**) Design of in vivo efficacy study in NSG mice injected intraperitoneally with OV231-CP30. Three days after injection, mice received 50 mg/kg IP loading dose, then daily IP injections at 25 mg/kg. Schematic designed in Biorender. (**H**) Total weight of tumor collected individually from each vehicle (*n* = 5) and DL78 (*n* = 4) treated mouse. ∗*p* < 0.05 as determined by unpaired t-test.

Upon ascertaining DL78’s mechanism of action, we were interested in characterizing its drug-like properties. Relative to the parent compound amiodarone, DL78 is predicted to have an improved drug-likeness, and slightly better solubility, based on SwissADME in silico bioavailability analysis (Fig. 7A)^65^. DL78 specifically improved in size, lipophilicity (more lipophilic is expected to have better absorption), flexibility (fewer than 9 rotatable bonds), and solubility, however, has increased saturation as a result of a lower ratio of sp3 hybridized carbons over the total carbon count. The properties that contribute to these characterizations are shown in Fig. S8A,B. Therefore, prior to conducting in vivo efficacy studies, the pharmacokinetics of DL78 were evaluated in healthy CD-1 mice over 7 h at 15 mg/kg administered intraperitoneally, or 30 mg/kg administered orally. DL78 had a low circulating concentration, with both intraperitoneal (12 ng/mL max) and oral (8 ng/mL max) dosing (Fig. 7B). However, in tissues, DL78 concentrations in kidney and liver of healthy mice in either dosing method were 6–20 fold higher, around 40–150 ng/g (Fig. 7C). These pharmacokinetic results are likely due to instability of the molecule because of the methoxy group, leading to quick metabolism in the liver, or poor absorption. Despite these challenges, we performed an in vivo orthotopic pilot study to determine pharmacodynamic differences in tumor-bearing mice. A pre-clinical metastatic ovarian cancer model of intraperitoneally (IP) injected patient-derived OV81.2 cells was used. Given that DL78 concentrations were slightly higher in plasma when mice were treated via IP injection, DL78 was delivered IP in vivo. We treated mice with either 12.5 mg/kg or 25 mg/kg DL78 every other day for 17 days (Fig. 7D). Pharmacokinetic analysis performed 3 h after the final dose showed that DL78 is found in low concentrations in the blood and liver, but nearly ten times higher in the tumor (Fig. 7E). Furthermore, protein isolated from the tumors showed trending increased T58-Myc phosphorylation, cleaved PARP, and a significant increase in γH2AX in mice treated with 25 mg/kg (Fig. 7F). Next, to investigate DL78’s potential effects on efficacy in ovarian cancer, we utilized a Myc-driven, metastatic platinum-resistant model of intraperitoneally injected OV231-CP30 cells. On Day 3, mice received a loading dose of DL78 at 50 mg/kg IP followed by 25 mg/kg IP daily treatment for 15 days (Fig. 7G). The mice did not show any overt signs of toxicity following DL78 treatment denoted by no change in weight compared to the vehicle treated mice (Fig. S8C). Daily administration of DL78 significantly decreased overall tumor burden and metastatic spread (Fig. 7H). These studies demonstrate the potential in vivo impact of DL78 as a cancer-specific agent and provide preliminary data to support further chemical optimization to improve its pharmacokinetics so that its translational impact can be fully evaluated.

## Discussion

Myc is amplified in over 30 human cancers, with HGSC having the highest rate of nearly 50%^21^. In the last two decades, disrupting this “undruggable”, highly relevant transcription factor garnered success through the development of Omomyc, Myc-Max dimerization repressors, and indirectly through BET inhibitors^21,61,66–75^. However, these approaches target Myc’s transcriptional activity and have yet to gain approval for clinical use. In this study, a novel Myc modulator, DL78, was characterized. Through the NCI-60 Screen, Micro-Tag technology, fluorescence polarization competition assay, and various molecular biology approaches, we uncovered that this small molecule directly binds to Myc, leading to selective acceleration of cancer cells into uncoordinated mitotic entry due to the disruption of Myc at the microtubules, and ultimately cumulating in mitotic catastrophe. Instead of inhibiting Myc’s transcriptional activity like most inhibitors, DL78 acts as a Myc modulator, utilizing Myc’s interaction with α-tubulin and its role in regulating mitotic fate to selectively target and eliminate cancer cells with high Myc expression.

### Pan-cancer activity and specificity

Through the NCI-60 Screen, DL78 demonstrated growth inhibition across 9 cancer types, particularly in highly proliferative, chromosomally unstable cells with high Myc expression. Interestingly, nonmalignant cells can escape the anti-mitotic effects of DL78, continue with normal but slowed division, and remain viable. There are several possible explanations for this cancer-selective phenomenon: (i) nonmalignant cells have the ability to regulate Myc expression and respond to elevated levels of Myc^76^; (ii) the cells lack cancer-driving properties, such as unregulated proliferation, oncogenic activation of Myc, and most importantly, chromosomal instability^77,78^; and finally, (iii) an additional checkpoint exists at the end of G2, prior to mitotic commitment, that is independent of the DNA Damage Checkpoint. This lesser discussed checkpoint, the antephase checkpoint, is utilized to prevent chromosome condensation and mitotic entry in response to chromosomal or microtubule poisons^79^. This checkpoint can be activated by CHFR, which is typically lost in transformed cells due to promoter methylation^80^. It is possible that the FT237 cells activate the antephase checkpoint to pause in G2, whereas it may be absent in the cancer cells, leading to a problematic mitotic entry. Furthermore, nonmalignant cells are known to be less sensitive to mitotic catastrophe than cancer cells^81^, which may be in part due to the lack of chromosomal instability.

While it has been shown that Myc can promote mitotic abnormalities and induce chromosomal instability in malignant and *TP53*-WT nontransformed RPE-1 cells, it was not sufficient in *TP53*-mutant fallopian tube FNE1 cells^25,26,64^. Similarly, FT237 + Myc did not show polyploidy populations after *MYC* overexpression. As a result, upon treatment with DL78, the FT237 + Myc cells arrested in G2-M at 6 h but quickly died rather than experiencing prolonged arrest. These results suggest that in addition to high levels of Myc, cells must have high levels of chromosomal instability at baseline for DL78 to induce mitotic catastrophe. This further highlights the specificity of DL78 towards cancer cells.

#### Leveraging Myc-induced mitotic abnormalities to promote mitotic catastrophe and disrupt Myc:α-tubulin interaction

Myc leads a fine line between promoting proliferation or apoptosis, which is controlled through its transcriptional activity^24,76^. Additionally Myc’s pivotal role in gene regulation orchestrates the expression of various mitotic genes^26^. Consequently, overexpression of *MYC* in malignant cells is known to induce chromosomal instability, sensitize cells to mitotic poisons, alter mitotic timing, and induce mitotic abnormalities, including altered spindle morphology^24–26^. Other groups have harnessed the *MYC* overexpression found in most cancers to induce synthetic dosage lethality with various cell cycle inhibitors, such as CDK1, Survivin, and the Aurora kinases^27–30,82–85^. Importantly, these inhibitors rely on Myc activity to push the cells towards Myc-driven apoptosis. Interestingly, DL78 induces a robust upregulation of Aurora A, both at the gene and protein level. AURKA is a known Myc target gene, and the two proteins have been shown to interact, stabilizing one another^82,84,86^. We propose that DL78 maintains Myc transcriptional activation and accelerates mitotic entry, resulting in the persistent upregulation of Aurora A, which normally peaks during M phase and is degraded upon mitotic exit. In the presence of DL78, however, Aurora A remains highly expressed due to the cells’ failure to complete mitosis. This suggests that DL78 not only sustains Myc’s role in driving mitotic abnormalities but also enables Aurora A to exacerbate these effects by promoting premature mitotic entry. Concurrently, DL78 disrupts Myc’s interaction with α-tubulin, impairing cytokinesis and leading to mitotic catastrophe. Thus, we describe an innovative approach of engaging Myc to induce mitotic stress specifically in cancer cells. By maintaining Myc’s transcriptional activity, DL78 can leverage the Myc-induced mitotic abnormalities, which result in cell death and Myc loss.

Myc is known to promote the G1-S transition, and even accelerate G1 exit in transformed cells or in response to stress^87–90^. Furthermore, it is hypothesized that Myc has an indirect role in driving inappropriate anaphase onset^83^. With DL78 treatment, a significant decrease in G1 is observed as cells quickly accumulate in G2-M. Interestingly, DL78’s efficacy correlated with increased *CCNE1* expression, which is found in Myc-driven cells^37,91^. As Cyclin E1 is essential for G1-S progression^92^, the presence of this protein may aid in propelling the cells out of G1 towards mitotic entry, exasperating DL78’s effects.

In the G2-M arrested cells, DL78 treatment led to improper spindle assembly, multiple aster formation, and decreased α-tubulin, but did not directly prevent α-tubulin polymerization in a cell-free pelleting assay. Typically, once the cells reach mitosis, Myc interacts with α-tubulin, protecting Myc from degradation^58^. We show that DL78 treatment significantly disrupted binding to α-tubulin. Unbound Myc is likely less stable, leading to an accumulation of T58-phosphrylation. This process may contribute to the eventual proteasomal degradation and loss of total Myc protein, consistent with previous findings that Vincristine decreases Myc levels following its dissociation from microtubules^58^. Importantly, this Myc depletion is specific to DL78 and is not a general consequence of mitotic arrest, as other G2-M arresting drugs like Paclitaxel and Nocodazole do not alter Myc protein^58,82^. Furthermore, this disruption could prevent transfer of Myc to daughter cells, which may affect the cancer cell’s viability or metastatic potential. While previous literature has described the Myc-α-tubulin interaction to regulate Myc’s stability or compartmentalization, the abnormal microtubule phenotypes observed with DL78 treatment could instead suggest the opposite relationship of Myc affecting microtubule function. This possibility was first described with LSF, a transcription factor, which acts with SET8 to enhance methylation on α-tubulin, affecting tubulin function^93^. Additional research is necessary to further elucidate the effects of Myc on α-tubulin.

### Improving compound solubility for additional preclinical study

While the discovery of this novel therapeutic approach is intriguing, the molecule has limitations that may hinder its clinical translation. Modifying DL78’s chemical structure to improve absorption, permeability, and solubility could result in a more potent compound. While high concentrations of DL78 were found in the tumor 3 h after treatment, there are limitations. First, the accumulation of DL78 in mouse tumors does not necessarily predict its behavior in patients and may underestimate potential toxicity. Second, these pilot studies were performed with a small number of mice (*n* = 3–5). Third, it is unknown how long DL78 remains in the tumor following treatment. Finally, although DL78 induces apoptosis after prolonged exposure, its greatest phenotype is growth inhibition. This fact, combined with the low concentration of circulating DL78 reported in healthy mice, may result in a shortened exposure of the tumor to the drug, allowing for continued tumor growth once the drug is metabolized. Other Myc interacting compounds, 10058-FA and 10074-G5, were shown to be quickly metabolized, preventing anti-tumor activity^72,73^. Given DL78’s observed effects on reducing tumor weight and Myc-leveraging properties, it is a promising chemical backbone to be modified for improved solubility, stability, and target engagement.

Once DL78 undergoes preclinical development, there is potential for the improved compound to have profound clinical implications. There are several connections between HGSC chemoresistance, Myc, and microtubules that support chemical modification leading to additional investigation of DL78. First, ovarian cancer patients who responded to platinum-based treatment had greater *MYC* expression than non-responders^94^ and patient-derived xenografts with higher *MYC* expression were more sensitive to anti-microtubule agents^58,95^. Additionally, platinum resistance in ovarian cancer has been shown to be dependent on the G2-M checkpoint due to dysfunction of the anaphase promoting complex and altered microtubule dynamics^39^. As a Myc modulator that creates abnormal mitotic spindles and kills cisplatin-resistant cells, DL78 could be successful in HGSC preclinical studies with an improved chemical structure. Finally, there are unfortunately many side effects with platinum, including nephrotoxicity, ototoxicity, neurotoxicity, cardiotoxicity, and hematological toxicity^96,97^. As a cancer-specific antimitotic agent with no observed toxicity in mice, DL78 presents a promising chemical scaffold for further optimization.

Overall, DL78 leverages Myc’s oncogenic abilities to accelerate cell cycle progression to uncoordinated mitotic entry. Concurrently, the G2-M arrested cells experience a decreased interaction between Myc and α-tubulin, resulting in increased phosphorylation of T58-Myc, and remain in mitosis until they endure mitotic catastrophe. While this study characterized DL78 in ovarian cancer, its broad pan-cancer activity allows for investigation in several other cancers, particularly leukemia, melanoma, and central nervous system, which appeared to be more sensitive to DL78 than non-small cell lung cancer, kidney, and prostate. Although the chemical structure presents challenges in pharmacokinetics, we have demonstrated that DL78 effectively infiltrates tumors and significantly reduces tumor burden without overt toxicity. This highlights its potential as a promising scaffold for further optimization to identify a structurally improved, more drug-like candidate. With further study, DL78 can become a potent compound to study biological functions in nonmalignant cells, such as G1 exit and the antephase checkpoint, as well as in cancer cells such as in the context of Myc-driven mitotic abnormalities, given the phenotypes DL78 induces. Furthermore, there is more to be studied in regard to DL78’s novel mechanism decreasing binding between α-tubulin and Myc and altering α-tubulin morphology, thus introducing a unique therapeutic window. Ultimately, our research demonstrates a small molecule is capable of leveraging cancer vulnerabilities of chromosomal instability and high Myc expression to selectively induce cell death.

## Materials and methods

### Compounds

Screened compounds were purchased from Molport. See Table S2 for list of compound names/IDs. Amiodarone (#S1979), Nocodazole (#S2775), MYCi361 (#S8905), and 10,074-G5 (#S8426) were purchased from Selleck Chemicals. Cisplatin was purchased from Fisher Scientific (#50-187-3570). SCH772984 was kindly provided by the Narla Laboratory. Cmpd 238 (MolPort) was identified in the screen and utilized as a structurally similar negative control. Receipt of the lead compound, DL78 ((5-methoxy-2-methylbenzofuran-3-yl)(5-methoxybenzofuran-2-yl)methanone), was confirmed through proton nuclear magnetic resonance, shown in Supplementary File 2. DL78 was later synthesized by the Vahlteich Medicinal Chemistry Core. To a solution of 2-hydroxy-5-methoxybenzaldehyde (1.08 g, 7.06 mmol) in acetone (20 mL) K_2_CO_3_ (1.95 g, 14.1 mmol) and 2-bromo-1-(5-methoxy-2-methylbenzofuran-3-yl)ethan-1-one (2.00 g, 7.06 mmol) were added. The reaction mixture was refluxed for 4 h. Then acetone was evaporated under reduced pressure. The residue was treated with dichloromethane and water. The organic phase was washed by brine, dried over anhydrous Na_2_SO_4_, and evaporated to give a brown gum. It was purified by column chromatography and then recrystallized from ethyl acetate/diethyl ether to give the title compound as solid (1.27 g, yield 53.4%). 1 H NMR (500 MHz, cdcl_3_) δ 7.53–7.47 (m, 2 H), 7.37 (d, J = 9.0 Hz, 1 H), 7.19 (d, J = 2.6 Hz, 1 H), 7.15–7.09 (m, 2 H), 6.94–6.87 (m, 1 H), 3.87 (d, J = 0.8 Hz, 3 H), 3.78 (d, J = 0.8 Hz, 3 H), 2.64 (d, J = 0.8 Hz, 3 H). MS: m/z 337.1072 [M + H] +. NMR from synthesis shown in Supplementary File 3. Of note, a majority of experiments are performed with 10µM dosing due the observed IC50 in a short-term viability assay.

### Cell culture

Cells were cultured in 10–15 cm plates at 37 °C in a humidified atmosphere (5% CO_2_). All cells were grown in filtered media supplemented with 10% FBS and 1% Pennicilin/Steptomycin. Specific media differed between cell lines (FT237, FT246: DMEM-F12; OV81.2, OV81.2-CP40, OV231, OV231-CP30, HeyA8: DMEM; OVCAR3, OVCAR8, Kuramochi: RPMI-1640). Cells were passaged at 80% confluency using trypsin (0.25%)/EDTA solution. All cell lines were authenticated via STR profiling and confirmed negative for mycoplasma testing, completed quarterly. FT237, FT246, and Kuramochi cells were gifts from the Drapkin Laboratory. FT237 + Myc was generated in the DiFeo Laboratory through lentiviral transduction of FT237 using the pLV-[MYC] vector with GFP selection marker. Generation and selection previously described^98^. OV81.2, OV81.2-CP40, OV231, OV231-CP30 were generated from patient samples in the DiFeo Laboratory^39,40^. OVCAR3 was gifted by the Zhang Laboratory. OVCAR8 was gifted by the McLean Laboratory. HeyA8 and CaOV3 were bought from AATC. A549 and NCI-H1975 were gifted by the Narla Laboratory. All methods were carried out in accordance with relevant guidelines and regulations, approved by Institutional Biosafety Committee (IBCA00001196) and the Institutional Review Board (PI: DiFeo) for human sample collection and use, and informed consent was provided for tumor donation.

### High-throughput screen

OV81.2 cells were plated at 125 cells/well in 100 μl of complete growth medium into white clear bottom 96 well plates, (Corning 3765). Test compounds were plated into the medium using TTP Labtech Mosquito instrumentation at 10, 3, and 1 µM final drug concentrations via dilution from a 2 mM stock source plate library. Replicate wells were plated in different locations for independent experiments. Positive control compound amiodarone was plated at drug testing concentrations onto each plate. Negative control wells comprised of DMSO were also included. DMSO concentration was normalized to 0.5% where needed across the entire plate. Plates were centrifuged (1 min, 1000 rpm) before incubation for 48 h at 37 °C with 5% CO_2_. Cell viability was determined using CellTiter-Glo (Promega #G7572). The general kit protocol was followed where 50ul of detection reagent was added to each well. Bulk reagent addition was performed by using a Multidrop Combi Reagent Dispenser (Thermo Scientific). Each plate was then mixed for 5 min prior to endpoint read. Luminescent intensity was detected using a PHERAstar plate reader (BMG Labtech) using a LUM plus optics modules. Reported values were normalized to DMSO-treated cells.

### Optical mapping of voltage changes

Commercially available cryopreserved hiPSC-CMs (iCell2, FujiFilm CDI, USA) were thawed according to manufacturer’s recommendation, plated in a 96 wells plate with matrigel (Corning, USA) coated PDMS inserts to induce maturation as previously described^99,100^ and cultured for 7 days prior to optical mapping of voltage changes to assess the effects of amiodarone (0.1, 5, 10, 20 µM), DL 78 (0.1, 5, 10, 20 µM), or E-4031 (500 nM). hiPSC-CMs were incubated with Fluovolt (1:1000, Thermo, USA) for 30 min and washed 2 times with complete HBSS prior to a 1-hour drug treatment in HBSS. Optical mapping movies were recorded at 200 frames per second and movies were analyzed with Scrow^99–101^ for obtaining frequency of spontaneous activation and action potential duration.

### Colony formation

Cells were plated in triplicate in low density (~ 100–200 cells/well) in a multi-well plate and left to settle overnight. Treatment began the following day and every 3 days the media was replaced with fresh media and newly dissolved drug. Once colonies were visible to the naked eye (around day 7), media was removed and colonies were fixed with 10% Acetic Acid 10% Methanol in water for 1 h. After removing the fixing solution, crystal violet dissolved in 100% methanol was added for at least 1 h or overnight. Crystal violet was removed and the plates were washed in tap water. Plates were tented to dry out for several hours or overnight prior to imaging on the BioRad ChemiDoc. Automated analysis was performed using a plug-in on Fiji.

### MTT assay

Cells were plated in triplicate in a 96-well plate and settled overnight. The following day, a serial dilution of the compound was prepared, and cells were treated at 70% confluency. After 48 h of treatment, MTT (Thermo Fisher Scientific M6494) was added to the media at 10% of the volume and incubated for 2 h at 37 °C. Media was removed and the cells were lysed with isopropanol. Absorbance was read at 600 nm on the Cytation 5 Bioteck Plate Reader.

### NCI-60 human tumor cell lines screen

DL78 was submitted to the National Cancer Institute following an accepted application to test the compound in their NCI-60 one-dose classic screen (96-well format, retired January 2024). Methods are described here: https://dtp.cancer.gov/discovery_development/nci-60/methodology.htm. Percent Growth data of the NCI-60 Screen was calculated as growth relative to the no-drug control, and relative to the time zero number of cells. Correlation data of DL78 compared to other microtubule inhibitors were obtained through the COMPARE database (https://dtp.cancer.gov/databases_tools/compare.htm). Raw sequencing data for the NCI-60 Human Tumor Cell Lines Screen was downloaded from Cell Miner^104^ and mapped to GRCh38 using STAR v2.5.2a^105^ and gene quantifications were calculated using Stringtie v2.1.1^106–108^ to quantify refGene annotations. Gene read counts were calculated using featureCounts^109^ v1.6.1 (subread).

### Annexin V flow cytometry

Cells were plated and settled overnight. At 70% confluency, cells were treated with 10 µM DL78 and harvested at 24 and 48 h. Media, PBS washes, and trypsinized cells were all collected in a 15 mL conical and spun down at 300 g at 4 °C for 5 min. The supernatant was aspirated, and the cell pellet was resuspended in PBS. Following a second spin down at 300 g at 4 °C for 5 min and aspiration of the supernatant, the cells were resuspended in 1× Annexin V Binding Buffer with Annexin V, per the kit’s protocol (Fisher Scientific #V13242). Cells were incubated in the dark at room temperature for 15 min. Diluted PI from the kit was then added to the suspended cells and they incubated for another 15–30 min prior to being analyzed on the BioRad Ze5. Data were analyzed in FlowJo.

### Protein extraction (cells)

Cells were plated in a 10 cm dish and settled overnight to reach 70% confluency at time of treating. Compound treatment was diluted in the media and added to the plates. At time of harvest, media, PBS wash, and trypsinized cells were collected into a 15mL conical. Cells were spun down at 300 g at 4 °C for 5 min. The supernatant was removed, and the cells were resuspended in PBS. After a second spin at 300 g at 4 °C for 5 min, the supernatant was aspirated, and the cell pellet was stored in − 80 °C until further processing. To lyse the cells, chilled RIPA (Thermo Fisher #89901) with protease and phosphatase inhibitors (Thermo Fisher #A32955, #A32957) was added directly to the frozen pellets. After resuspension, the lysate was kept on ice for 5 min, then vortexed and sonicated for ~ 45 s twice. Lysate was then spun down for at 15,000 rpm for 15 min. The supernatant was collected in a fresh Eppendorf tube and quantified via Pierce BCA Protein Assay (Thermo Fisher #23227). The plate was read on the Cytation 5 Bioteck Plate Reader at an absorbance of 562 nm.

### Protein extraction (animal tissue)

The tissue was stored in − 80 °C until processing. Chilled T-PER (Thermo Scientific #78510) with protease and phosphatase inhibitors (Thermo Fisher #A32955, #A32957) was added at 10 mL/1 g to lyse 60–80 mg of frozen tissue per sample. A mortar and pestle were used to break up the tissue. The sample was then vortexed and sonicated for 45 s. Lysate was centrifuged at 10,000 g for 7 min. The supernatant was collected and transferred to a new tube. Samples were quantified via Pierce BCA Protein Assay (Thermo Fisher #23227). The readout was completed on Cytation 5 Bioteck Plate Reader at an absorbance of 600 nm to reduce any present hemoglobin absorbance by a factor of 10, compared to reading at a 562 nm absorbance.

### Western blotting

Following quantification of the lysate, samples were diluted in the lysis buffer and NuPAGE LDS Sample Buffer (Thermo Fisher #NP0007) with 5% 2-Mercaptoethanol (Sigma-Aldrich, #M6250) to achieve desired protein concentration. Typically, 15–30 µg of protein were loaded for each sample. Samples were heated to 100 °C for 5 min then briefly centrifuged at 1200 rpm for 1 min prior to loading into a 12% Mini-PROTEAN gel (BioRad #4568046, #4568043, #4568045) in the Mini-PROTEAN Tetra Vertical Electrophoresis Cell (BioRad #1658004). PageRuler Prestained Protein Ladder was included in each gel (Thermo Scientific #26617). The finished gel was transferred on Trans-Blot Turbo Mini Nitrocellulose Transfer Packs (BioRad, #1704158, #1704159) on the Trans-Blot Turbo Machine. After blocking for 1 h in 5% milk (Fisher Scientific #50-488-785), the blot was cut for the specific proteins of interest and the primary antibody in 5% milk was added overnight. See Table S3 for the list of antibodies and their supplier. Following four 5 min washes of 1× TBST, the secondary antibody in 5% milk was added (Cell Signaling #7076, #7074) for 1 h at room temperature. After another four 5 min 1× TBST washes, the membranes were imaged using Western ECL Substrate (BioRad #1705062, #1705062; Cytivia #RPN2235) on the BioRad ChemiDoc. To probe for total protein after the phosphorylated protein, Restore PLUS Stripping Buffer (Thermo Fisher #46430) was added to the membrane for 8 min, then washed 3 times with 1× TBST for 10 min each. Blots were analyzed via Fiji. All blots are cropped in order to fit in the figure. Full-size blots are included in supplementary.

### Cell cycle profiling

Cells were plated in a 10 cm dish and settled overnight. By time of treatment, the cells reached 70% confluency. Media was replaced with fresh media containing diluted drug. At each timepoint, media, PBS wash, and trypsinized cells were collected into a 15mL conical and spun down at 300 g at 4 °C for 5 min. The supernatant was removed and the cell pellet was washed with PBS. After a second 300 g at 4 °C 5 min spin-down, the PBS was removed and the cell pellet was resuspended in 750 µL PBS. Next, 1750 µL of 100% Molecular Grade Ethanol (Sigma-Aldrich #E7023) was added dropwise to the cell suspension while placed on a low-speed vortexer to reach a final concentration of 70% ethanol. Samples were stored at − 20 °C overnight or up to 3 days. To process for flow cytometry, the samples were spun down at 300 g at 4 °C for 4 min and the supernatant was aspirated. The pellet was washed in 1mL PBS and centrifuged at 300 g at 4 °C for 4 min. The PBS wash was removed, and the pellet was resuspended in 500 μL FxCycle PI/RNase Staining Solution (Thermo Scientific #F10797). Samples incubated in the dark at room temperature for 15–30 min prior to being analyzed on the BioRad Ze5. The data were analyzed using FlowJo.

### Cell synchronization

Cells were synchronized for immunofluorescence microscopy and cell cycle profiling using a double thymidine block. To ensure that cells would be 70% confluent at time of treatment, cells were plated to reach 40% confluency at the beginning of the synchronization. The following day after plating cells, the media was removed. Thymidine (Sigma-Aldrich #T1895) dissolved in water was added to fresh media at a final concentration of 2 mM. After 18 h, the media was removed, cells were washed with PBS, and fresh media was added for 6 h to release the cells. Then a second 2mM thymidine treatment was added for another 18 h. After the second block, the cells were washed with PBS and released in fresh media and a 0 h timepoint was collected. Cells were treated with drug at the release and ultimately processed for microscopy or cell cycle.

### EdU assay

Cells were treated with DMSO, DL78 (10 µM), Cisplatin (5 µM) or Nocodazole (0.25 µM) for 6 h. 45 min prior to harvest, cells were treated with 10 µM EdU from the Invitrogen Click-iT™ Plus EdU Alexa Fluor 488 Flow Cytometry kit (#C10632). Media and cells were collected and processed per the manufacturer’s Click-iT EdU protocol. After the final wash step, the supernatant was aspirated with the pellet was resuspended with FxCycle PI/RNase Staining Solution (Thermo Scientific #F10797). Samples incubated in the dark at room temperature for 15–30 min prior to being analyzed on the BioRad Ze5. The data were analyzed using FlowJo.

### Immunofluorescence microscopy

Coverglasses (Fisher Scientific #12-541-005) were coated with CellTak (Corning # 354240) according to the manufacture’s protocol. After the coverglass airdried, they were placed in a 12-well plate and cells were plated such that confluency would be ~ 70% at time of treatment. Number of cells plated depended on if they were going to be synchronized or not. The media was replaced with diluted drug at the stated concentration. At the timepoint, cells were fixed to the coverglass with ice-cold 100% methanol for 10 min on ice. The methanol was aspirated, and cells were rehydrated with PBS, followed by three 5 min PBS washes. Cells were blocked in antibody diluent (2% BSA 0.1% Triton-X in PBS) for 45 min at room temperature. Primary antibodies were added to fresh antibody diluent and incubated on the cells overnight at 4 °C in the dark (c-Myc Alexa Fluor 546, 1:500 (Santa Cruz #sc-40 AF546) or α-tubulin-488 Conjugate, 1:1000 (CellSignaling #8058)). Antibody was collected the next morning, and the cells were washed for 5 min in PBS 3 times in the dark. The final wash was aspirated, and the coverglass was mounted onto glass slides (Fisher Scientific #22-170-302) using one drop of Prolong Diamond Antifade Mountant with DAPI (Thermo Fisher #P36962). Slides were kept in the dark overnight. Images were captured on the Nikon CSU-W1 SoRa or Nikon Ti2 Widefield Microscope. Quantification of α-tubulin in dividing cells was performed in Fiji using Cell Cy5 Mean of 8–12 cells.

### Microtubule pelleting assay

1 mg/ml of bovine brain tubulin was incubated in BRB80 (80 mM PIPES, 1 mM MgCI_2_, 1 mM EGTA pH 6.8) with various concentrations of DL78, 10 µM Nocodazole, or 1% DMSO (vehicle control) at 37 °C for 30 min and centrifuged at 100,000 g for 30 min at 37 °C. Pellets and supernatants were loaded on gels and stained with Coomassie. The intensity of the bands at 50 kDa was quantified using Fiji (ImageJ).

### Differential scanning fluorimetry

Tubulin (Cytoskleton #T240-A) was dissolved in Tubulin Buffer (Cytoskeleton #BST01-001) to reach a concentration of 10 mg/mL. DL78 and Vincristine were prepared as 5mM stocks. All components were prepared to reach a final concentration of 0.8 mg/mL (tubulin), 60, 50, 25, 10, 5 µM (compounds), and 2.5× (SYPRO Orange Protein Gel Stain (ThermoFisher # S6650)) and loaded in quadruplicate in a 384 well plate (Applied Biosystems #4483285). DMSO (vehicle) concentration across all wells was constant. Using the QuantStudio 7 (Life Technologies), the samples were subjected to temperatures from 25 °C to 95 °C with a ramp of 0.03 °C/s. Data were analyzed with the Protein Thermal Shift Analysis software (Life Technologies) to fit to a Boltzmann function to determine T_m_. Raw fluorescent data were normalized for visualization.

### RNA extraction and qPCR

Media, PBS wash, and trypsinized cells were collected in a 15mL conical and spun down at 300 g at 4 °C for 5 min. The supernatant was aspirated, and the pellet was washed with PBS. The cells were spun down a second time at 300 g at 4 °C for 5 min and the PBS was aspirated. Pellets were stored at − 80 °C until processing. Total RNA was extracted using the RNeasy Plus Kit (Qiagen #74134) according to the manufacturer’s protocol. RNA was quantified on the NanoDrop and 1 µg was used for cDNA synthesis in conjunction with the High-Capacity RNA-to-cDNA Kit (Thermo Fisher #4387406) according to the manufacturer’s protocol. cDNA was diluted 1:24 in molecular grade water and loaded into a 384-well plate along with PowerTrack SYBR Green Master Mix for qPCR (Thermo Fisher #A46109) and DNA primers. Primers were designed using the sequence published on NCBI and Primer3a, then confirmed via UCSC in silico PCR. The plate was read on a QuantStudio 5 Real-Time PCR System (Thermo Fisher #A34322) and data was analyzed using the 2^–∆∆Ct^ method.

### Proteosome inhibitor (Bortezomib)

OV81.2 cells were treated with DMSO, 1µM Bortezomib (Selleck Chemicals, S1013), 10µM DL78, or combination of Bortezomib and DL78 for 3, 6, or 24 h. Media and cells were collected and processed as described for Western blotting.

### Cycloheximide assay

OV81.2 cells were pretreated with DMSO or 10µM DL78 for 12 h. The 0 min plate was harvested prior to Cycloheximide (CHX) (Sigma-Aldrich #C48590-1ML) addition. CHX was added to the pretreated cells at a final concentration of 75 µg/mL. Plates were collected after 5, 15, 30, 60, or 180 min and processed as described for Western blotting.

### c-Myc transcription factor activity assay (E-Box)

We utilized the RayBio Human c-Myc Transcription Factor Activity Assay (#TFEH-CMYC), which included a microplate with bound c-Myc E box DNA probes, along with specific and non-specific competitor DNA probes. Nuclear lysates were collected from DMSO or 10µM DL78 treated cells (3, 6, or 24 h) via the NE-PER™ Nuclear and Cytoplasmic Extraction Reagents (Thermo Scientific #78833) following the manufacturer’s protocol. The lysates were added to the microplate, along with the competitors, according to the manufacturer’s protocol. The plate was read out on Cytation 5 Bioteck Plate Reader at an absorbance of 450 nm.

### RNA sequencing and gene expression analysis

RNA was extracted using the RNeasy Plus Kit (Qiagen #74134) according to the manufacturer’s protocol. RNA sequencing was performed by the University of Michigan Advanced Genomics Core, with PolyA+, stranded libraries constructed and subsequently subjected to 151 paired-end cycles on the NovaSeq-6000 platform (Illumina). Initial data analysis was performed by the University of Michigan Bioinformatics Core. Raw reads were trimmed using Cutadapt (v2.3)^110^. FastQC (v0.11.8) was used to ensure the quality of data^111^. Fastq Screen v was used to screen for various types of contamination^112^. Reads were mapped to the reference genome GRCh38 (ENSEMBL) using STAR (v2.7.8a)^105^ and gene count estimates were assigned using RSEM (v1.3.3)^113^. Alignment options followed ENCODE standards for RNA-seq (https://github.com/alexdobin/STAR/blob/master/doc/STARmanual.pdf). FastQC was used in an additional post-alignment step to ensure that only high-quality data were used for expression quantitation and differential expression.

Differential gene expression across groups was analyzed with DESeq2 in R. The obtained differentially expressed protein-coding genes (DEGs, defined as fold change (FC) > 1.5 and FDR adjusted p-values < = 0.1) were used for gene set enrichment analysis with a web-based functional enrichment analysis tool called WebGestalt (WEB-based Gene SeT AnaLysis Toolkit) available at http://www.webgestalt.org/. Log2 fold change (with shrinkage estimator) values obtained from DESeq2 were also utilized to perform Gene Set Enrichment Analysis (GSEA) using https://www.gsea-msigdb.org/gsea/index.jsp.

### Cell target engagement assay

The cell target engagement assay was performed using CellarisBio’s Micro-Tag enzyme complementation method. Gapdh (UniProt ID: P04406), c-Myc (UniProt ID: P01106), and ERK2 (UniProt ID: P28482) Micro-Tag constructs were engineered by cloning the 15-amino acid Micro-Tag at the C-terminus. Constructs were transiently expressed in HEK293 cells using Lipofectamine3000. Two days post-transfection, cells were lifted, washed with TBS and non-denaturing lysates prepared by lysis with 1% Triton-X-100 in TBS for 1 h on ice. Lysates were clarified by full speed centrifugation at 14 K rpm for 1 min. A thermal profile was determined for each target by applying a thermal gradient for 3 min spanning a range from 44 °C to 68 °C to diluted lysates (1/20) followed by 3-min cool down at ambient room temperature. Micro-Tag binding protein and substrate were added and fluorescence signal detected. A plot of fluorescence with temperature identified a temperature of aggregation (T_agg_(50)) of 53 °C for c-Myc, 54 °C for ERK2, and 55 °C for Gapdh. Binding of inhibitors was tested at the T_agg_(50) temperature. Briefly, non-denaturing lysates from cells expressing the Micro-Tag construct were diluted 1/20 and aliquoted to wells of a PCR plate. Increasing doses of compound was added and incubated with cells for 30 min on ice followed by heating for 3 min at the T_agg_(50) temperature and a 3 min cool down at ambient room temperature. Micro-Tag binding protein and substrate were added followed by detection of fluorescence signal. Fluorescence signal was plotted with concentration of the compound on a semi-log scale using GraphPad Prism 9.0 software. Non-linear regression analysis was used to fit a Sigmoidal dose-response curve with variable slope to generate EC50 values for cell target engagement for the compounds.

### KINOMEscan™

DL78 and SCH772984 were tested in the ERK1 and ERK2 KINOME*scan*™ performed by Eurofins Discovery as previously described^114^.

### Fluorescence polarization

Compounds were diluted to a 5 mM stock and added to a 384-well plate (Corning 4514) in triplicate using the Echo 655 Liquid Handler at various concentrations (10074-G5 10 µM, MYCi361 25 µM, DL78 20, 40, and 60 µM, SCH772984 60 µM, and Cmpd 238 60 µM. Wells were supplemented with additional DMSO to ensure equal DMSO concentrations across all conditions. Full length c-Myc protein was kindly provided by Dr. Arul Chinnaiyan at the University of Michigan and stored in 50 mM Tris HCL pH 8.0, 300 mM NaCl, 5% Glycerol, and 1 mM DTT. Immediately after thawing on ice, 10 µM Myc was added to the plate with the compounds and incubated for 30 min at room temperature. The plate was then read out on PHERAstar (BMG Labtech) using an optic module of 470 nm excitation and 550 nm emission (BMG Labtech).

### siRNA transfections

Opti-MEM (Thermo Fisher Scientific #31985070), Lipofectamine3000 (Thermo Fisher Scientific #L3000015), and the specific siRNA (Fisher Scientific: negative control No. 1 siRNA #AM4635, Silencer siRNA Myc #3107, or Silencer siRNA Myc #106821) were combined and incubated as described by ThermoFisher. Immediately after plating the cells, the opti-MEM, lipofectamine, and siRNA combination was added dropwise to the cells, which incubated overnight. Treatments with compound began the following morning.

### Lentiviral transduction

The pLenti6.3/EV, pLenti6.3/V5-DEST-Myc pLenti6.3/V5-DEST-Myc-T58A lentivirus were generous gifts from the Narla Laboratory. Lentiviral transduction of OV81.2 and OV231 cells was completed as previously described^115^ and selected for over several passages with Blasticidin (Invivogen #ant-bl-05).

### Co-immunoprecipitation

Cells were plated in 15 cm dishes and treated at 10 µM for 6 h. Media, PBS wash, and trypsinized cells were collected into a 50 mL conical. Cells were spun down for 5 min at 300 g at 4 °C and the supernatant was aspirated. Cells were washed in PBS, placed in a pre-weighed tube, and spun down again at 300 g at 4 °C for 5 min. The PBS was aspirated, and the cell pellet was weighed. Chilled M-PER (Thermo Fisher #78501) with combined EDTA-free proteinase and phosphatase tablet (Thermo Fisher #A32961) as added to the pellets at a 1:8 ratio. Cells were resuspended and kept on ice for 5 min prior to being spun down at 2600 g at 4 °C for 5 min. The supernatant was collected into a new tube. Samples were quantified via Pierce BCA Protein Assay (Thermo Fisher #23227). The readout was completed on Cytation 5 Bioteck Plate Reader at an absorbance of 562 nm. 1 µg of protein per condition was loaded onto Dynabeads (Protein A–Thermo Fisher #10006D) bound with 1 µg IgG (Cell Signaling, #3900) for preclearing. Precleared samples were then incubated overnight with rotation with Dyanbeads bound with either 5 µg IgG or 5 µg V5 (Bethyl #A190-120 A). Washes were performed with PBS + 0.02% Tween20, except for the final wash with PBS alone. Samples were eluted according to the kit’s protocol and analyzed via western blotting. Conformation specific secondary was used for anti-rabbit primaries (Cell Signaling #5127).

### In silico ADME

SMILES of DL78 and Amiodarone were used as input for SwissADME^65^.

### Pharmacokinetics (PK)

Short PK in healthy mice: For intraperitoneal (IP) (15 mg/kg) and oral (PO) (30 mg/kg) dosing, DL78 was dissolved in PBS containing 20% DMSO and 40% PEG-400. At the given time points (0.5 h, 2 h, 4 h, 7 h), blood samples were collected from 3 female ICR (CD-1) outbred mice (Inotiv, ICR (CD-1); aged 8–16 weeks, weights varied from 26.2 g to 32.4 g) using heparinized calibrated pipettes with serial bleeding. Samples were centrifuged at 15,000 rpm for 10 min. Subsequently, plasma was collected from the upper layer. The plasma was frozen at − 80 °C for later analysis. All methods were carried out in accordance with the relevant guidelines and regulations, including the ARRIVE guidelines. The University of Michigan Institutional Animal Care and Use Committee approved this study.

PK on tumor-bearing mice: 10 female NSG mice (Jackson Labs, RRID: IMSR_JAX:005557; aged 8–12 weeks, weights varied from 22.7 g to 28.4 g) were injected intraperitoneally with 1 × 10^6^ OV81.2 cells on Day 0. Mice were randomly assigned to treatment groups and treatment began on Day 3 and occurred Mondays, Wednesday, and Fridays. DL78 was dissolved prior to each injection in 10% N,N-Dimethylacetamide, 10% Solutol, and 80% PBS and dosed at 12.5 mg/kg or 25 mg/kg. On Day 17, mice received the final treatment. Three hours after, mice were euthanized via CO_2_ administration in a small mouse cage using a compressed CO_2_ gas cylinder with a pressure-reducing regulator at a flow rate of 3 L/min for 5 min followed by cervical dislocation. Blood was immediately collected with heparin-coated needles via cardiac puncture. Tumor (0.375 g to 0.676 g) and liver were collected from each mouse and stored at − 80 °C until processing. All methods were carried out in accordance with the relevant guidelines and regulations, including the ARRIVE guidelines. The University of Michigan Institutional Animal Care and Use Committee approved this study. Blood, liver, and tumor samples were stored at − 80 °C until processing. Calibration standards (CSs) were thawed and allowed to equilibrate at room temperature. The tissue samples were mixed with Water/ACN (4:1) solution, then homogenized by Precellys Evolution homogenizer. To an aliquot of 40 µL of spiked plasma or tissue homogenized solution, 120 µL of cold ACN solution with internal standard (IS) was added and vortexed for 10 min. Clofazimine was used as the internal standard. The samples were then centrifuged at 15,000 rpm for 10 min at 10 °C. After centrifugation, the supernatant organic layer was transferred to HPLC 96-well sample plate, and 5 µL was used for injection in the chromatographic system.

LC-MS/MS: All samples were analyzed via LC-MS/MS. The analytical curve was constructed using ten non-zero standards with DL78 concentration ranging from 1 to 5000 ng/mL in the blank plasma, liver and kidney. A blank sample (matrix sample processed without internal standard) was used to exclude contamination. The linear regression analysis of DL78 was performed by plotting the peak area ratio (y) against DL78 concentration (x) in ng/mL. The linearity of the relationship between peak area ratio and concentration was demonstrated by the correlation coefficients (R) obtained for the linear regression of (r = > 0.990 in all samples).

### in vivo efficacy study

9 female NSG mice (Jackson Labs, RRID: IMSR_JAX:005557, aged 8–12 weeks, weights varied 18.2 g to 25.2 g) were injected intraperitoneally with 1 × 10^6^ OV231-CP30 cells on Day 0. Mice were randomly assigned to treatment groups and treatment began on Day 3 and occurred daily. DL78 was dissolved prior to each injection in 10% N, N-Dimethylacetamide, 10% Solutol, and 80% PBS and dosed at 12.5 mg/kg or 25 mg/kg. On Day 17, mice received the final treatment. Three hours after, mice were euthanized via CO_2_ administration in a small mouse cage using a compressed CO_2_ gas cylinder with a pressure-reducing regulator at a flow rate of 3 L/minute for 5 min followed by cervical dislocation. Blood was immediately collected with heparin-coated needles via cardiac puncture. Tumor (ranging from 74.9 mg to 831.7 mg) and liver were collected from each mouse and stored at − 80 °C until processing. All methods were carried out in accordance with the relevant guidelines and regulations, including the ARRIVE guidelines. The University of Michigan Institutional Animal Care and Use Committee approved this study.

### Statistical analysis and graphing

Analysis is described in each figure legend. Graphing and statistical analysis was performed in GraphPad Prism Version 10 (RRID: SCR_000306). Data plotted as mean ± standard deviation of the triplicate unless stated otherwise. Two group comparisons were analyzed via the two-tailed student *t* test. Multiple comparisons were analyzed via an ANOVA and Tukey multiple comparisons test.

## Supplementary Information

Below is the link to the electronic supplementary material.

Supplementary Material 1

## Data Availability

Gene and isoform level expression values produced by RSEM in counts, TPM, and FPKM, as well as the gene lists generated by DESeq2, are available at the GEO accession GSE267208 via https://www.ncbi.nlm.nih.gov/geo/query/acc.cgi?acc=GSE267208 . NCI-60 cell line data can be publicly accessed at https://discover.nci.nih.gov/cellminer/ along with the COMPARE database at https://dtp.cancer.gov/databases_tools/compare.htm. DL78 was tested in the NCI-60 Screen with the NSC identifier 848692. Data can be viewed with a DTP supplier account to the COMPARE database linked above (input NSC and check “Include one dose experiments”) or provided upon request. Other data generated in this study are available upon request from the corresponding author.
