# Supplementary material for: Pan-cancer Myc modulator that targets Myc-α-tubulin interaction to drive selective mitotic catastrophe
Source: Sci Rep. 2025 Oct 31;15:38188. doi: 10.1038/s41598-025-22011-4 (PMC12578881; doi:10.1038/s41598-025-22011-4)

SUPPLEMENTARY MATERIAL

Pan-Cancer Myc Modulator that Targets Myc-α-Tubulin Interaction to Drive Selective Mitotic Catastrophe in Cancer

Jessica Teitel, Ph.D.[1,2], Margaret Farah[2], Michele L. Dziubinski, M.S.[2], Pil Lee, Ph.D.[3], Andrew White, Ph.D.[3], Alexander Sobeck[2], Jose Colina, Ph.D.[1,2], John Takyi-Williams, Ph.D.[4], Bo Wen, Ph.D.[5], Elmar Nurmemmedov, Ph.D.[6], Ivan Babic, Ph.D.[6], Andre Monteiro Rocha, DVM, Ph.D.[7,8], Karan Bedi, M.S.[9], Aaron Robida, Ph.D.[10], Grace McIntyre[1,2], Takashi Hotta, Ph.D.[11], Yinzhi Lin, Ph.D.[1,2], Sreeja C. Sekhar, Ph.D.[1,2], Ryoma Ohi, Ph.D.[11], and Analisa DiFeo, Ph.D.[1,2,12*]

[1]Department of Pathology, University of Michigan, Ann Arbor, MI 48109, USA
[2]Rogel Cancer Center, University of Michigan, Ann Arbor, MI 48109, USA
[3]College of Pharmacy, University of Michigan, Ann Arbor, MI 48109, USA
[4]Therapeutic Systems Research Laboratories (TSRL), Inc., Ann Arbor, MI 48105, USA
[5]Pharmacokinetics Core, University of Michigan, Ann Arbor, MI 48109, USA
[6]CellarisBio, San Diego, CA 92121, USA
[7]Frankel Cardiovascular Center Cardiovascular Regeneration Core Laboratory, University of Michigan, Ann Arbor, MI 48109, USA
[8]Internal Medicine, Cardiology; University of Michigan, Ann Arbor, MI 48109, USA
[9]Department of Biostatistics, University of Michigan, Ann Arbor, MI 48109, USA
[10]Center for Chemical Genomics, University of Michigan, Ann Arbor, MI 48109, USA
[11]Department of Cell and Developmental Biology, University of Michigan, Ann Arbor, MI 48109, USA
[12]Lead contact
*Correspondence: adifeo@med.umich.edu

Figure S1. Viability and NCI-60 Screen data.

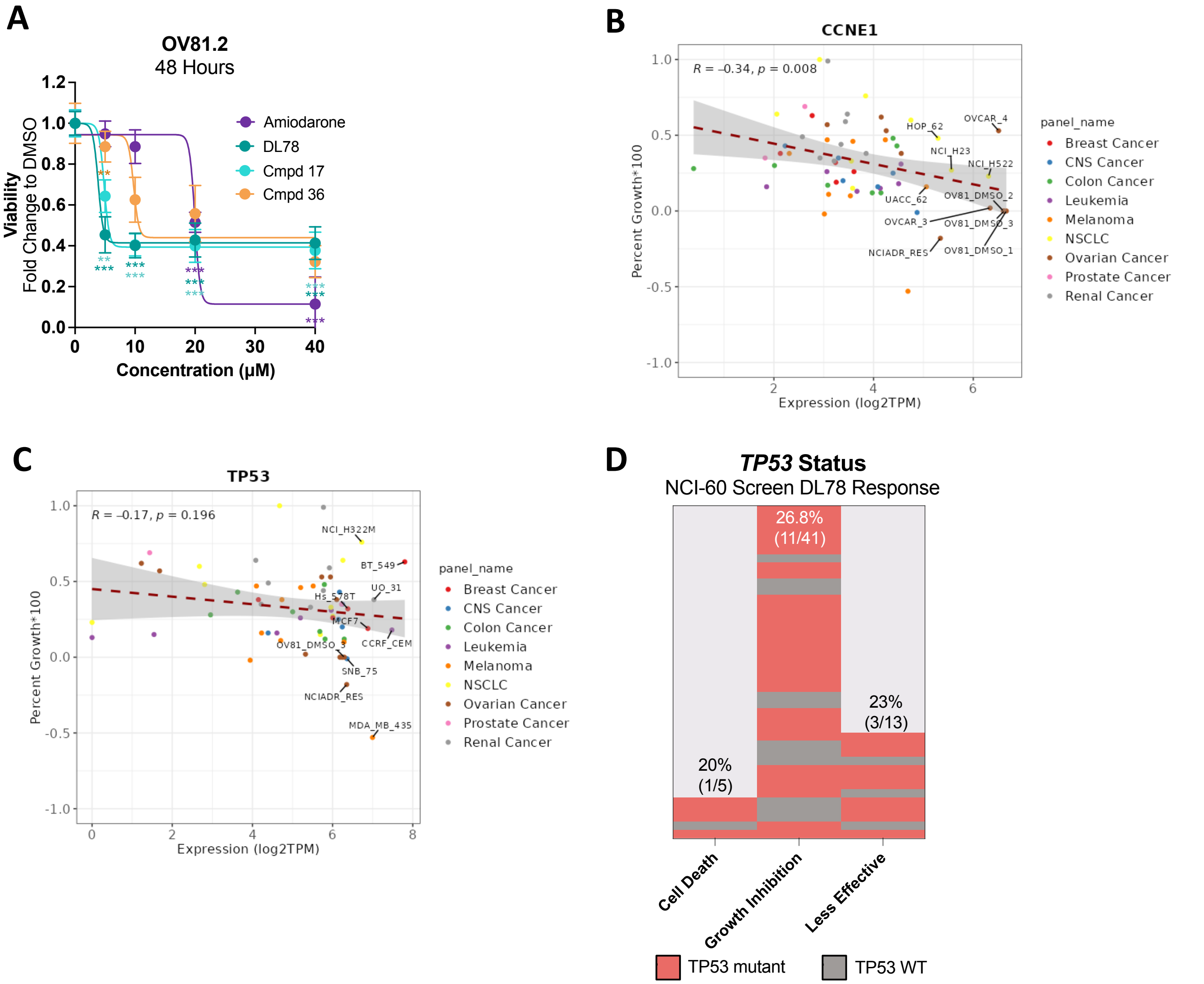

FIGURE S1. Viability and NCI-60 Screen data.
(A) MTT in OV81.2 cells after 48 hours of treatment with amiodarone, DL78, Cmpd 17, or Cmpd 36. Data plotted as mean with error bars as standard deviation, n=3 biological replicates.
$^{**}p < 0.01$ and $^{***}p < 0.001$ as determined by two-sided Student's t-test.
(B) Correlation of *CCNE1* expression or (C) *TP53* expression in the NCI-60 Screen with DL78 10μM treatment.
(D) *TP53* mutational status in cell lines from the NCI-60 Screen grouped by response to DL78 via percent growth: cell death ≤0%; growth inhibition ≤49.99%; less effective ≥50%.

Figure S2. Apoptosis flow cytometry graph.

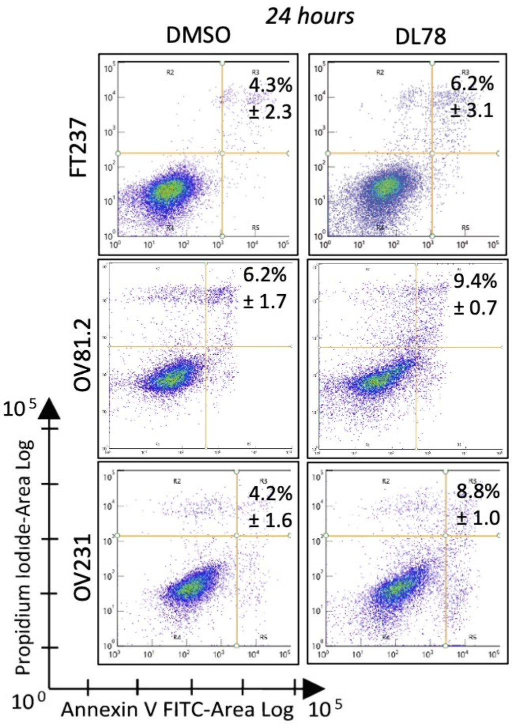

FIGURE S2. Apoptosis flow cytometry graph.
Representative flow plots of Annexin V/PI of FT237, OV81.2, and OV231 cells treated with 10μM DL78 for 24 hours. Number in the top right quadrant is the average total apoptosis, calculated via summation of the top and bottom right quadrants.

Figure S3. Cell cycle and western blot quantifications and microtubule pelleting assay.

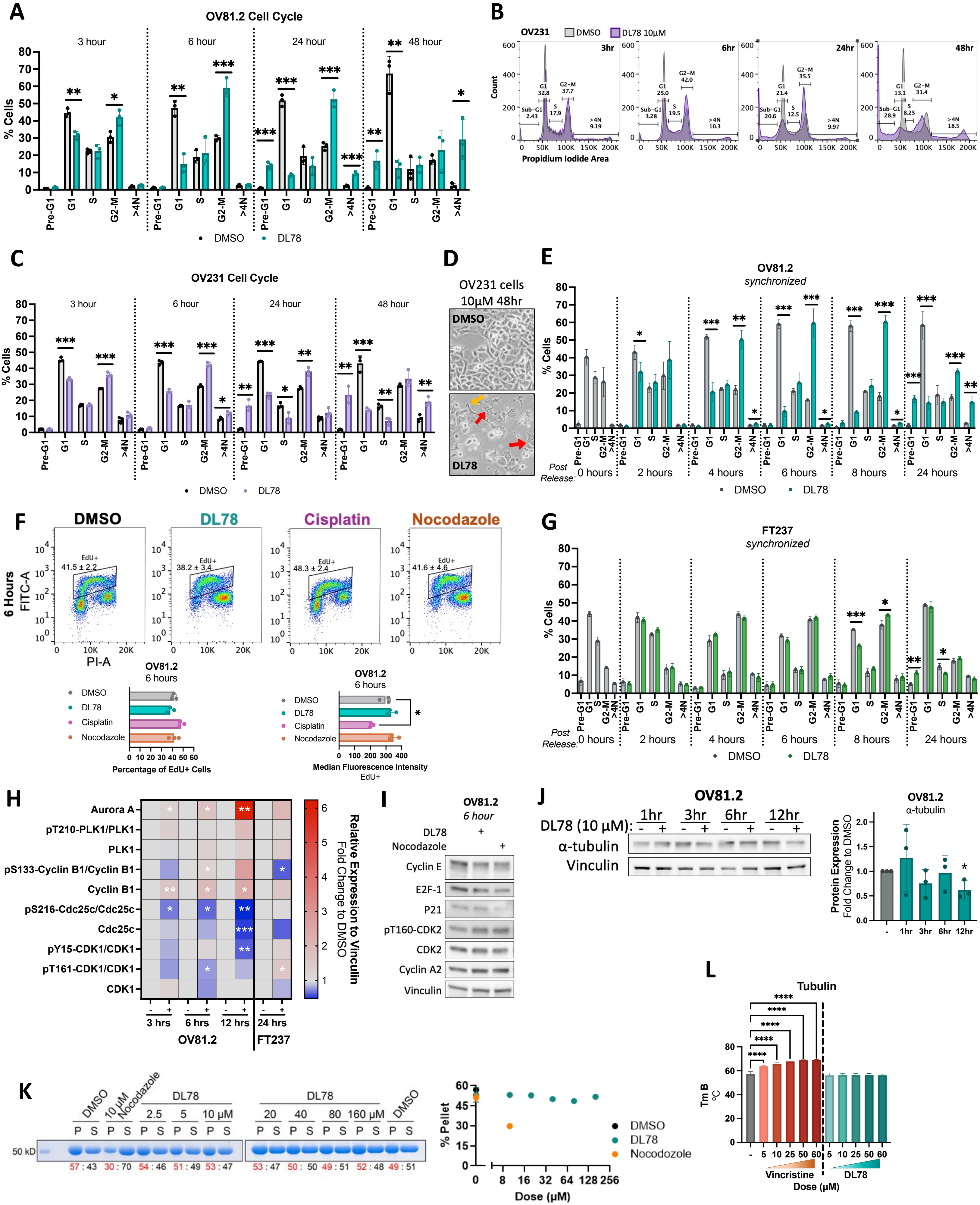

FIGURE S3. Cell cycle quantification, western blots, and microtubule pelleting assay.

(A) Quantification of OV81.2 cell cycle analysis in Figure 3A.

(B) Representative histograms of flow cytometry propidium iodide cell cycle analysis of OV231 cells treated with DMSO or 10μM DL78 over 3, 6, 24, or 48 hours. Statistics on graph are for DL78-treated cells.

(C) Quantification of OV231 cell cycle analysis in Figure S3B.

(D) Microscopy images at 10X magnification of OV231 following 48 hours of DMSO or 10μM DL78 treatment. Colored arrows in OV81.2 treated cells highlight enlarged, multinucleated cells (red arrow) and micronuclei (yellow arrow).

(E) Quantification of double-thymidine block synchronized OV81.2 cell cycle analysis in Figure 3C.

(F) Representative plots of EdU and PI analyzed via flow cytometry following 6 hours of compound treatment (DMSO, DL78 10μM, Cisplatin 5μM, Nocodazole 0.25μM). Quantifications below. On the left, average percentage of cells in EdU+ gating. On the right, quantification of EdU+ median fluorescence intensity.

(G) Quantification of double-thymidine block synchronized FT237 cell cycle analysis in Figure 3D.

(H) Quantification of Western blots in Figures 3E-3F.

(I) Western blot in OV81.2 cells treated with 10μM DL78 or 0.25μM Nocodazole for 6 hours.

(J) Western blot in OV81.2 cells treated with DL78 over a 12-hour timecourse. Quantification of Western blot below.

(K) Microtubule pelleting assay in the presence of DMSO, 10μM Nocodazole, or various concentrations of DL78. P=pellet; S=supernatant. Numbers below the gel are quantification; red values are quantification of the pelleted fraction, which is plotted below.

(L) Boltzmann T_m analysis from differential scanning fluorimetry of tubulin exposed to increasing concentrations of Vincristine or DL78 (5-60μM). Significance calculated via one-way ANOVA.

Data plotted as mean with error bars as standard deviation, n=3 biological replicates.

*$p < 0.05$, **$p < 0.01$, and ***$p < 0.001$ as determined by two-sided Student's t-test.

Figure S4. Western quantification and RNASeq.

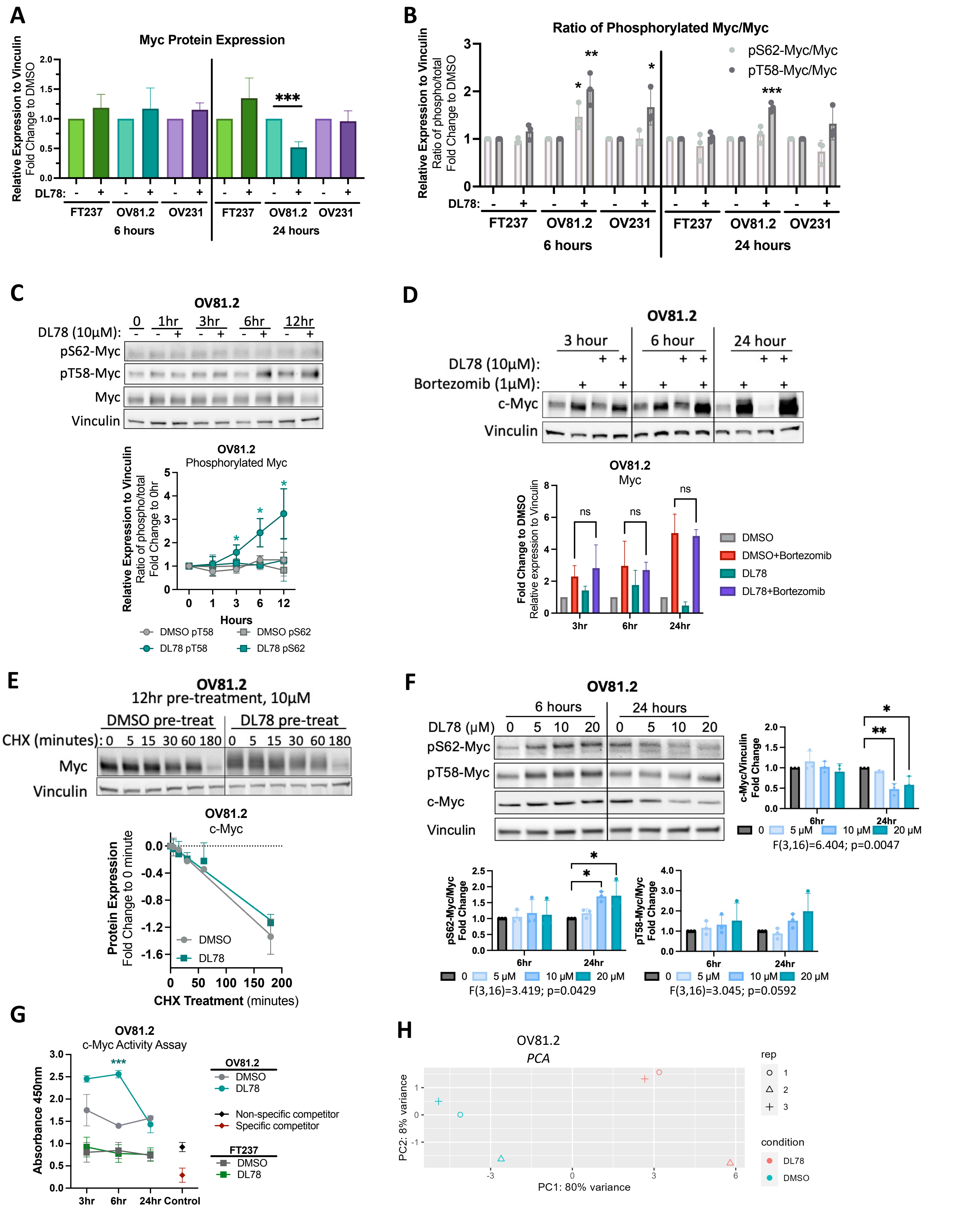

FIGURE S4. Western quantification and RNASeq.

(A) Quantification of Western blot in Figure 4A.

(B) Quantification of Western blot in Figure 4A, specifically the ratio of phosphorylated Myc to total Myc.

(C) Western blot with and without 10μM DL78 treatment over a 12 hour time course. Quantification below of the ratio of phosphorylated Myc to total Myc relative to DMSO at each timepoint.

(D) Western blot of Bortezomib treatment time course with or without 10μM DL78 in OV81.2. Quantification to the right.

(E) Western blot of OV81.2 cells pretreated with DMSO or DL78 10μM for 12 hours, followed by cycloheximide (CHX) treatment across several timepoints up to 3 hours. Quantification below.

(F) Western blot of OV81.2 cells treated with increasing concentrations of DL78 at 6 and 24 hours. Quantification of the the ratio of phosphorylated Myc to total Myc relative to DMSO at each timepoint. Two-way ANOVA stats below each graph.

(G) c-Myc Transcription Factor Activity Assay in OV81.2 and FT237 DMSO or 10μM DL78-treated lysates for 3, 6, or 24 hours.

(H) Principal component analysis plot of RNASeq data from OV81.2 treated with 10μM DL78 or DMSO.

Figure S5. MYCi361 and Micro-Tag data.

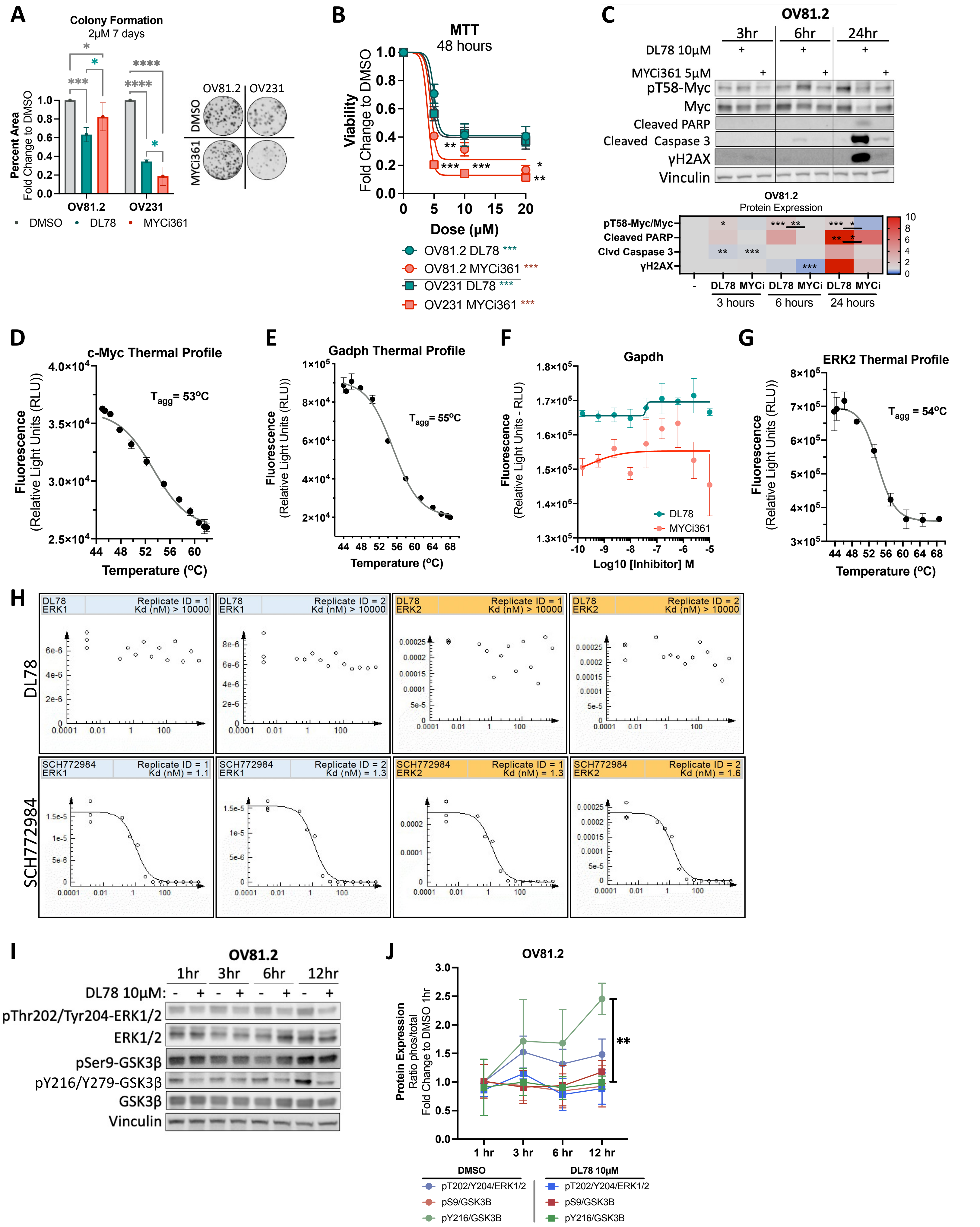

FIGURE S5. MYCi361 comparison and Micro-Tag data.

(A) Colony formation in OV81.2 and OV231 with DMSO, 2μM DL78, or 2μM MYCi361 treatment for 7 days. Significance determined by one-way ANOVA. Representative images to the right.

(B) MTT in OV81.2 (circles) and OV231 (squares) with DL78 2μM or MYCi361 2μM treatment for 48 hours. Colored asterisks next to key denote significance across all doses compared to 0μM. Black asterisks denote significant differences between DL78 and MYCi361 at the respective dose.

(C) Western blotting in OV81.2 treated with DMSO, 10μM DL78, or 5μM MYCi361 for 3, 6, or 24 hours. Quantification below.

(D) Florescent readout of the Myc-MicroTag in HEK293 cell lysate at the indicated temperatures to generate a thermal profile for Myc.

(E) Fluorescent readout of the Gapdh-MicroTag in HEK293 cell lysate at the indicated temperatures to generate a thermal profile for Gapdh.

(F) Fluorescent readout of the Gapdh-MicroTag in HEK293 cell lysate treated with DL78 or MYCi361 over a dose curve (10μM to 0.4nM) for 30 minutes.

(G) Fluorescent readout of the ERK2-Micro-Tag in HEK293 cell lysate at the indicated temperatures to generate a thermal profile for ERK2.

(H) KINOME*scan*™ by Eurofins Discovery of ERK1 (blue header) or ERK2 (yellow header) with DL78 (top row) or SCH772984 (bottom row) treatment.

(I) Western blot of OV81.2 treated with DMSO or 10μM DL78 over time.

(J) Quantification of Western blot in Figure S5H. Black asterisk denotes significant difference between DMSO and DL78 10μM.

Data plotted as mean with error bars as standard deviation, n=3 biological replicates.

*$p < 0.05$, **$p < 0.01$, and ***$p < 0.001$ as determined by two-sided Student's t-test.

Figure S6. Cell cycle and western quantification.

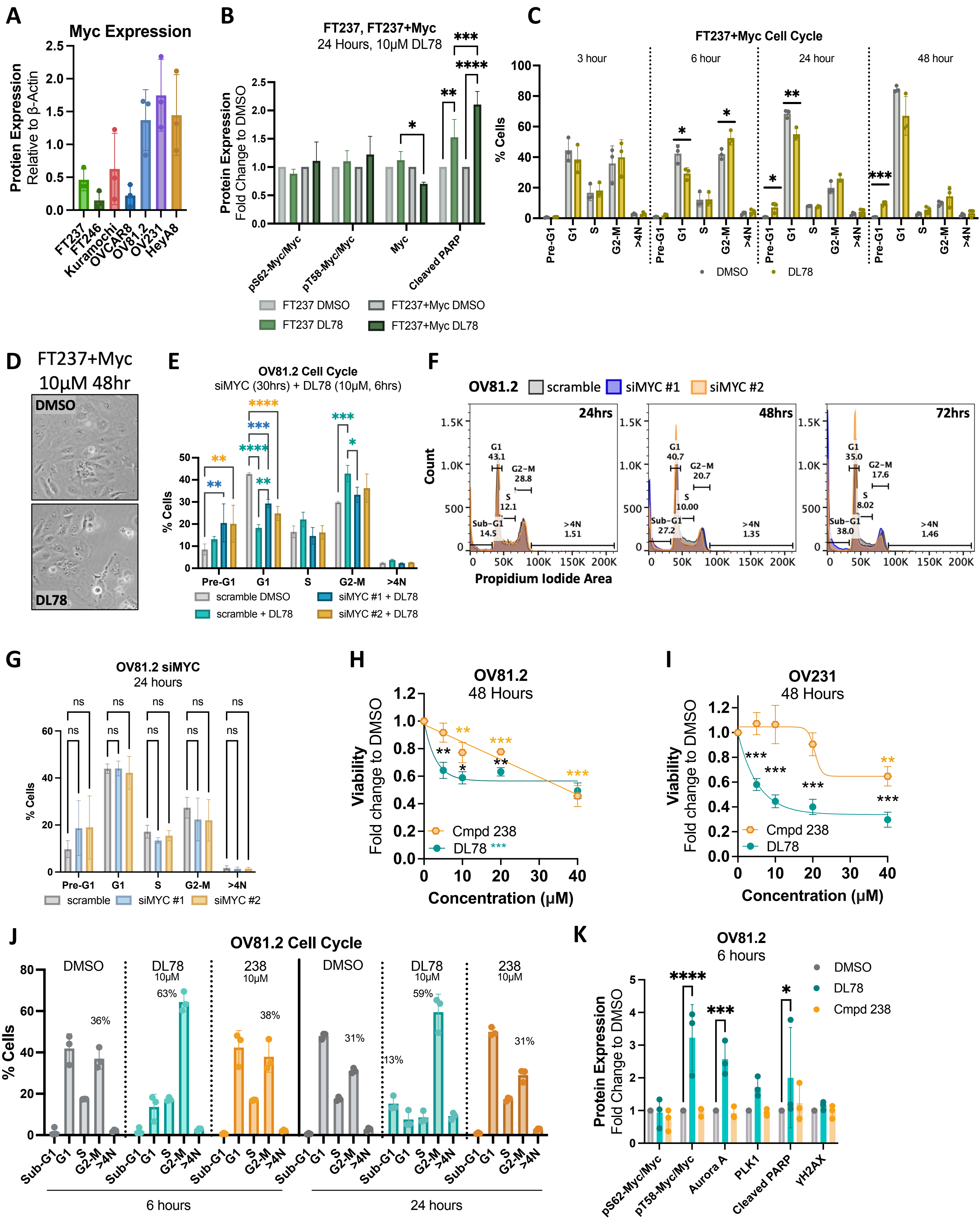

FIGURE S6. Cell cycle and western quantification.
(A) Quantification of Western blot in Figure 5B.
(B) Quantification of the Western blot in Figure 5E relative to each DMSO.
(C) Quantification of FT237+Myc cell cycle analysis in Figure 5F.
(D) 20X Microscopy image of FT237+Myc cells following 48 hour treatment of DMSO or DL78.
(E) Quantification of OV81.2 cell cycle analysis in Figure 5I.
(F) Representative histograms of flow cytometry propidium iodide cell cycle analysis in OV81.2 following 24, 48, or 72 hours of transfection with scramble, siMYC#1, or siMYC#2.
(G) Quantification of OV81.2 scramble, siMYC#1, or siMYC#2 24hr cell cycle analysis in Figure S5F.
(H) MTT in OV81.2 cells treated with DL78 or Cmpd 238 for 48 hours. Colored asterisks denote significant difference from untreated and black asterisks denote significant difference between DL78 and Cmpd 238 at that dose.
(I) Same as (H) but performed in OV231 cells.
(J) Quantification of OV81.2 cell cycle analysis in Figure 5L.
(K) Quantification of the Western blot in Figure 5M relative to DMSO.
Data plotted as mean with error bars as standard deviation, n=3 biological replicates.
*$p < 0.05$, **$p < 0.01$, and ***$p < 0.001$ as determined by two-sided Student's t-test.

Figure S7. Western blot and cell cycle analysis quantification.

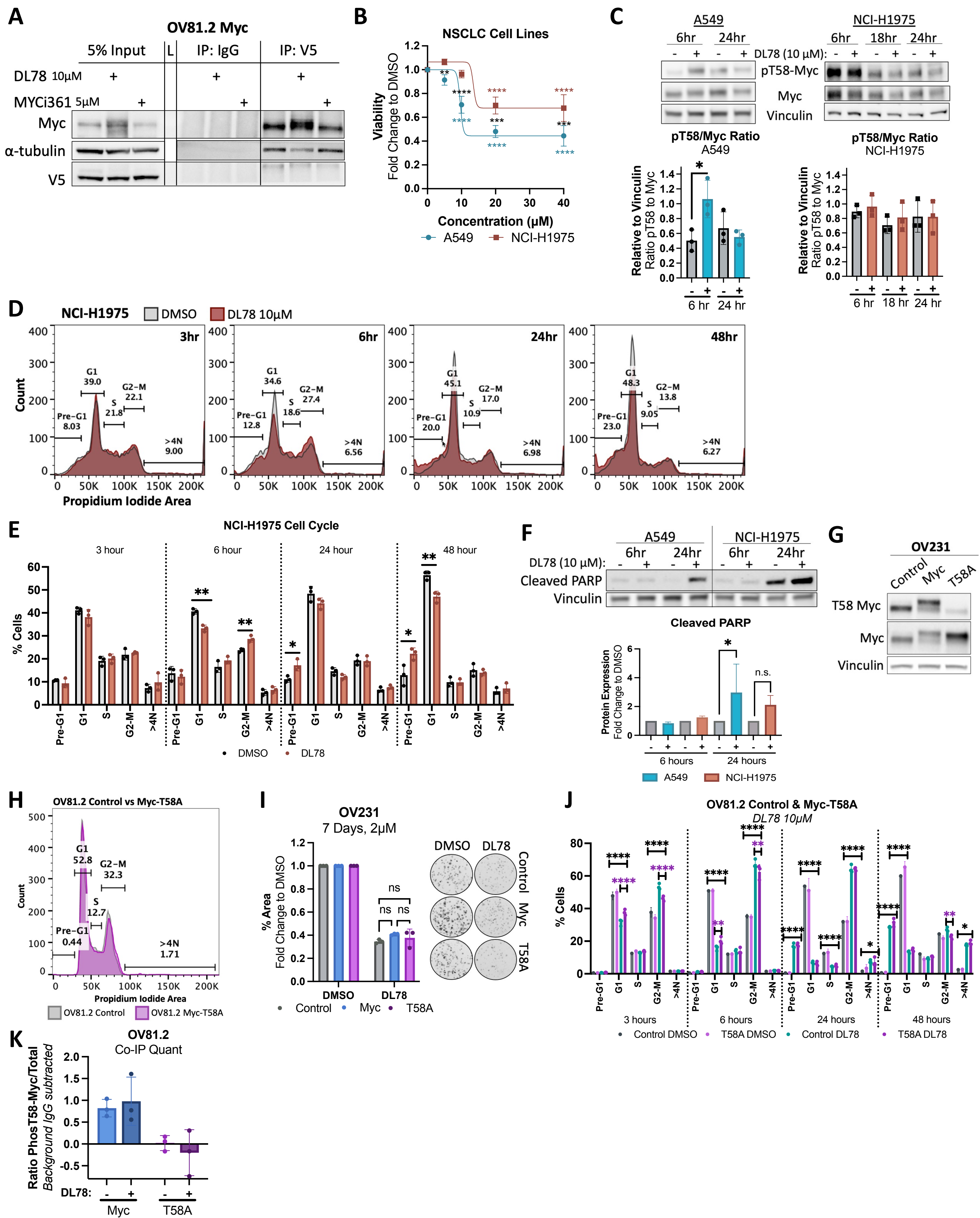

FIGURE S7. Western blot and cell cycle analysis quantification.

(A) Co-immunoprecipitation of V5-tagged Myc from OV81.2-Myc cells treated with DMSO, 10μM DL78, or 5μM MYCi361 for 6 hours.

(B) MTT assay of A549 and NCI-H1975 cells treated with DL78 (0, 5, 10, 20, 40μM) for 48 hours. Colored asterisks denote statistical significance compared to 0μM treatment. Black asterisks denote significant differences between A549 and NCI-H1975 at the respective dose.

(C) Western blot of Myc in A549 or NCI-H1975 treated with 10μM DL78 across various timepoints. Quantification below relative to DMSO at each timepoint in A549 (left) or in NCI-H1975 (right).

(D) Representative histograms from flow cytometry propidium iodide cell cycle analysis of NCI-H1975 treated with 10μM DL78 for 3, 6, 24, or 48 hours. Statistics on graph are for DL78-treated cells.

(E) Quantification of NCI-H1975 cell cycle analysis in Figure S7D.

(F) Western blot in A549 and NCI-H1975 treated with 10μM DL78 for 6 or 24 hours. Quantification below. Significance determined by one-way ANOVA.

(G) Western blot of OV231 Control, Myc, or T58A transduced cells.

(H) Representative histogram of flow cytometry propidium iodide cell cycle analysis in untreated OV81.2 Control or T58A.

(I) Colony formation in OV231 Control, Myc or T58A cells following 7 days of DMSO or 2μM DL78 treatment. Representative images are to the right of the graph.

(J) Cell cycle analysis quantification of Figure 6E. Black asterisks compare T58A DMSO vs T58A DL78 treated; purple asterisks compare Control DL78 to T58A DL78 determined by two-way ANOVA.

(K) Quantification of Co-IP in Figure 6F. Background from the IgG lanes was subtracted from the V5 pulldown lanes.

Data plotted as mean with error bars as standard deviation, n=3 biological replicates.

*$p < 0.05$, **$p < 0.01$, and ***$p < 0.001$ as determined by two-sided Student's t-test.

Figure S8. SwissADME screenshots and in vivo quants.

A

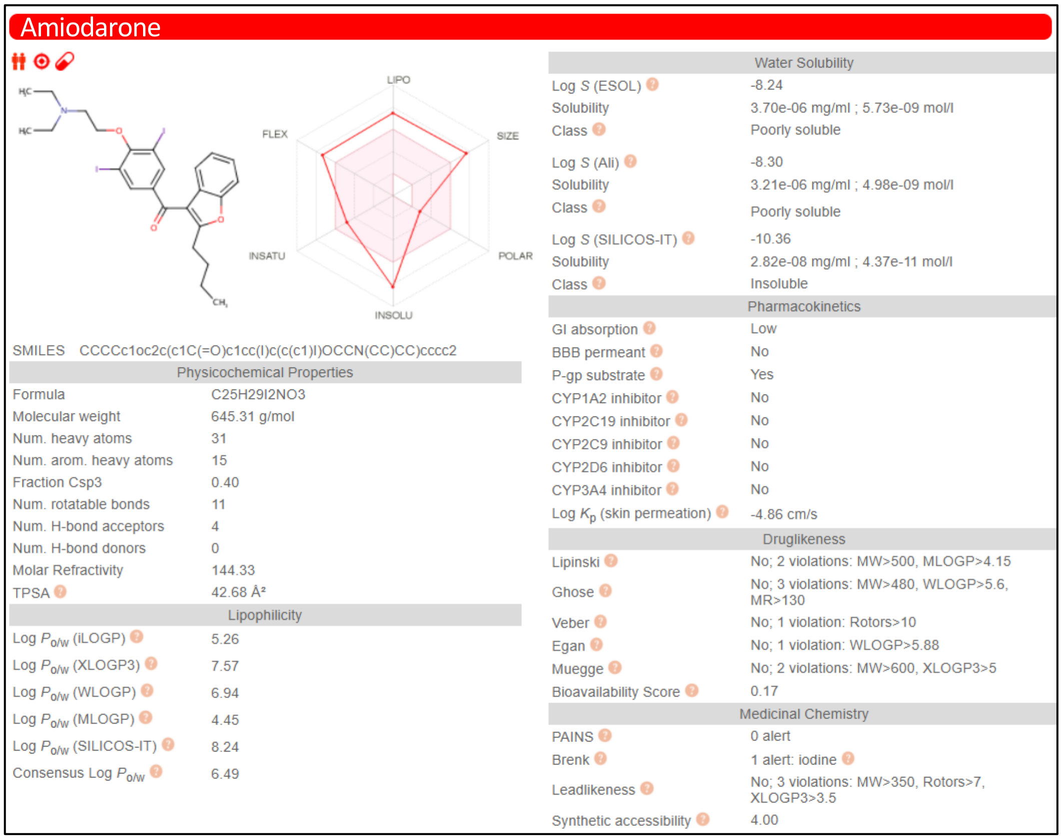

Amiodarone

SMILES CCCCc1oc2c(c1C(=O)c1cc(I)c(c(c1)I)OCCN(CC)CC)cccc2

Physicochemical Properties	
Formula	C25H29I2NO3
Molecular weight	645.31 g/mol
Num. heavy atoms	31
Num. arom. heavy atoms	15
Fraction Csp3	0.40
Num. rotatable bonds	11
Num. H-bond acceptors	4
Num. H-bond donors	0
Molar Refractivity	144.33
TPSA	42.68 Å²

Lipophilicity	
Log $P_{o/w}$ (iLOGP)	5.26
Log $P_{o/w}$ (XLOGP3)	7.57
Log $P_{o/w}$ (WLOGP)	6.94
Log $P_{o/w}$ (MLOGP)	4.45
Log $P_{o/w}$ (SILICOS-IT)	8.24
Consensus Log $P_{o/w}$	6.49

Water Solubility	
Log *S* (ESOL)	-8.24
Solubility	3.70e-06 mg/ml ; 5.73e-09 mol/l
Class	Poorly soluble
Log *S* (Ali)	-8.30
Solubility	3.21e-06 mg/ml ; 4.98e-09 mol/l
Class	Poorly soluble
Log *S* (SILICOS-IT)	-10.36
Solubility	2.82e-08 mg/ml ; 4.37e-11 mol/l
Class	Insoluble

Pharmacokinetics	
GI absorption	Low
BBB permeant	No
P-gp substrate	Yes
CYP1A2 inhibitor	No
CYP2C19 inhibitor	No
CYP2C9 inhibitor	No
CYP2D6 inhibitor	No
CYP3A4 inhibitor	No
Log K_p (skin permeation)	-4.86 cm/s

Druglikeness	
Lipinski	No; 2 violations: MW>500, MLOGP>4.15
Ghose	No; 3 violations: MW>480, WLOGP>5.6, MR>130
Veber	No; 1 violation: Rotors>10
Egan	No; 1 violation: WLOGP>5.88
Muegge	No; 2 violations: MW>600, XLOGP3>5
Bioavailability Score	0.17

Medicinal Chemistry	
PAINS	0 alert
Brenk	1 alert: iodine
Leadlikeness	No; 3 violations: MW>350, Rotors>7, XLOGP3>3.5
Synthetic accessibility	4.00

B

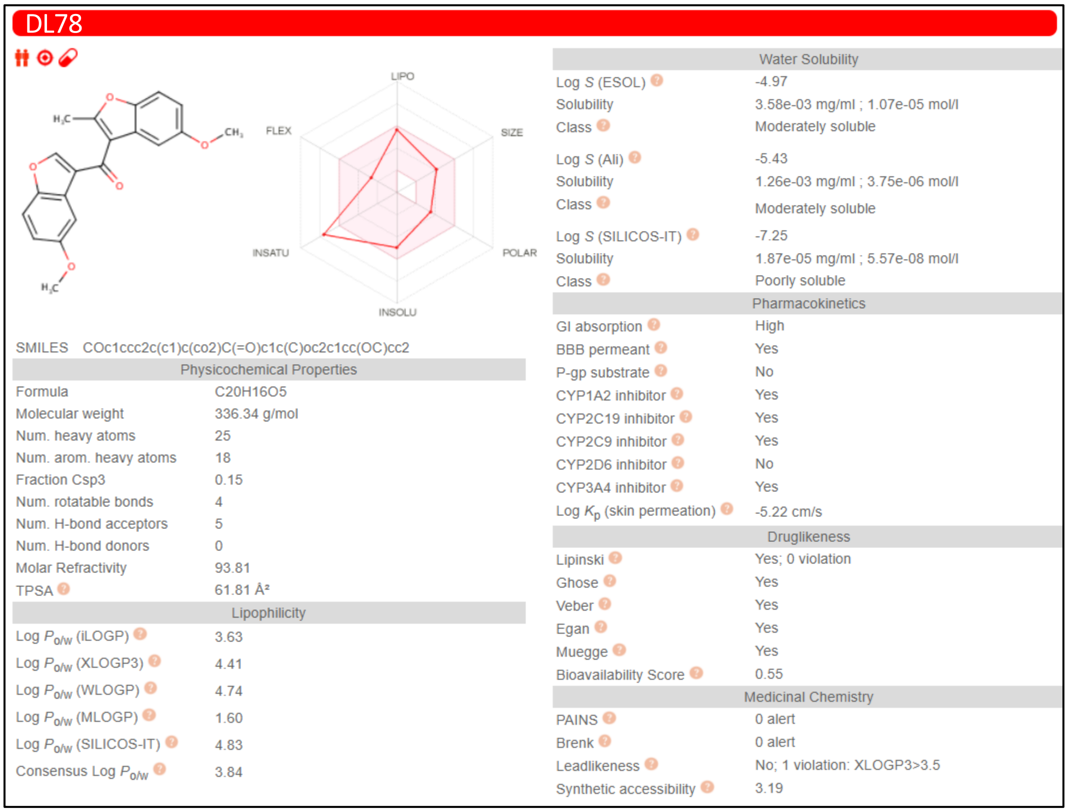

DL78

SMILES COc1ccc2c(c1)c(co2)C(=O)c1c(C)oc2c1cc(OC)cc2

Physicochemical Properties	
Formula	C20H16O5
Molecular weight	336.34 g/mol
Num. heavy atoms	25
Num. arom. heavy atoms	18
Fraction Csp3	0.15
Num. rotatable bonds	4
Num. H-bond acceptors	5
Num. H-bond donors	0
Molar Refractivity	93.81
TPSA	61.81 Å²

Lipophilicity	
Log $P_{o/w}$ (iLOGP)	3.63
Log $P_{o/w}$ (XLOGP3)	4.41
Log $P_{o/w}$ (WLOGP)	4.74
Log $P_{o/w}$ (MLOGP)	1.60
Log $P_{o/w}$ (SILICOS-IT)	4.83
Consensus Log $P_{o/w}$	3.84

Water Solubility	
Log *S* (ESOL)	-4.97
Solubility	3.58e-03 mg/ml ; 1.07e-05 mol/l
Class	Moderately soluble
Log *S* (Ali)	-5.43
Solubility	1.26e-03 mg/ml ; 3.75e-06 mol/l
Class	Moderately soluble
Log *S* (SILICOS-IT)	-7.25
Solubility	1.87e-05 mg/ml ; 5.57e-08 mol/l
Class	Poorly soluble

Pharmacokinetics	
GI absorption	High
BBB permeant	Yes
P-gp substrate	No
CYP1A2 inhibitor	Yes
CYP2C19 inhibitor	Yes
CYP2C9 inhibitor	Yes
CYP2D6 inhibitor	No
CYP3A4 inhibitor	Yes
Log K_p (skin permeation)	-5.22 cm/s

Druglikeness	
Lipinski	Yes; 0 violation
Ghose	Yes
Veber	Yes
Egan	Yes
Muegge	Yes
Bioavailability Score	0.55

Medicinal Chemistry	
PAINS	0 alert
Brenk	0 alert
Leadlikeness	No; 1 violation: XLOGP3>3.5
Synthetic accessibility	3.19

C

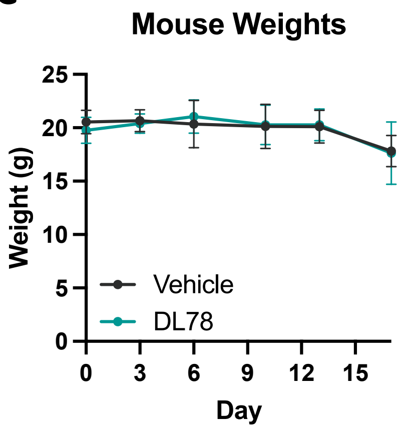

FIGURE S8. SwissADME screenshots and western quantification.
(A) Screenshot from SwissADME of *in silico* bioavailability analysis of amiodarone showcasing the details used to consider its drug-likeness qualities.
(B) Screenshot from SwissADME of *in silico* bioavailability analysis of DL78 showcasing the details used to consider its drug-likeness qualities.
(C) Mouse body weights in grams.

SUPPLEMENTAL TABLES

Table S1: COMPARE database results, correlating DL78's percent growth pattern with other microtubule-targeting drugs tested in the NCI-60 Screen. MT = microtubule; ER = estrogen receptor

Dataset	Correlation	Count of Cell Lines Included	Target Descriptor	Mechanism of Action
BEC_Referral_Set_GI50	0.68	58	BAVISTIN	MT Destabilizer
	0.66	59	(-)-EBURNAMONINE	Alkaloid
	0.64	59	CLANFENUR (INN)	MT Inhibitor
	0.63	55	INDIBULIN	MT Inhibitor
	0.64	58	5-IODO-2-(2-PHENOXYACETAMIDO) BENZAMIDE	MT Inhibitor Derivative
Marketed_Drugs_GI50	0.53	48	TAXOTERE	MT Stabilizer
	0.42	59	RP 54780	Alkylating Agent
	0.39	57	TAMOXIFEN	ER modulator
Marketed_Drugs_TGI	0.50	60	PACLITAXEL	MT Stabilizer
	0.46	60	TAMOXIFEN	ER Modulator
	0.44	57	TAXOTERE	MT Stabilizer
	0.40	59	VINBLASTINE SULFATE	MT Inhibitor

Table S2: Compound Molport IDs graphed in Figure 1A.

Alias	Molport ID
Compound 3	MolPort-001-937-649
DL78	MolPort-002-508-326
Compound 17	Molport-003-943-318
Compound 36	Molport-000-005-342
Compound 238	Molport-002-140-494
	Molport-002-549-957
	Molport-002-547-424
	Molport-002-622-240
	Molport-002-083-833
	Molport-002-546-048
	Molport-002-692-375
	Molport-002-134-160
	Molport-002-508-589
	Molport-002-175-444
	Molport-001-964-428
	Molport-008-700-583
	Molport-035-814-304
	MFCD03839860

	Molport-001-972-500
	Molport-046-762-162
	PHO10642
	PHO11016

Table S3: Antibodies used in Western blotting.

Name	Supplier	Catalog Number
α-tubulin	Cell Signaling Technology	3873
Aurora A	Cell Signaling Technology	12100
pT58-Myc	Applied Biological Materials Inc.	Y011034
pS62-Myc	Abcam	ab78318
c-Myc	Cell Signaling Technology	9402
pS216-Cdc25c	Cell Signaling Technology	4901
Cdc25c	Cell Signaling Technology	4688
pY15-CDK1	Cell Signaling Technology	9111
pT161-CDK1	Cell Signaling Technology	9114
CDK1	Cell Signaling Technology	9116
pT160-CDK2	Cell Signaling Technology	2561
CDK2	Cell Signaling Technology	2546
Cleaved Caspase 3	Cell Signaling Technology	9661
Cleaved PARP	Cell Signaling Technology	9541
Cyclin A2	Cell Signaling Technology	4656
pS133-Cyclin B1	Cell Signaling Technology	4133
Cyclin B1	Cell Signaling Technology	4135
Cyclin E1	Cell Signaling Technology	4129
E2F-1	Cell Signaling Technology	3742
pT202/Y204-ERK1/2	Cell Signaling Technology	4370
ERK1/2	Cell Signaling Technology	9102
pS9-GSK3β	Abcam	ab54537
pY216/Y279-GSK3β	Invitrogen	44-604G
GSK3β	Cell Signaling Technology	9315
p21	Cell Signaling Technology	2947
pT210-PLK1	Cell Signaling Technology	5472
PLK1	Cell Signaling Technology	4513
V5	Bethyl	A190-120A
Vinculin	Santa Cruz Biotechnology	sc-73614
Wee1	Cell Signaling Technology	13084
pS139-γH2AX	Cell Signaling Technology	9718

VMCC-XG-016-071_PROTON_01

^{1}H NMR (499 MHz, cdcl$_3$) δ 7.53 – 7.47 (m, 2H), 7.37 (d, J = 9.0 Hz, 1H), 7.19 (d, J = 2.6 Hz, 1H), 7.15 – 7.09 (m, 2H), 6.94 – 6.87 (m, 1H), 3.87 (d, J = 0.8 Hz, 3H), 3.78 (d, J = 0.8 Hz, 3H), 2.64 (d, J = 0.8 Hz, 3H).

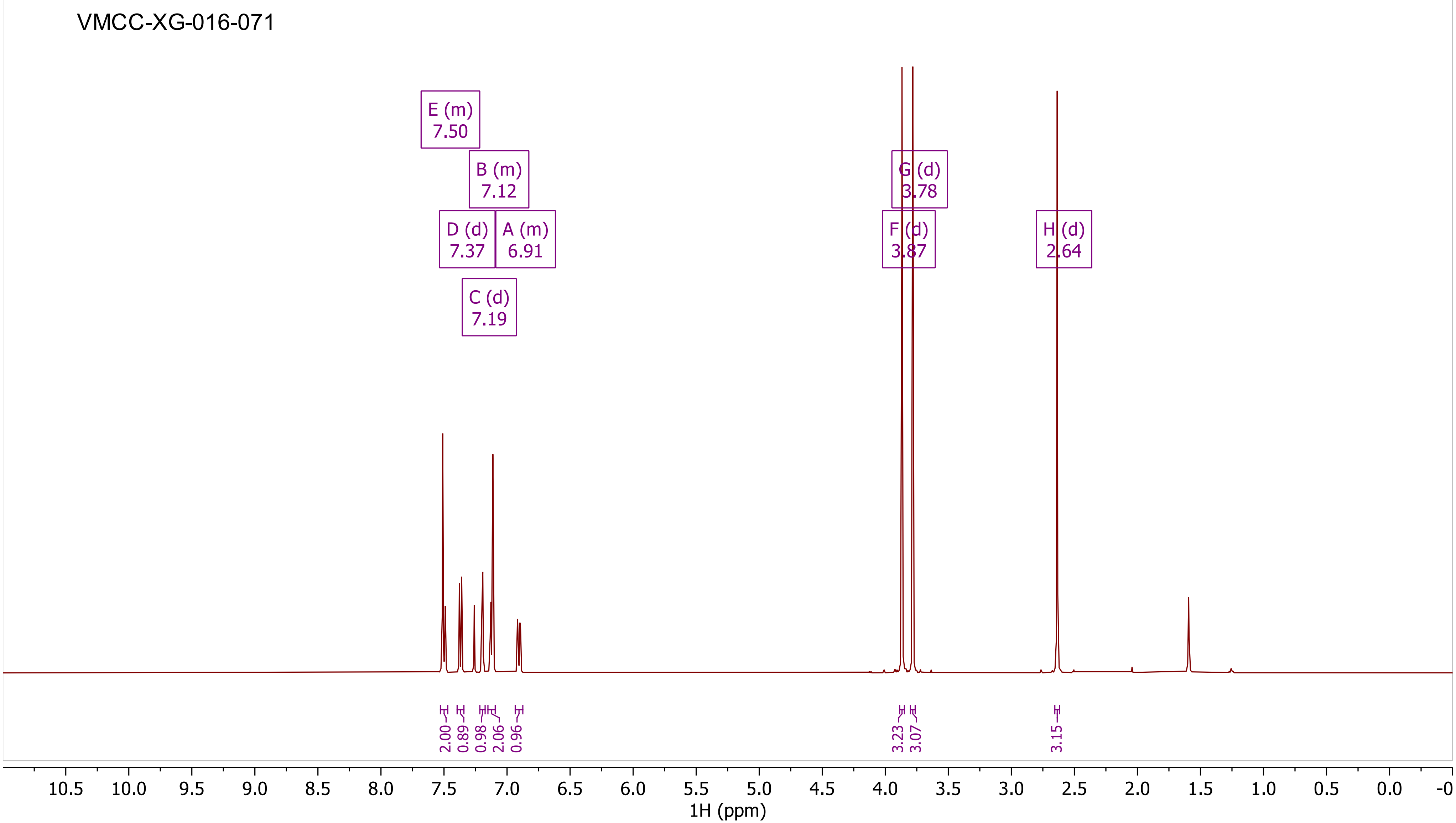

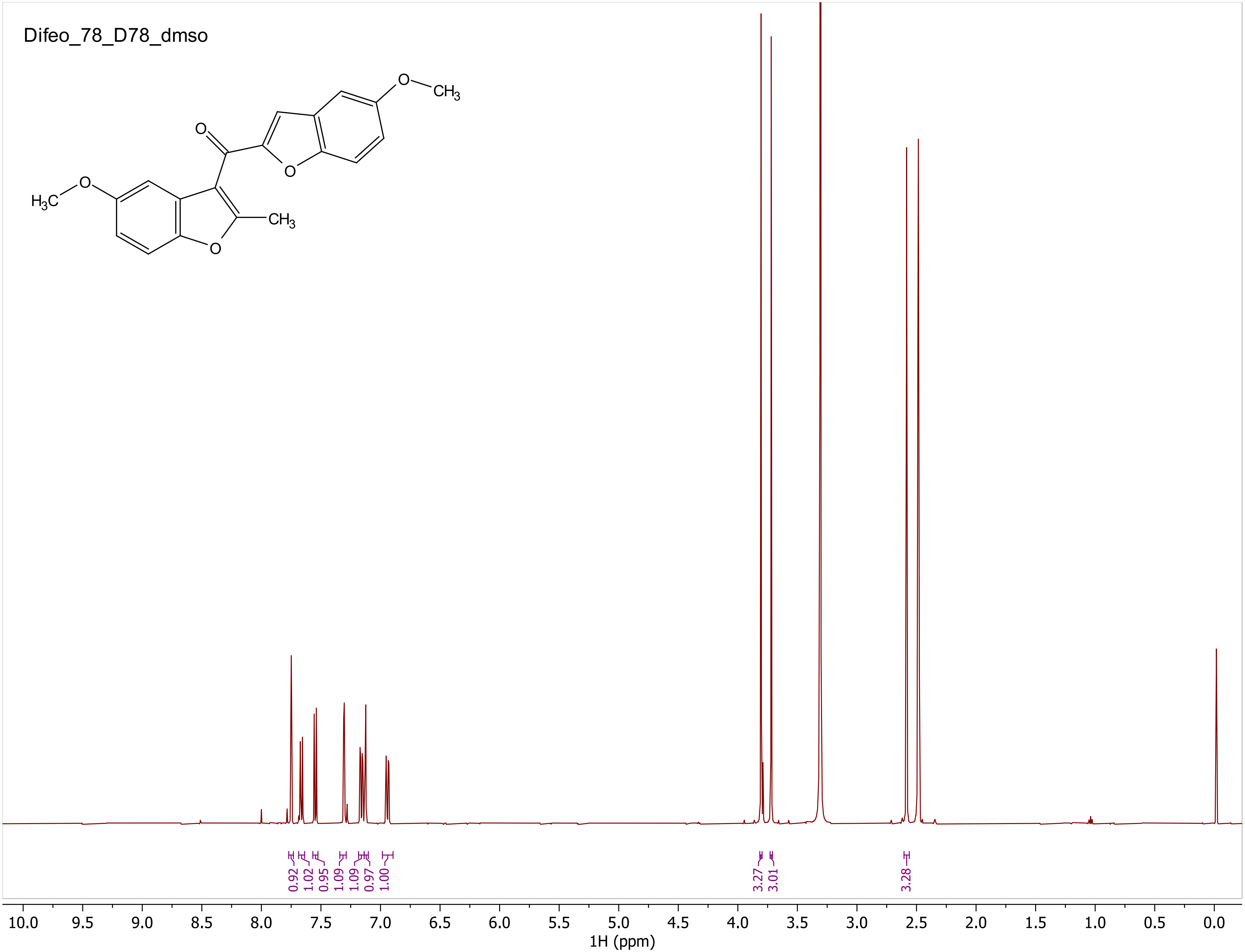

Difeo_78_D78_dmso
O
O
O
O
H3C
CH3
CH3
0.92
1.02
0.95
1.09
1.09
0.97
1.00
3.27
3.01
3.28
10.0 9.5 9.0 8.5 8.0 7.5 7.0 6.5 6.0 5.5 5.0 4.5 4.0 3.5 3.0 2.5 2.0 1.5 1.0 0.5 0.0
1H (ppm)

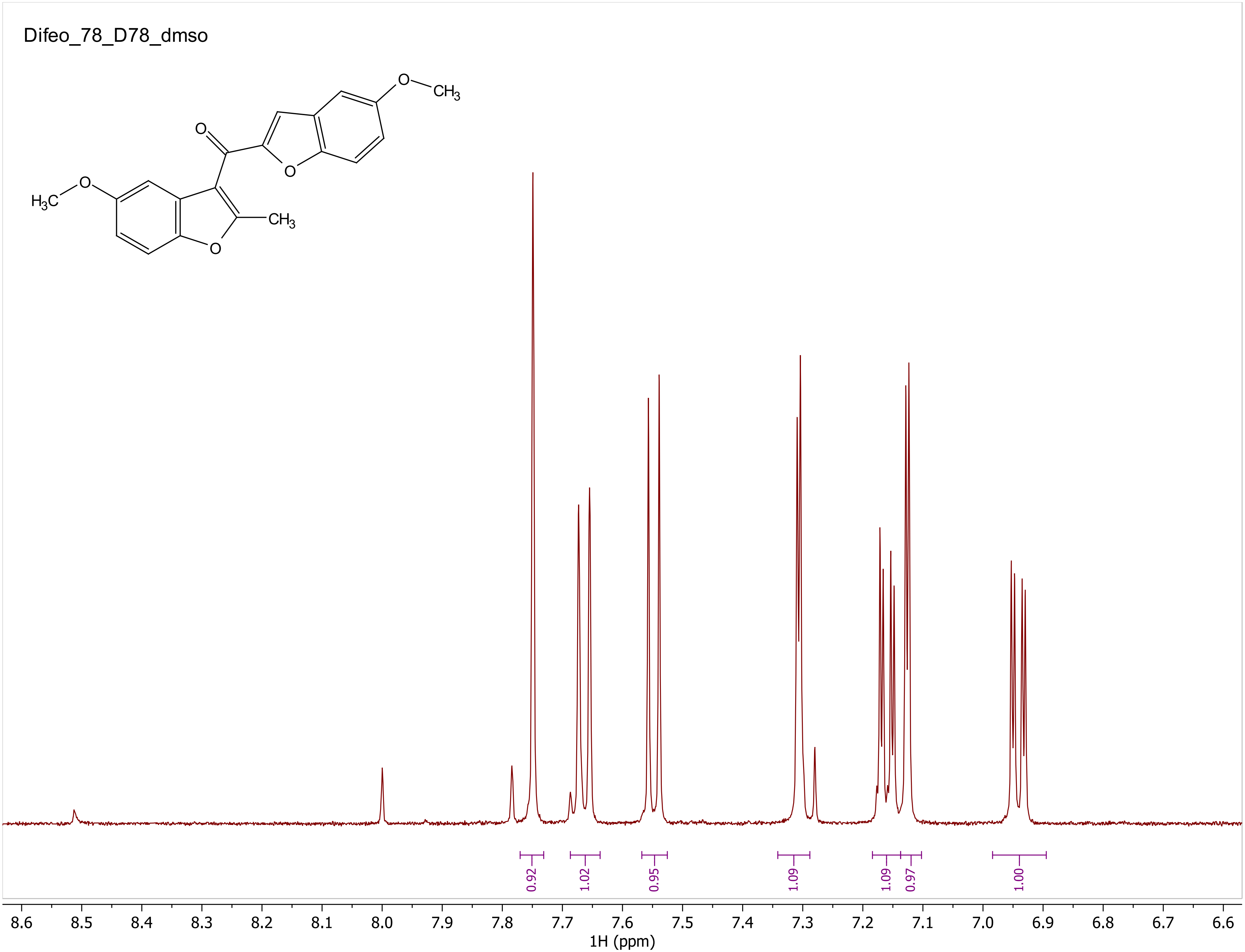
Difeo_78_D78_dmso
O
CH3
H3C
O
O
O
O
CH3
0.92
1.02
0.95
1.09
1.09
0.97
1.00
8.6 8.5 8.4 8.3 8.2 8.1 8.0 7.9 7.8 7.7 7.6 7.5 7.4 7.3 7.2 7.1 7.0 6.9 6.8 6.7 6.6
1H (ppm)

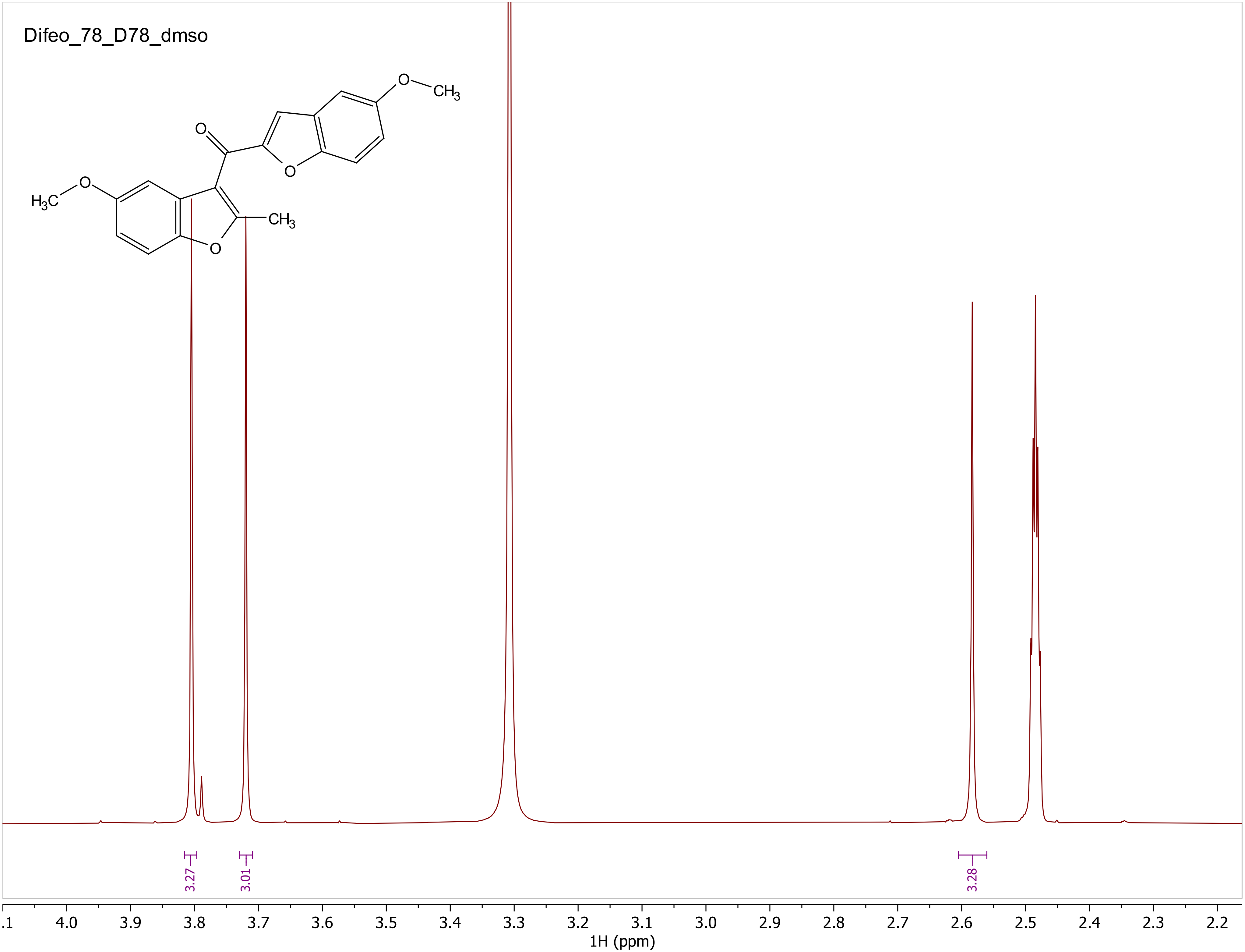
Difeo_78_D78_dmso
3.27
3.01
3.28
.1
4.0
3.9
3.8
3.7
3.6
3.5
3.4
3.3
3.2
3.1
3.0
2.9
2.8
2.7
2.6
2.5
2.4
2.3
2.2
1H (ppm)

This spectrum was provided by MolPort via email

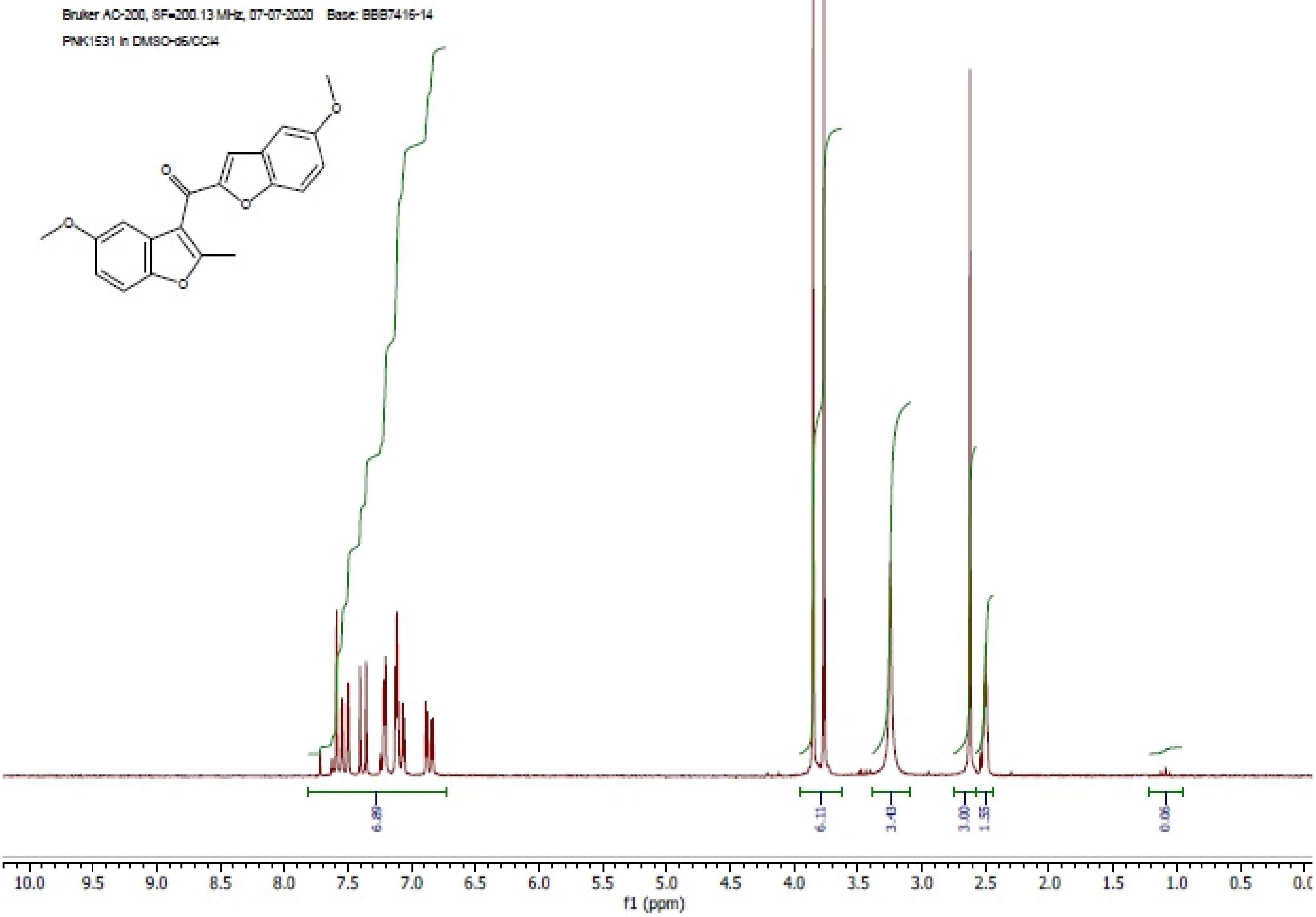

Figure 1D

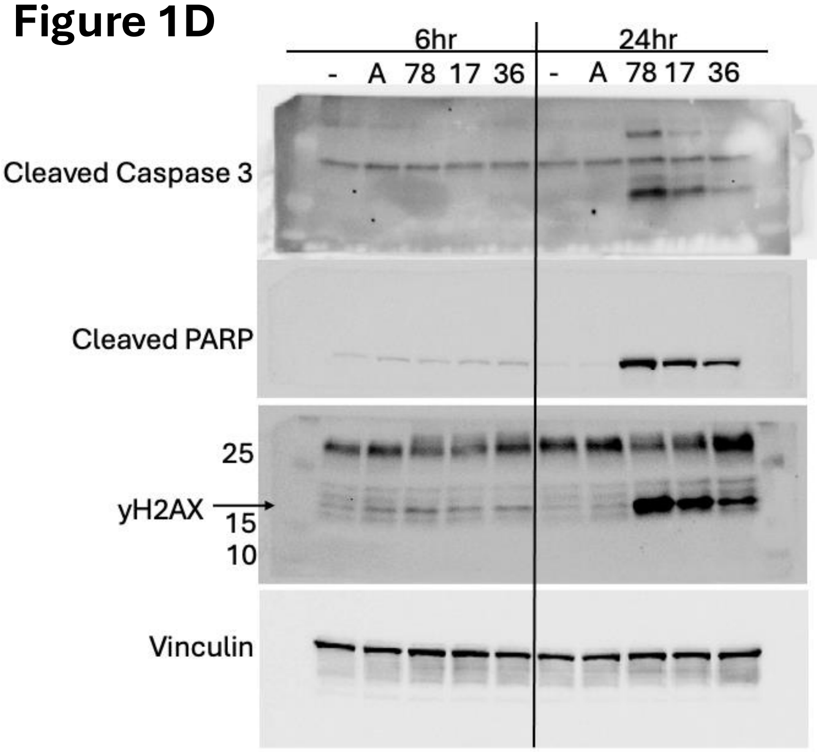

Figure 2B

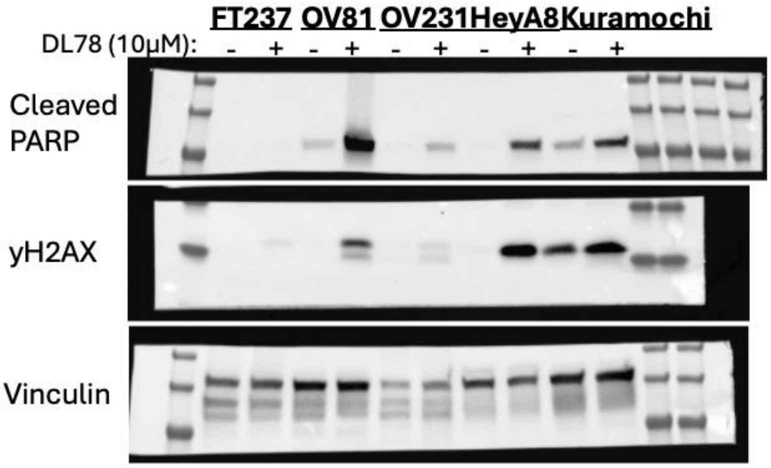

Figure 2F

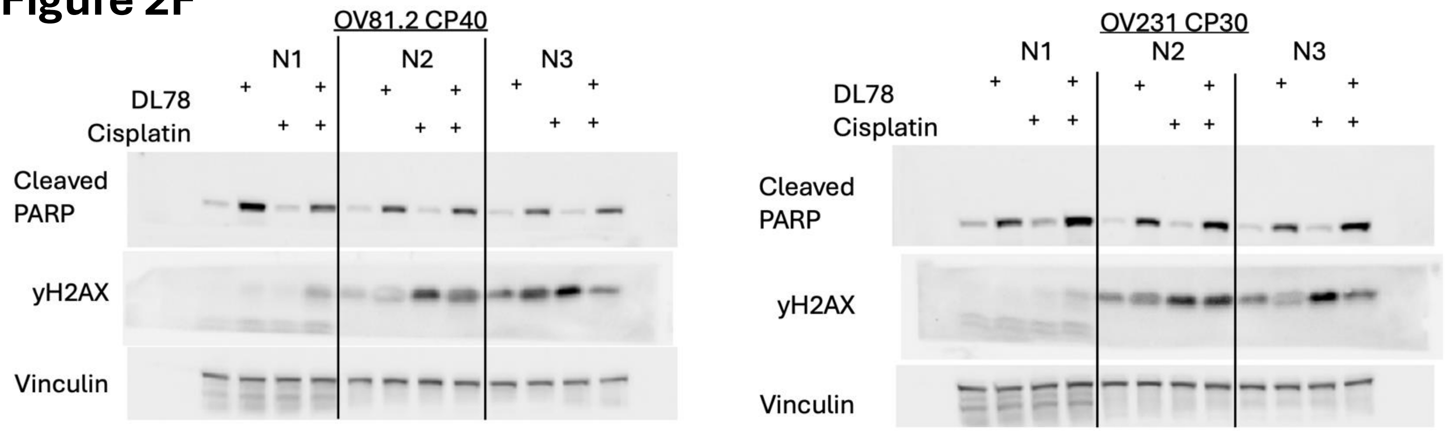

Figure 3E

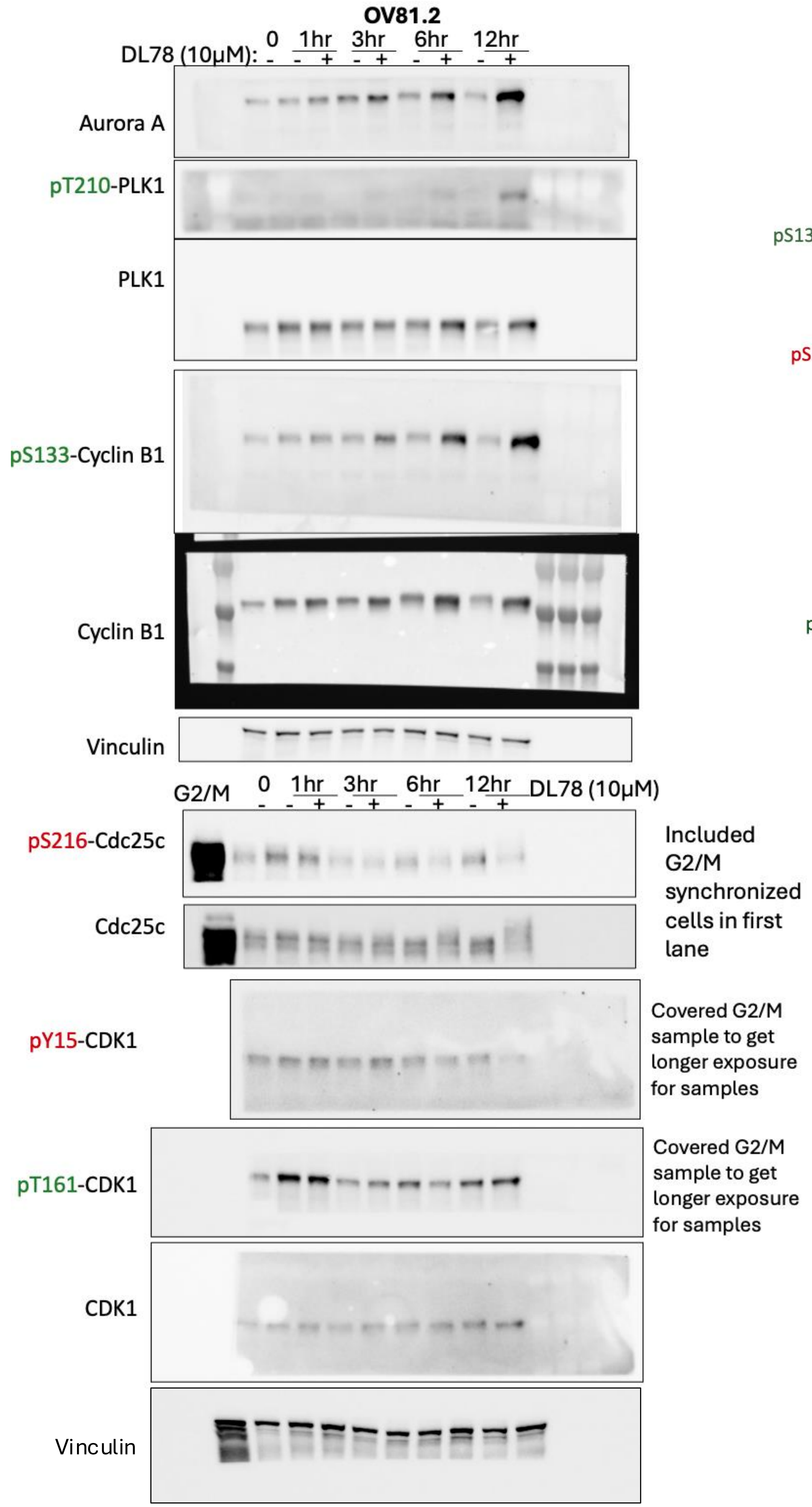

Figure 3F

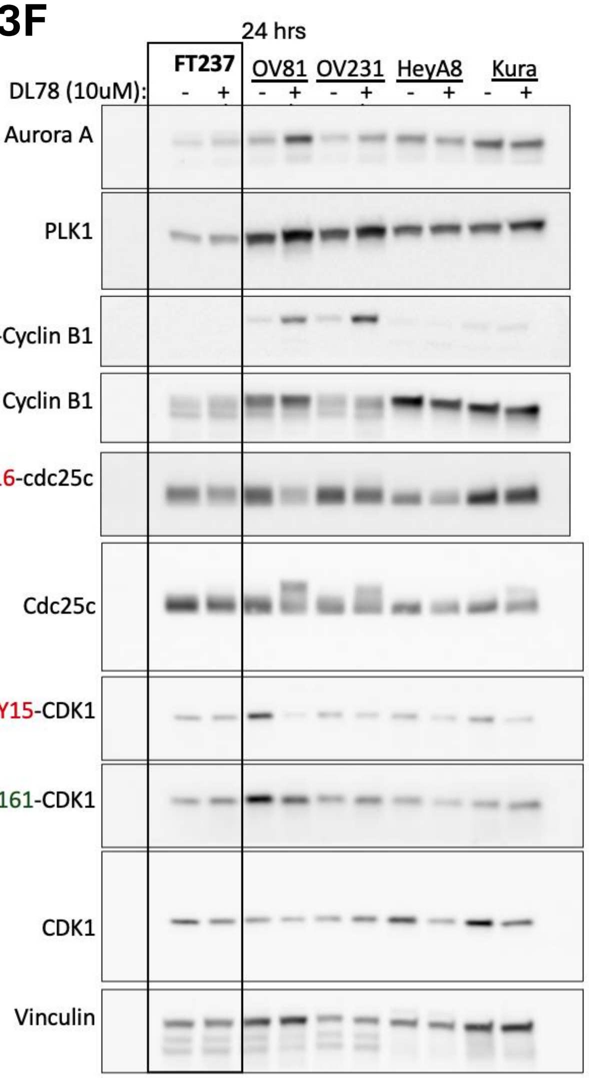

Figure 4A

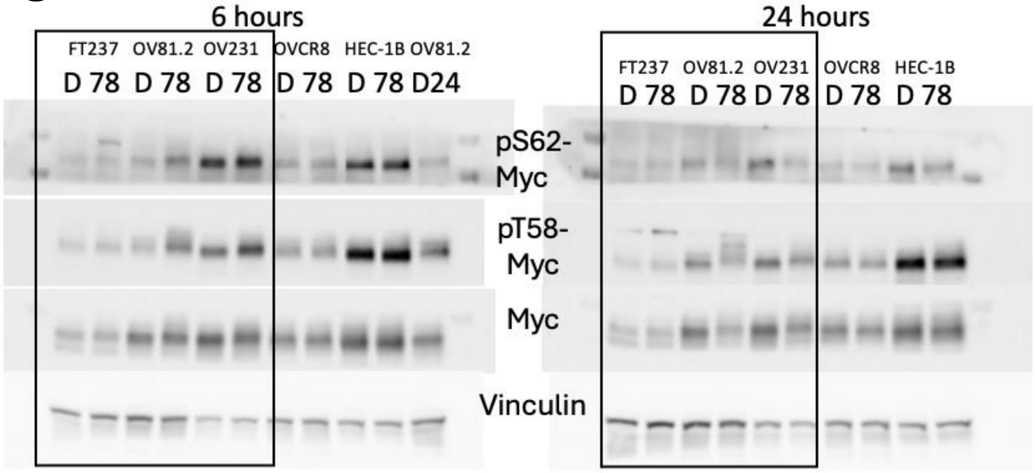

Figure 5B

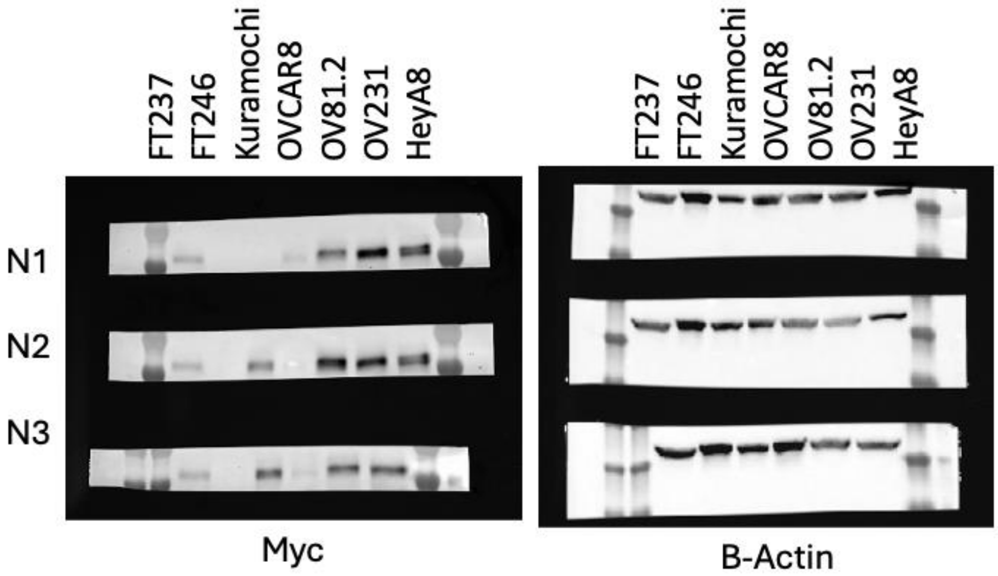

Figure 5E

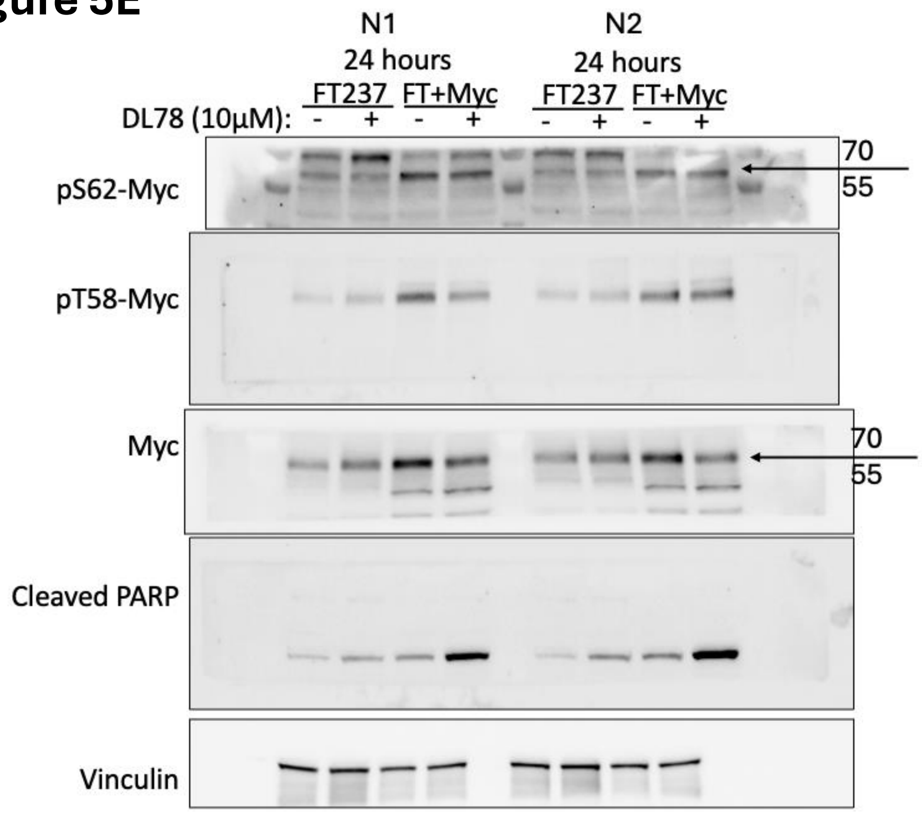

Figure 5M

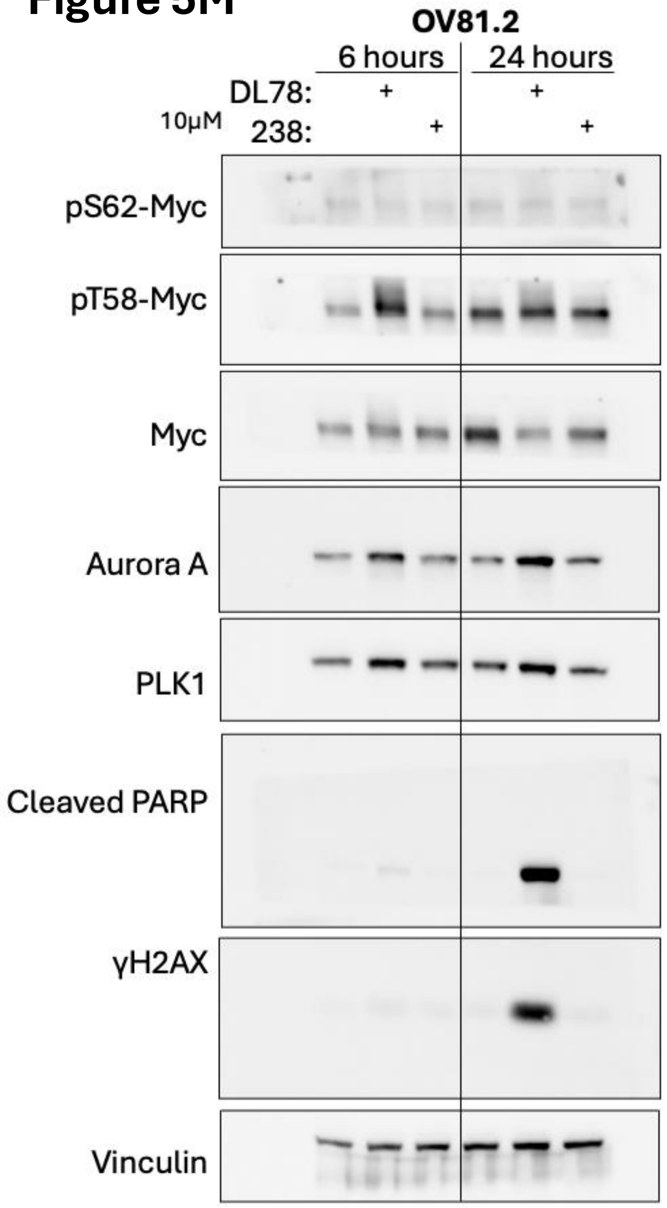

Figure 6B

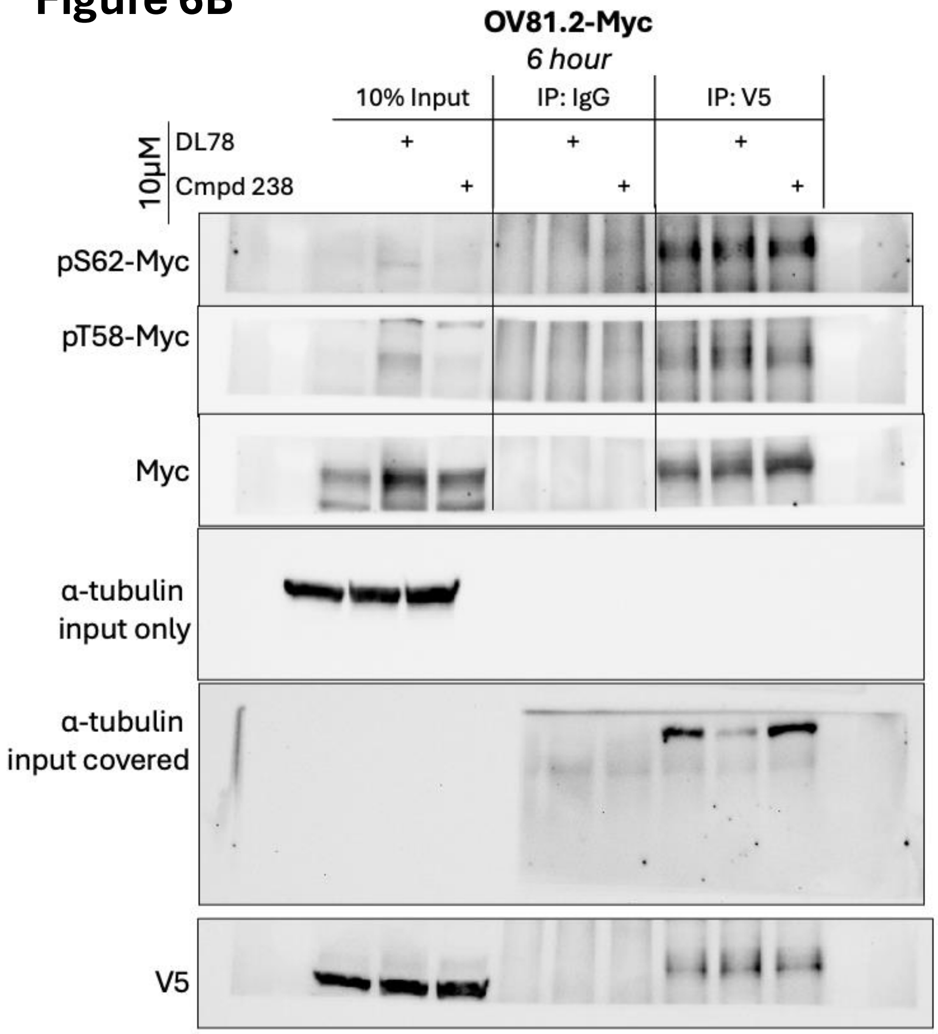

Figure 6C

Figure S7G

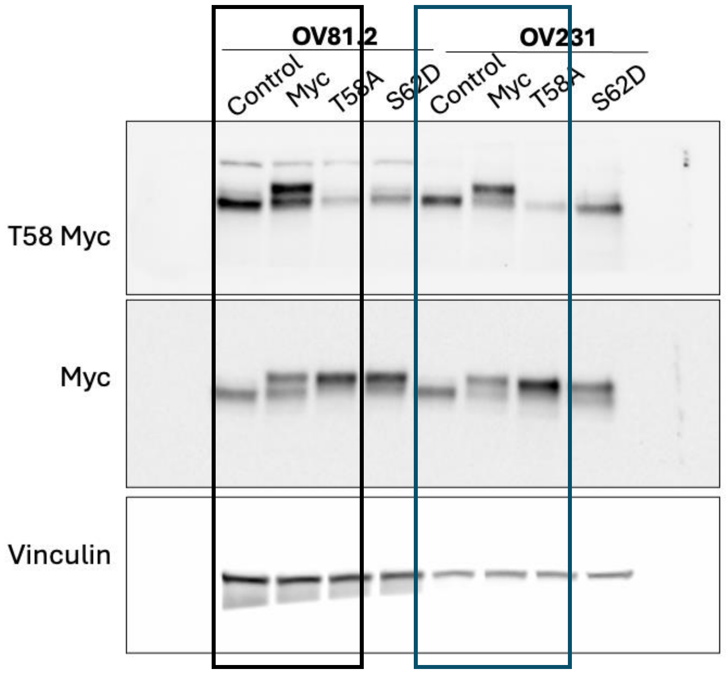

Figure 6F

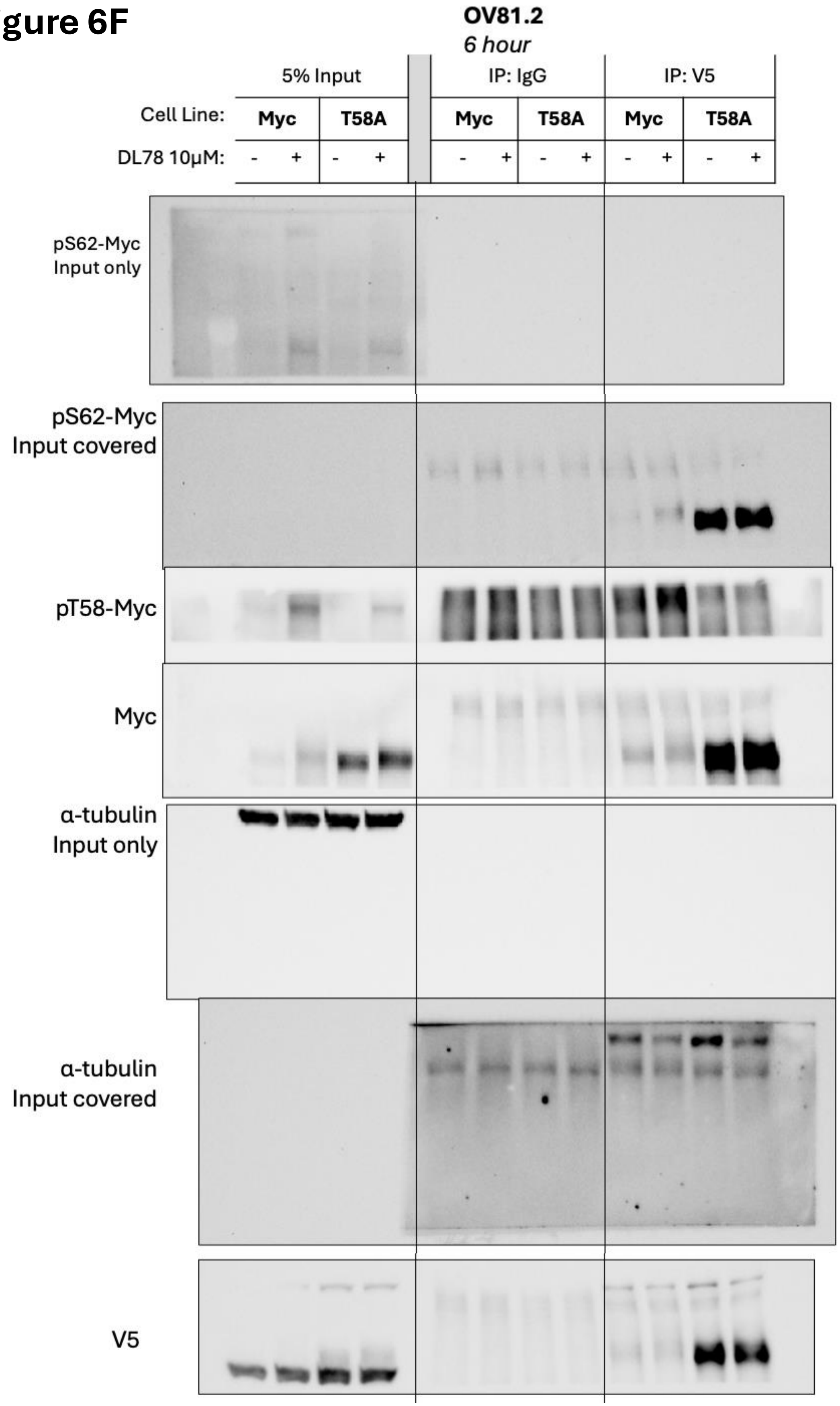
OV81.2
6 hour
5% Input
IP: IgG
IP: V5
Cell Line:
Myc
T58A
Myc
T58A
Myc
T58A
DL78 10μM:
- + - + - + - + - + - +
pS62-Myc
Input only
pS62-Myc
Input covered
pT58-Myc
Myc
α-tubulin
Input only
α-tubulin
Input covered
V5

Figure 7F

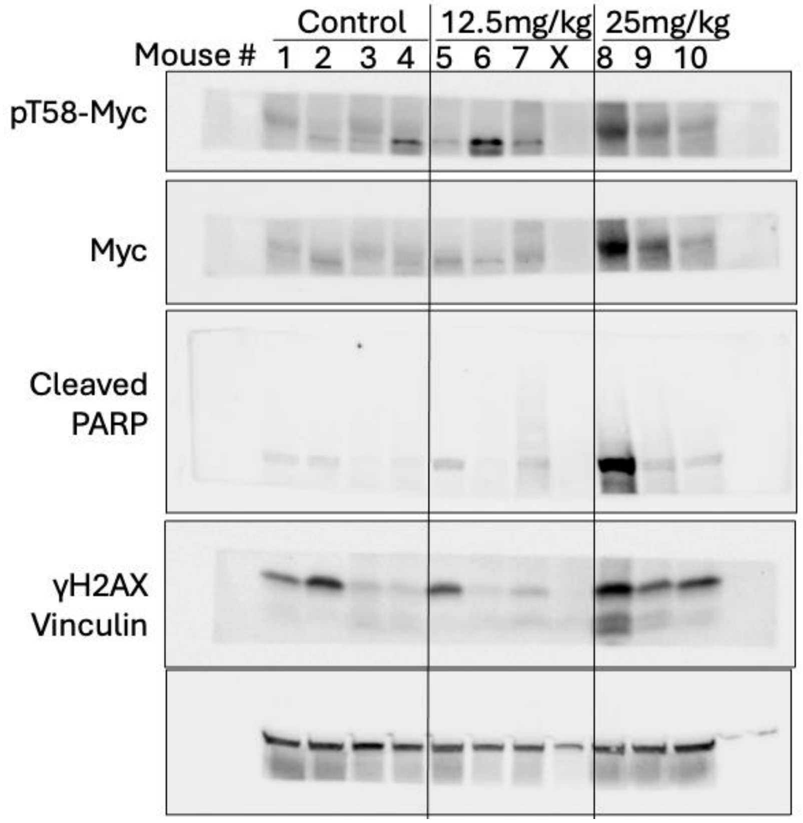

Figure S3I

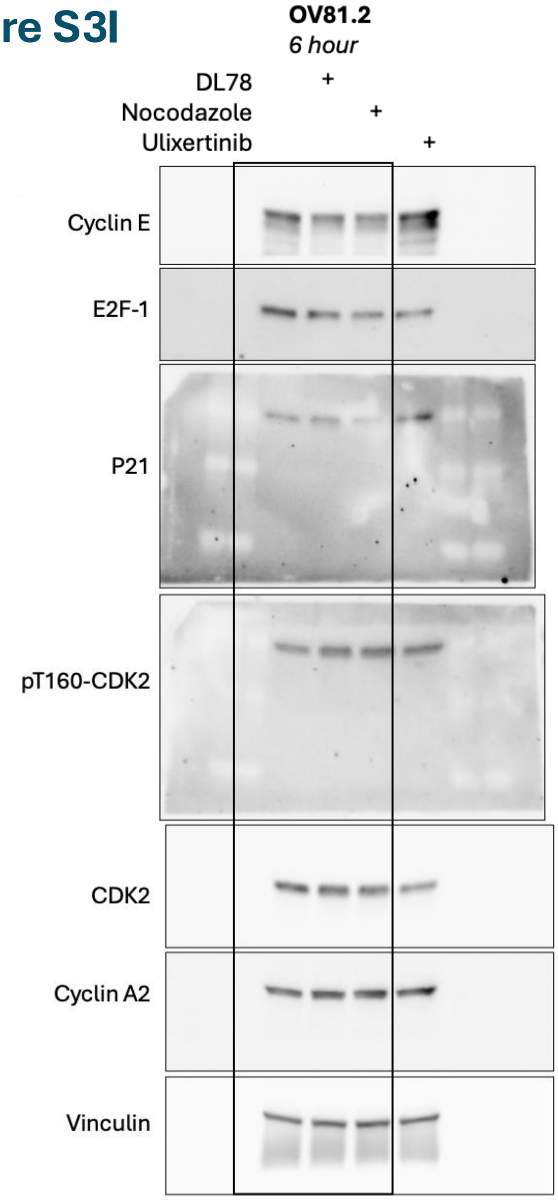

Figure S3J

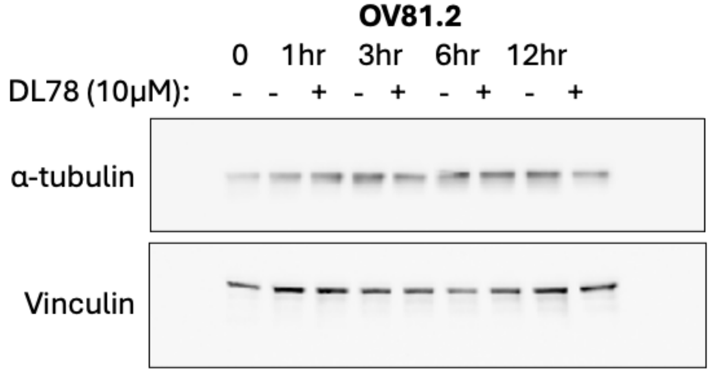

Figure S4C

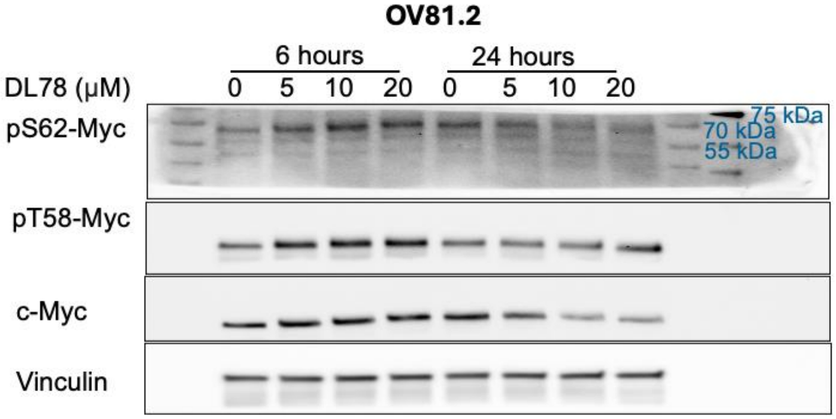

Figure S4D

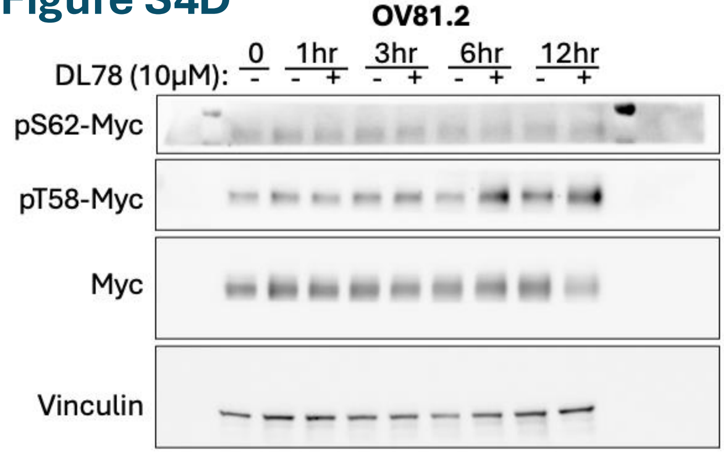

Figure S4E

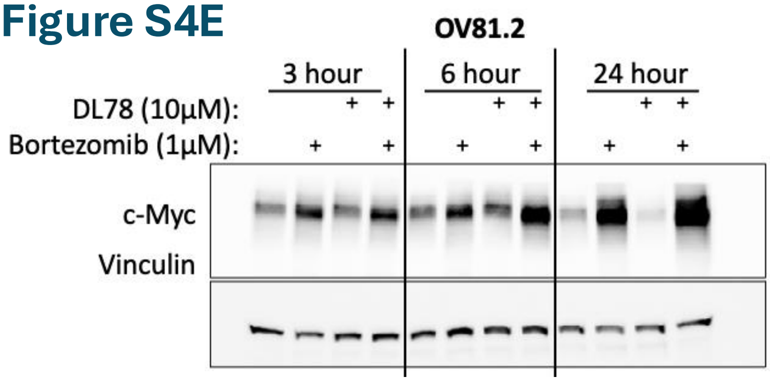

Figure S4F

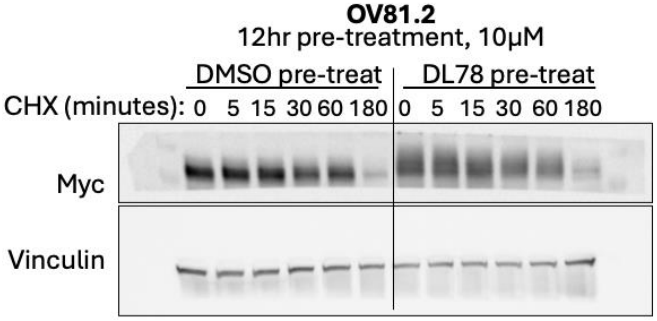

Figure S5C

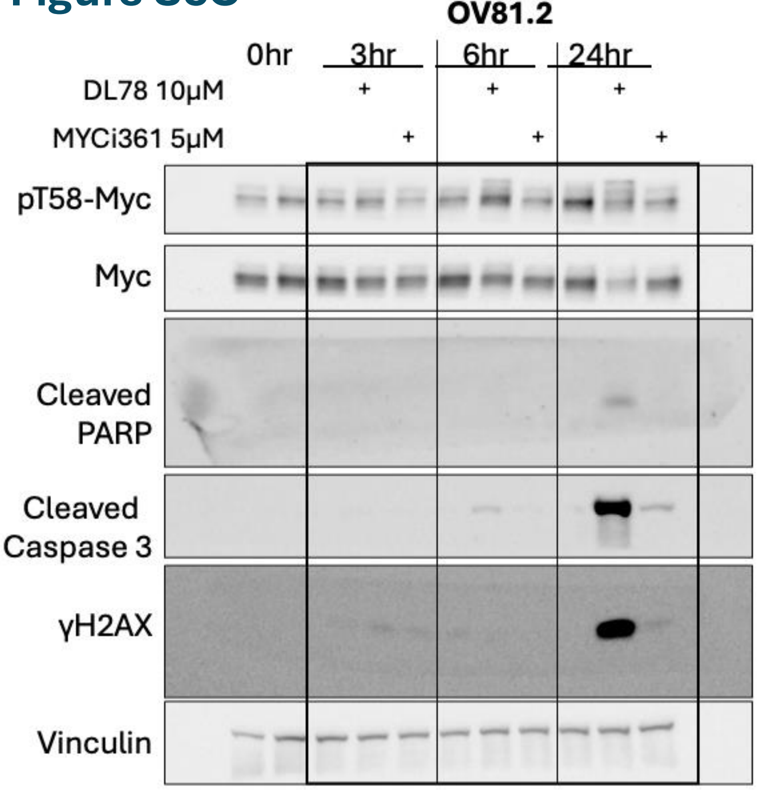

Figure S5I

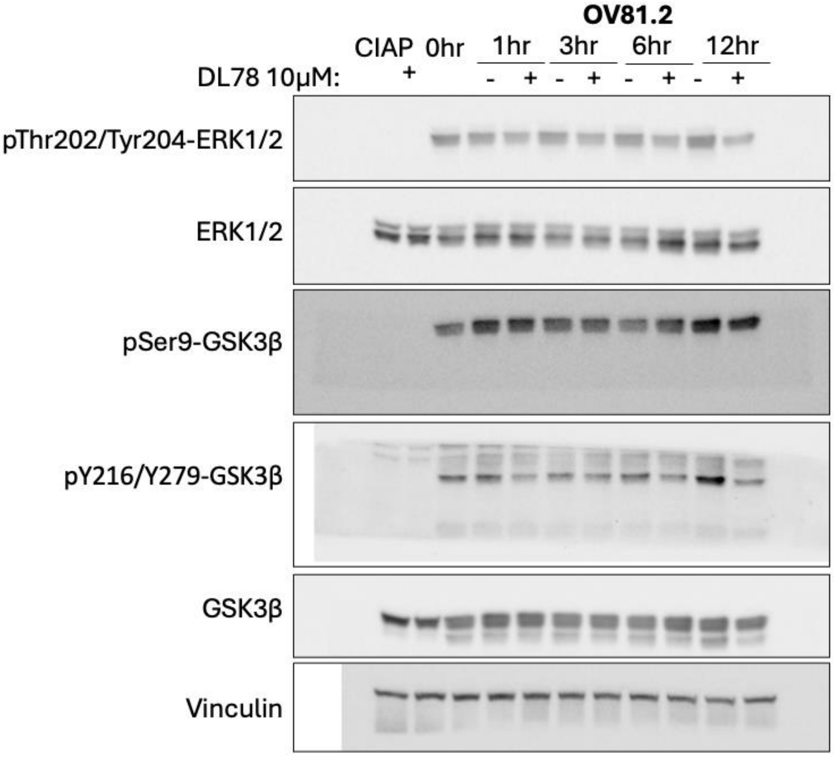

Figure S7A

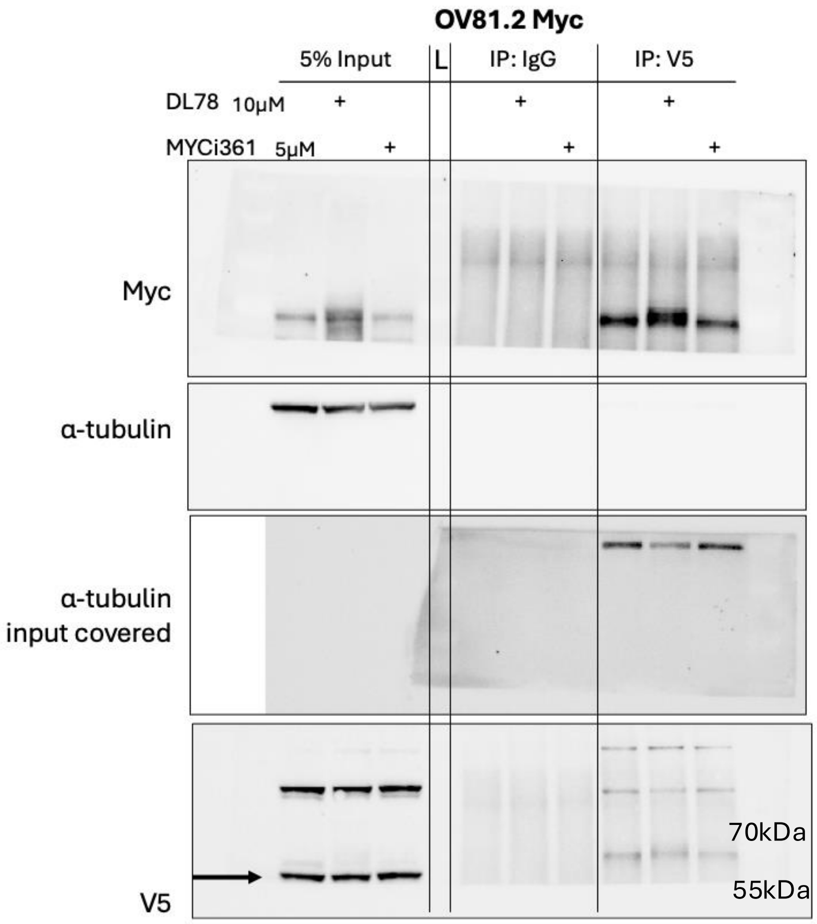

Figure S7C

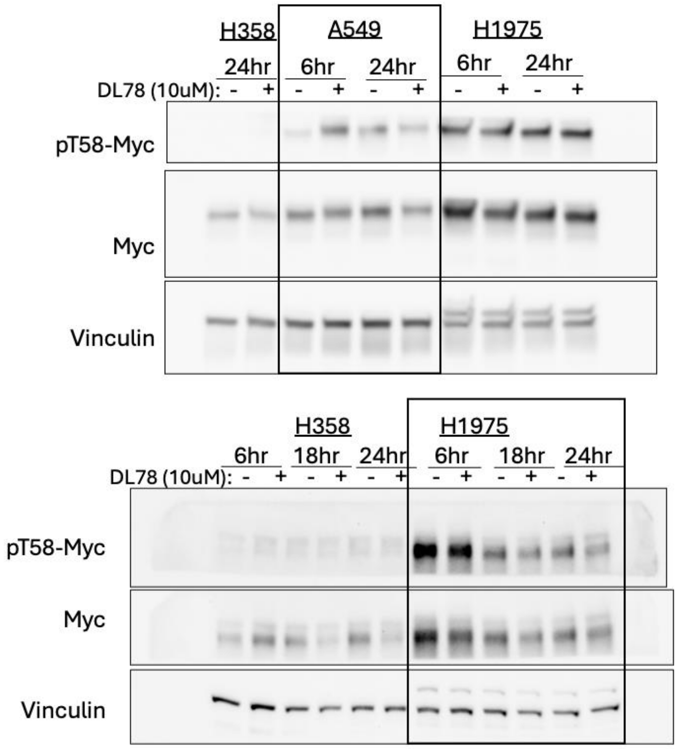

Figure S7F

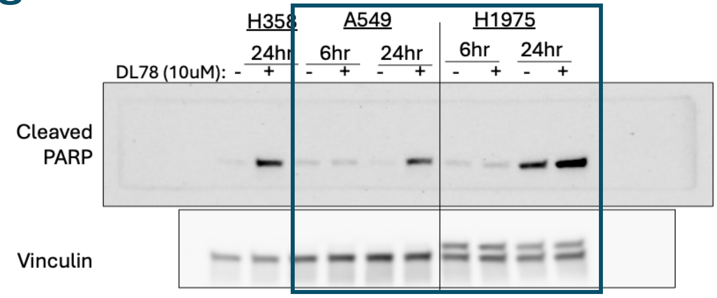

Supplement: Supplementary file 1 — Supplementary Material 1 [file 41598_2025_22011_MOESM1_ESM.pdf]
